# Whole genomic analysis of two potential recombinant strains within Human mastadenovirus species C previously found in Beijing, China

**DOI:** 10.1038/s41598-017-15336-2

**Published:** 2017-11-13

**Authors:** Naiying Mao, Zhen Zhu, Pierre Rivailler, Meng Chen, Qin Fan, Fang Huang, Wenbo Xu

**Affiliations:** 1 WHO WPRO Regional Reference Measles/Rubella Laboratory and Key Laboratory of Medical Virology Ministry of Health, National Institute for Viral Disease Control and Prevention, Chinese Centre for Disease Control and Prevention, No. 155, Changbai Road, Changping District, Beijing, 102206 People’s Republic of China; 2 Beijing Centers for Disease Control and Prevention, No. 16, Hepingli Middle Street, Dongcheng district, Beijing, 100013 People’s Republic of China

## Abstract

Human mastadenovirus species C (HAdV-C) are the most common etiologic agents of respiratory disease in young children and are frequently detected worldwide including China. Two recombinant HAdV-C strains (BJ04 and BJ09) were isolated from infants with acute respiratory infection (ARI) in Beijing in 2012–2013. The whole genome sequences (WGS) of BJ04 and BJ09 were generated and compared to other 35 HAdV-C WGSs publicly available. Phylogenetic analyses showed that the BJ04 strain might be the result of three homologous recombination events involving the parental strains JX173086 (HAdV-1), NC_001405 (HAdV-2) and LC068718 (HAdV-6), whereas BJ09 viral genome might be made of genetic elements from JX173083 (HAdV-1), KF268199 (HAdV-5), and KR699642 (strain CBJ113). Despite intratypic recombination, amino acid analysis showed that the gene repertoire of BJ04 and BJ09 were similar to type 2 viruses. Finally, this analysis revealed that at least three lineages of HAdV-C have been identified in China, represented by BJ04 related to NC_001405, BJ09 related to CBJ113, and KF951595 (strain DD28) related to virus isolated in Japan. This study showed that the frequent recombination played an important driving force for complexity of the HAdV-C epidemic in Beijing, thereby demonstrating the necessity for epidemiological and virological surveillance for HAdV-C in China.

## Introduction

Human mastadenovirus (HAdV) is a non-enveloped, double-stranded DNA virus of the family *Adenoviridae* within the genus *Mastadenovirus* ^1^. HAdV genomes generally range from 26 to 45 kb in length^2^. The viral capsid is composed of two types of capsomeres: the hexon and the penton (which consists of the penton base and the fiber)^1^. Hexon and fiber are involved in neutralization as well as hemagglutination inhibition for the later^3^. HAdVs can be divided into 7 species (HAdV-A to G) with more than 70 types based on biological properties, a serum neutralization assay, and whole-genome sequencing^4^. Recently, in order to serve to the adenovirus research community, HAdV working group, being a collaboration between adenoviral researcher and the National Center for Biotechnology Information (NIH)/GenBank, was established with the goals of coordinating and standardizing the process of assigning names to candidate novel HAdV. The working group established a parallel nomenclature based on penton base, hexon and fiber sequences (PHF), and is continuously updating the nomenclature based on biological and genomics data (http://hadvwg.gmu.edu). As multiple studies revealed that HAdV was prone to intratypic recombination, the 9th International Adenovirus Meeting proposed to use whole genome sequences (WGSs) to characterize and name novel HAdV types^5^.

Due to specific and separate tissue tropisms, HAdVs could cause variety of clinical diseases including epidemic keratoconjunctivitis (EKC)^6^, infantile gastroenteritis^7^, fulminant pneumonia, hepatitis and even encephalitis^1^. In addition, HAdV is one of the most common causes of viral acute respiratory infections, which are linked to 5–10% of respiratory illnesses in children^8–10^. Viruses in HAdV-C species (HAdV-C1, C2, C5, C6 and C57) are generally prevalent and commonly associated with respiratory tract infections among pediatric patients^11^. Since HAdV-C viruses have the ability to establish persistent infections, patients can remain asymptomatic carriers until at least their young adulthood^12^.

In 2009, the HAdV-C strain CBJ113 (KR699642) was isolated in Beijing from a hospitalized pediatric patient with severe acute respiratory infection (ARI). Comparative genomic analysis showed that the CBJ113 genome was the result of a recombination between type 1 and type 2 viruses^13^. Another full genome sequence of HAdV-C virus (strain DD28, KF951595) collected in China was reported in GenBank (GB) in 2013 but was not published. Additionally, a surveillance project on the viral etiology of ARI, performed in Beijing in late 2012, identified two HAdV-C strains (BJ04 and BJ09) that were also characterized by a recombination between type 1 and type 2 sequences^14^. In order to understand the relationship between all these HAdV-C strains collected in China, WGSs of strain BJ04 and BJ09 were generated and compared to other HAdV-C strains publicly available. In addition to describing the recombination events characterizing these viruses, this study assessed the consequences of such recombination on the gene repertoire and revealed that multiple lineages of mastadenovirus species C have been identified in Beijing, China.

## Results

### Genomic characterization of BJ04 and BJ09 strains

The genome lengths of BJ04 and BJ09 strains (35,953 and 35,958 bp, respectively) were similar to the length of the reference strain, NC_001405 (HAdV-2, 35,937 bp). The GC content of the three genomes was 55.2% which is a hallmark of HAdV-C genomes^1^. Like NC_001405, BJ04 and BJ09 genomes encoded 37 ORFs which were all conserved in size except for the membrane glycoprotein E3 CR1-beta (nt 29468–29773 in NC_001405). This ORF is predicted to be encoded by 101 amino acids in BJ04 and NC_001405 genomes, whereas a mutation of the start codon resulted in a shorter ORF of 61 amino acids in BJ09 genome. Such a mutation was also observed in other HAdV-C genomes. This ORF is considered to be a relic based on GB annotation. The nucleotide sequences of BJ04 and BJ09 strains were 99.0% identical with 349 nucleotide differences. Pairwise comparisons with the five HAdV-C prototypes (1, 2, 5, 6 and 57) showed that BJ04 and BJ09 strains had the highest identities to HAdV-C2, with 99.2% and 98.9% respectively (Supplementary Table S1).

The average percentage of pairwise identity between the 37 HAdV-C WGSs is 96.7% with the lowest identity at 94.5% (Supplementary Fig. S1). The highest identity between BJ04 and BJ09 and other sequences was about 99.5% (Table 1). Compared to two previously reported Chinese strains CBJ113 and DD28, BJ04 and BJ09 is the most closely related to CBJ113, with 99.1% and 99.5% identity respectively (Supplementary Fig. S1).

**Table 1:**
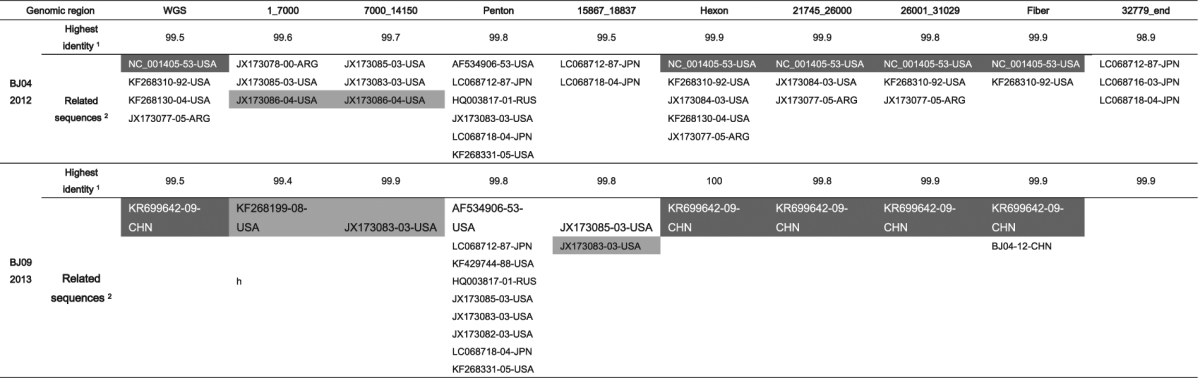
Highest identity to BJ04 and BJ09 among the 35 GB sequences. ^1^Identity percentage were computed from p-distances displayed in Supplementary Figure 2. ^2^Backbone sequences are shaded in dark grey. Additional recombinant partners are shaded in light grey.

### Phylogenetic analysis

**Figure 1:**
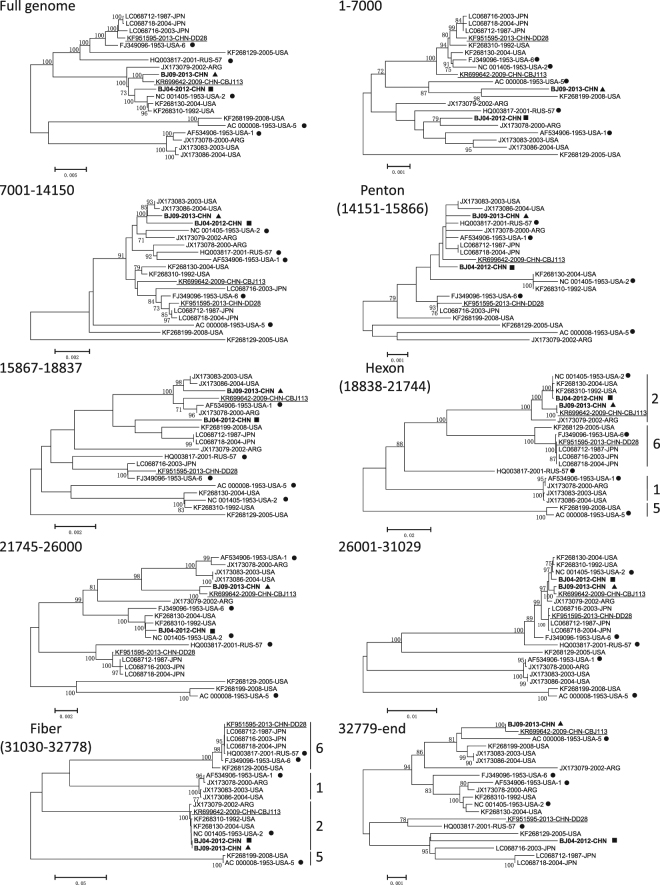
Neighbor joining phylogenetic trees on 20 HAdV-C WGS. The genomic region used to generate the trees is indicated for each tree. Sequences of BJ04 and BJ09 viruses are in bold font. BJ04 sequences are indicated with a black square whereas BJ09 sequences are indicated with a black triangle. The Chinese sequences KR699642 (CBJ113) and KF951595 (DD28) are underlined. Each prototype sequences are indicated by a black dot. Only bootstrap values greater than 70% are indicated. Types designation for the hexon and fiber genes are indicated by brackets. Sequences are indicated by their GB ID followed by the collection year and the country of collection. The type is also indicated for the 5 prototype sequences. Finally, the name of Chinese strain CBJ113 and DD28 is also indicated.

Comprehensive phylogenomic analyses of HAdV-C WGSs were performed with two Beijing strains (BJ04 and BJ09) and other 35 available HAdV-C viruses (Supplementary Fig. S2). The full genome tree constructed by the ML method with 37 WGSs dataset was highly similar to the tree produced by NJ method with the smaller 20 WGSs dataset (Fig. 1). The nodes supported by a posterior probability of 1 in the ML trees were supported by a bootstrap value of 100% in the NJ trees. The phylogenetic NJ tree with 20 WGS featured most of the nodes with 100% bootstrap value (Fig. 1). The larger ML tree with 37 WGS showed a similar picture with most of the nodes having a posterior probability of 1 (Supplementary Fig. S2). This indicated that most of the sequences were highly divergent to each other and that most of them clustered always at the same position in the permuted trees. A similar situation was observed for all genomic regions though with a lower bootstrap value (still greater than 70%) with the exception of the penton tree where only 4 nodes featured a significant bootstrap value (>90%). This demonstrated that most of the penton sequences were very close to each other. Indeed, among the 1725 nucleotides of the 37 analyzed sequences, only 110 sites (6.3%) were divergent and half of these single nucleotide polymorphisms (SNP) were only seen once and therefore not likely to be useful for bootstrap testing (Supplementary Table S2).

Among the four Chinese strains, DD28 appeared to be very different from the others, and closely related to viruses collected in Japan (Fig. 1). BJ04 and BJ09 sequences were highly divergent from each other. BJ09 sequence clustered with the Chinese strain CBJ113, while BJ04 was closely related to NC_001405 (Fig. 1). A similar clustering was only observed in 3 trees based on the hexon gene (nt 18838–21744) and the genomic region upstream of the fiber gene (nt 21745–26000 and nt 26001–31029). The remaining trees had BJ04 and BJ09 clustering to different sequences indicating the existence of potential recombination events.

### Recombination analysis of strain BJ04

BJ04 branch featured a significant bootstrap value in all the trees except for the penton, 15867–18837 and the 32779-end trees (Fig. 1). As mentioned previously, the penton tree was not really informative as the 37 sequences did not feature much divergence between each other. The lack of significant bootstrap in the 15867–18837 and 32779-end trees was confirmed by the relatively low pairwise identity percentages, 99.5% and 98.9% respectively (Table 1). This showed that BJ04 was divergent from known sequences in these two particular genomic regions. As the full genome tree had shown, BJ04 was related to NC_001405 which could be considered as the backbone of the BJ04 genome (Fig. 1). However, BJ04 sequence was showing some divergence at the 5′ and 3′ end of the genome. BJ04 was more related to JX173086 (HAdV-1) on the 5′ end of the genome. The 3′ end of the BJ04 genome was more divergent than the rest of the genome, 98.9% identity with LC068718 (HAdV-6) (Table 1).

**Figure 2:**
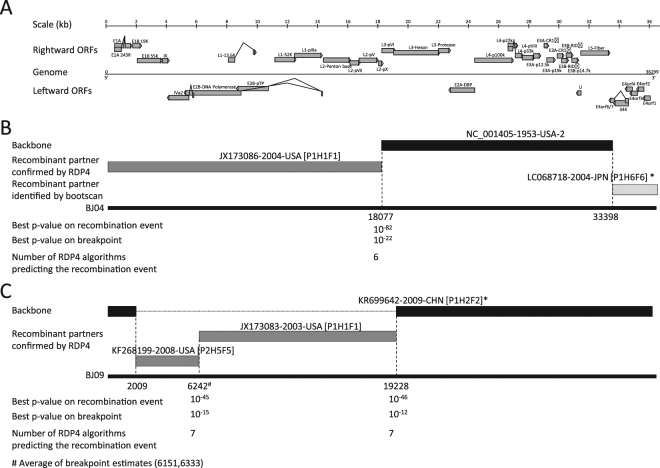
Schematic representation of recombination events within BJ04 and BJ09 genomes. (**A**) Genomic map of HAdV-C. The l-strand of the genome is represented by a straight line. Rightward (top) and leftward (bottom) ORFs are represented by grey arrows based on NC_001405 annotation. (**B**) Recombination events predicted in BJ04. BJ04 genome is shown as a thick black line. The likely backbone and the other genetic components were identified based on Table 1. The likely backbone is shown as a black block. Genetic components predicted by RDP4 to be involved in a recombination event are shown as dark grey blocks whereas genetic components identified only in the bootscan analysis are shown as light grey blocks. Likely breakpoint positions are shown below the genome. The lowest (best) p-values for the prediction of recombination as well as breakpoint are indicated. The number of algorithms of the RDP4 package that were predicting the recombination event is also shown. PHF designations based on our study are identified by an asterisk. (**C**) Recombination events predicted in BJ09. Representation layout similar to (**B**).

RDP4 package strongly confirmed that the strain BJ04 was a highly probable homologous recombinant resulting from NC_001405 and JX173086 with a breakpoint located around 18077, within the gene coding for the capsid protein precursor pVI (Fig. 2, Supplementary Fig. S3). Indeed, 6 algorithms supported this event with p-values ranging from 10^−82^ to 10^−7^. The bootscan output using SimPlot further confirmed this recombination event. In addition, the bootscan algorithm identified one additional recombination event at the 3′ end of the genome between NC_001405 and LC068718. However, the 3′ end of BJ04 genome was very divergent compared to the other sequences. It is a likely that a recombination event occurred but the origin might be unknown.

The amino acid analysis of BJ04 gene repertoire showed that despite detection of multiple recombination events, up to 93% of amino acid residues were conserved in all prototype viruses (Table 2). Among the residues that were not conserved in all prototype viruses, 94% were of type 2 strongly suggesting that BJ04 could be considered as a HAdV-C2 virus despite the recombination event with JX173086 and LC068718 (Table 2, Supplementary Table S3). Finally, 3% of BJ04 residues (25) were not found in any prototype HAdV-Cs. These 25 positions were first visually checked and then compared to the 30 remaining GB sequences. Sixteen of these positions were found only in BJ04 (Table 3). A PROVEAN analysis, which consists mainly on a comparison with orthologs, predicted only one amino acid change (R274C in DNA polymerase) to be deleterious to the protein function. This site is 8 amino acids upstream from the DXE motif of the exonuclease region of the DNA polymerase (Supplementary Fig. S4).

**Table 2:** Comparison of 37 BJ04 amino acid (AA) sequences with homologous sequences encoded by the 5 prototype viruses.

BJ04 amino acid analysis^a^	Number^b,c^	Percentage (%)^b^
Overall number of amino acids (37 ORFs)	11540	/
BJ04 residues conserved in all prototype sequences	10696	92.7
BJ04 residues specific to prototype type 2 sequence	147	17.4
Type 2 residues found in another prototype sequence	643	76.2
BJ04 residues found in other prototype sequence (not type 2)	29	3.4
BJ04 residues not found in any prototype sequences	25	3

Note: ^a^GB annotations were harmonized based on NC_001405 type 2 annotation.

^b^The number and percentage of BJ04 amino acid residues that are not conserved in all prototype sequences are indicated. As BJ04 is highly related to type 2 prototype, the comparison was done in reference to type 2 sequences.

^c^Supplementary Table 5 was used to generate the numbers.

**Table 3:** List of 25 BJ04 amino acid (AA) residues that were not found in any prototype sequences.

ORF Name	Length(AA)	AA change	Genomic position^a^	Other 30 GB sequences^b^	PROVEAN analysis^c^
Control protein E1A	289	A29T	643	2	ND^d^
Control protein E1B 55 K	495	T51A	2157	5	ND
DNA polymerase	1198	V89I	8519	0	Neutral
		N254H	8024	0	Neutral
		R274C	7964	0	Deleterious
Terminal protein precursor pTP	671	A64T	10399	0	Neutral
		A283T	9742	0	Neutral
		D432N	9313	1	ND
		A527T	9028	1	ND
Encapsidation protein 52 K	415	P74S	11259	0	Neutral
Capsid protein precursor pIIIa	585	L474V	13727	0	Neutral
Penton base	571	A368T	15266	3	ND
Single-stranded DNA-binding protein	529	R67C	23881	0	Neutral
Hexon assembly protein 100 K	805	A122T	24471	0	Neutral
Encapsidation protein 22 K	195	S75N	26462	0	Neutral
Protein 33 K	228	S75N	26462	0	Neutral
Control protein E3 12.5 K	107	T50I	28047	0	Neutral
Membrane glycoprotein E3 gp19K	159	K62R	28995	0	Neutral
Fiber	582	S178T	31561	5	ND
Control protein E4orf6/7	150	P66S	33171	10	ND
		Q69R	33161	9	ND
Control protein E4 34 K	294	Y61F	33896	5	ND
		L90I	33810	0	Neutral
Control protein E4orf4	114	H38R	34230	0	Neutral
Control protein E4orf3	116	V70I	34499	0	Neutral

Note: ^a^Genomic positions as well as amino changes for each ORF were indicated.

^b^The number of sequences within the remaining 30 GB sequences featuring the same amino change is shown.

^c^The PROVEAN prediction on the effect of amino changes only found in BJ04 is indicated.

^d^Not done (ND).

### Recombination analysis of strain BJ09

All branches corresponding to the BJ09 sequence were supported by significant bootstrap values except for the penton and the downstream genomic region (15867–18837) (Fig. 1). However, the identity table showed that BJ09 was highly related to known sequences with lowest identity score at 99.4% (Table 1). Most of the trees showed tight relationship with CBJ113 except at the 5′ end of the genome where BJ09 was more related to KF268199 (HAdV-5) and JX173083 (HAdV-1). Therefore, CBJ113 could be considered as the backbone of BJ09 whereas KF268199 and JX173083 are potential genetic components.

Bootscan and RDP4 confirmed 2–3 recombination events between these genomes (Fig. 2). A strong signal was detected by 6 algorithms in RDP4 between CBJ113 and JX173083, with p-values ranging from 10^−46^ to 10^−6^ (Supplementary Fig. S3 online). The breakpoint is likely located around 19228, within the hexon gene. A second recombination event was confirmed by RDP4 involving KF268199 with similar strength. The breakpoint is likely located around 6242, within the DNA polymerase gene. Bootscan predicted an additional breakpoint between CBJ113 and KF268199, around 2 kb from the 5’end of the genome. This event might not be real as recombination event at genome ends are very rare.

The amino acid analysis comparing BJ09 to prototype sequences and CBJ113 showed that 92% of the residues were conserved (Table 4). Among the divergent amino acid residues, only 4 were CBJ113 specific showing that despite a close relationship with CBJ113, BJ09 was not very divergent with the other prototypes. As BJ04 genome, BJ09 genome featured many type 2 specific residues (141) and could be therefore considered as HAdV-C2 virus. Forty-eight of these residues were from the hexon protein (Supplementary Table S4 and Supplementary Table S5). It is worth noticing that the hexon nucleotide tree clearly showed a divergence between BJ04 and BJ09 (Fig. 1). The hexon nucleotide tree showed a clear clustering between all 5 types with long branches and 100% bootstrap at major nodes. The lowest between group mean distances was 9.3. However, the within group distance in type 2 (1.3) was much higher than the distances within the other groups (0.2 to 0.4). This is due to the presence of several sequences in type 2 group that are divergent to the other type 2 sequences. Such divergence was however not observed at the amino acid level with within group distances less than 0.2 (Supplementary Fig. S5). The fiber tree did not show such divergence. BJ04 and BJ069 fiber nucleotide were almost identical. Similarly, the divergence within group was minimal. Fiber type groups are more homogeneous, with within group distance 0.1 to 0.6. Fifteen residues were not found in the other prototype sequences (Table 5). After visually checking these positions, they were compared to the remaining 29 GB sequences, 12 were unique to BJ09 but none were identified as deleterious to protein function by PROVEAN.

**Table 4:** Comparison of 37 BJ09 amino acid (AA) sequences with homologous sequences encoded by the 5 prototype viruses as well as the Chinese CBJ113 strain.

BJ09 amino acid analysis^a^	Number^b,c^	Percentage (%)^b^
Overall number of amino acids (37 ORFs)	11502	/
BJ09 residues conserved in all prototype sequences and CBJ113	10675	92.3
BJ09 residues specific to CBJ113	6	0.7
CBJ113 residues found in any prototype sequences	775	93.9
BJ09 residues found in any prototype sequences (not in CBJ113)	30	3.6
BJ09 residues not found in any prototype sequences nor CBJ113	15	1.8
BJ09 residues specific to type 1	6	/
BJ09 residues specific to type 2	143	/
BJ09 residues specific to type 5	10	/
BJ09 residues specific to type 6	0	/
BJ09 residues specific to type 57	1	/

Note: ^a^GB annotations were harmonized based on NC_001405 type 2 annotation.

^b^The number and percentage of BJ09 amino acid residues that are not conserved in all prototype sequences and in CBJ113 are indicated. As BJ09 is highly related to CBJ113, the comparison was done in reference to CBJ113 sequences. The number of BJ09 residues that are specific to any of the 5 types is shown.

^c^Supplementary Table 6 was used to generate the numbers.

**Table 5:** List of 15 BJ09 amino acid (AA) residues that were not found in any prototype sequences nor CBJ113 sequence.

ORF Name	Length(AA)	AA change	Genomic position^a^	Other 29 GB sequences^b^	PROVEAN analysis^c^
Control protein E1A	289	H94Y	838	0	Neutral
Control protein E1B 55 K	495	A116S	2346	1	ND^d^
		R130K	2389	1	ND
DNA polymerase	1198	S613N	6946	0	Neutral
		P1005H	5770	0	Neutral
Terminal protein precursor pTP	671	P397_E398insEEEEE	9397	0	Neutral
Penton base	571	P23S	14217	0	Neutral
Core protein V	369	A250T	17286	0	Neutral
Protein 33 K	228	S59L	26414	0	Neutral
Encapsidation protein 22 K	195	S59L	26414	0	Neutral
		V134I	26638	0	Neutral

Note: ^a^Genomic positions as well as amino changes for each ORF were indicated.

^b^The number of sequences within the remaining 29 GB sequences featuring the same amino change is shown.

^c^The PROVEAN prediction on the effect of amino changes only found in BJ09 is indicated.

^d^Not done (ND).

## Discussion

This study revealed that at least three distinct lineages of HAdV-C have been identified in China with various geographic spread. BJ09 strain together with CBJ113, which was collected in Beijing in 2009, belongs to a lineage considered as domestic and indicates a continuous circulation of BJ09-like strains in China. BJ04 strain, which is related to viruses close to NC_001405, collected in the USA in 1953, might belong to an international lineage. Finally, the Chinese DD28 strain which was reported in Liaoning province in 2013 but not published and is related to Japanese viruses according to our analysis, could belong to a lineage identified as regional. Therefore, these HAdV-C viruses may have been recently co-circulating in China and could pose a new challenge with regards to acute respiratory diseases in children.

The dataset used for this analysis present various limitations. First, the number of analyzed sequences is limited. We chose to use complete (or nearly complete) genome sequences for the analysis. There were 39 WGSs available in GB and we chose to discard 4 because of large sequence gaps or potential frameshift in some ORFs which might be the sign of poor sequence quality. Among these 35 WGSs, 28 (80%) sequences were generated by two sequencing consortium, one from the USA sequencing 17 American (14 North and 3 South) and 2 Egyptian viruses and one from Japan generating 9 sequences. There is therefore a large bias in terms of collection location in the dataset. For example, viruses from Europe are missing and African viruses are poorly represented. In addition to the location bias, the publicly available sequences were not fully characterized (no publication) and therefore difficult to use unless a full recombination analysis of these sequences is realized which was beyond the scope of this paper. For example, this study clearly shows that viruses collected on the American continent have been circulating in China. It would not be surprising if viruses collected in China have been circulating in the American continent. WGSs from Chinese viruses have been available since 2014 but they have not been used to analyze data from the Americas. It would benefit the entire HAdV-C scientific community if more viruses, from a various collection location like Africa or Europe, were fully sequenced and analyzed. It is good to generate full genome sequence but it would be more useful to fully characterize these sequences and identify potential genetic partners.

Phylogenetic analyses across the genomes of BJ04 and BJ09 indicate that recombination events might be involved in their evolutionary history. BJ04 strain involved three probable homologous recombination events resulting from parent strain JX173086, NC_001405 and LC068718, whereas BJ09 is made of genetic elements from JX173083, KF268199, and CBJ113. Our findings strongly suggested that natural recombination is common in species HAdV-C which might be an essential feature for viral evolution and immune escape. Interestingly, the breaking points for the recombination events within BJ04 and BJ09 are located in the same genomic region (18–19 kb). Further analysis would be necessary to test whether or not this genomic region is a hotspot for recombination.

Recently, newly identified HAdV appeared to originate by recombination among more than 2 viruses within the species^15^. However, such recombination usually requires frequent co-infection of different HAdV types within the same species^16,17^. The species C HAdV can establish long-term latent infections characterized by persistent intermittent excretion in nasopharyngeal secretions, and also in feces excretion for months, or even several years, which may explain why all the types within HAdV-C are so similar among the genomes^18^. The studies have showed that prior infection with one type has no effect on establishment of infection with another type, thus is conducive to establish persistent infections of multiple HAdV-C types after sequential infections early in life^19,20^. Frequent co-infection may facilitate the occurrence of natural recombination within species C HAdV.

Currently, the typing of HAdV is based on penton, hexon and fiber genes. However, as demonstrated in this analysis, the penton sequence is not informative. Hexon and fiber are informative but these two sequences are not enough to identify viruses. For example, BJ04 and BJ09 are sharing hexon and fiber genes but they are clearly coming from a different background. As NGS technology is becoming available, it would be beneficial to the community to develop a surveillance program using WGSs. It is clear that the HAdV community is struggling with nomenclature issues, not only for the viruses but also the gene repertoire. For the viruses, the current nomenclature seems to be based on biological properties, like neutralization and hemagglutination inhibition, ie hexon and fiber. Such nomenclature is ignoring genetic information. We would suggest an additional nomenclature based on lineages. It is for example clear that CBJ113 and BJ09 are related and different from BJ04 even though they all are designated as type 2 viruses.

Our study clearly shows that CBJ113 which was collected in 2009 in China was still circulating in Beijing in 2013. Because of limited surveillance, it is not possible to tell if CBJ113 has been circulating or if a virus closely related to CBJ113 is currently circulating. Similarly, BJ04 is closely related to NC_001405 (HAdV-2). It is difficult to imagine that a virus collected in 1953 could still be circulating. Despite a limited number of analyzed sequences, it is clear that other viruses, collected also in America, are closely related to NC_001405 and these recently collected viruses could be circulating in China. Improving surveillance would be the key to have a better idea of what viruses are circulating and how fast viruses have been replaced.

Genetic analysis showed that most of amino acid differences between the prototype viruses are in hexon and fiber. This means that a recombination event leading to an exchange in other parts of the genome might be rather inconsequential. We cannot rule out the fact that one amino acid change could have a detrimental effect on the biology of the virus but it is rather unlikely. However, characterizing recombination events allow indirectly to identify viruses that are circulating at a certain location and at a certain time. Recombination means co-infection and therefore co-circulation. If a virus is the result of recombination between virus A and virus B, then it means that the patient was infected by both viruses A and B and that both viruses were in the environment at the same time unless the patient was already infected by one of the viruses. Of course such analysis can be meaningful only if there is enough data from various part of the world. For example, if there has been only data from the US, it would have been very difficult to characterize BJ09 recombination event. Therefore, increase of surveillance in various part of the world would benefit the entire community.

HAdV-C viruses, especially HAdV-1, HAdV-2, and HAdV-5, are the most common etiologic agents of respiratory disease in young children (mainly < 5 years of age) and are frequently detected worldwide including in China^21–23^. It is known that more than 80% of the human population is exposed to species C HAdV during childhood^24,25^. HAdV-1 and HAdV-2 were the dominant HAdV types infecting the lower airways of young children with chronic endobronchial suppuration^23^. In addition, prenatal HAdV-C infection can contribute to the earliest steps in development of childhood leukaemia^26^. HAdV-C can also cause significant morbidity and mortality among immunocompromised individuals including organ transplant recipients^27–29^, as well as in children with immunodeficiency disease^29^. Frequent co-infection may facilitate the occurrence of natural recombination within species C HAdV in China. However, whether the recombination of HAdV-C might increase the virulence of circulating viruses needs to be assessed. Furthermore, the epidemic situation of HAdV-C in China remains obscure due to limited research on these viruses, thereby posing a global challenge with regard to acute respiratory disease in children. Therefore, effective HAdV-C surveillance is extremely necessary and could provide insight into disease control and prevention.

## Methods

### Ethics statement

This study was approved by the second session of the Ethics Review Committee of the National Institute for Viral Disease Control and Prevention in China Center for Disease Control and Prevention (CDC). All methods used in this study were performed in accordance with the relevant guidelines and regulations. Written informed consent for the collection of throat swabs for pathogenic identification was obtained from all participants or legal guardians involved in this study.

### Clinical information of strain BJ04 and BJ09

The cases BJ04 and BJ09 were previously reported^14^. BJ04 and BJ09 viruses were collected at the same hospital in Beijing, 5 months apart. BJ04 was collected in November 2012 whereas BJ09 was collected in March 2013. Both viruses were collected from a toddler, less than 1-year-old. BJ04 and BJ09 patients were clinically diagnosed with bronchitis and upper respiratory tract infection, respectively.

### Virus isolation, DNA extraction, amplification and sequencing

BJ04 and BJ09 strains were isolated from tonsil secretions and underwent three passages in HEp-2 cells to obtain high-titer stocks. The viral DNA was extracted using a QIAamp DNA mini kit (Qiagen, Valencia, CA, USA) following the manufacturer’s instructions. The primer pairs used to amplify complete genome was designed based on the sequences of HAdV-1 (AF534906) and HAdV-2 (NC_001405) (Supplementary Table S6). Eight overlapping PCR fragments covering the entire genome, except for the 5′ and 3′ termini, were amplified by using the Platinum PCR SuperMix (Invitrogen, Carlsbad, CA, USA) according to the manufacturer’s protocol. The PCR products were cleaned up using a QIA Gel Extraction kit (Qiagen, Valencia, CA, USA), and then sequenced using Sanger sequencing method with a BigDye Terminator chemistry (Version 3.1; Life Technologies, NY, USA) and the 3100 Genetic Analyzer (Life Technologies, Japan). 5’/3’ RACE kit (Roche, Indianapolis, IN) was used to obtain the sequences of 5’ and 3’ termini. WGSs was obtained from 66 overlapping sequences (average length of 880 nt) assembled in Sequencher version 5.0 (Genecode). Consensus sequences were annotated in Artemis version 16.00 using NC_001405 as a template^30^. Annotated genome sequences of BJ04 and BJ09 were submitted to GB under accession number MF315028 and MF315029 respectively.

### Datasets

Thirty-nine HAdV-C WGSs were downloaded from GB (Supplementary Table S7). Among them, four sequences (KF268331, KF429744, AY339865, AY601635) were excluded due to large sequence gaps or frameshifts in ORFs. A total of 37 WGSs including the two Beijing strains reported in this study (BJ04 and BJ09) were aligned using MAFFT version 7^31^. Viruses were identified by their GB ID except for the Chinese viruses KF951595 and KR699642 which were identified by their strain name, DD28 and CBJ113, respectively. For convenient display, a smaller dataset of 20 sequences was also used including 2 *de novo* sequences, 5 prototype sequences (type 1, 2, 5, 6 and 57), 2 sequences of virus collected in China CBJ113 and DD28) and 11 other sequences that were genetically related to BJ04 and BJ09 sequences (Supplementary Table S7). Multiple sequence alignments were edited in BioEdit^32^. WGSs alignment was split into 9 pieces in order to monitor potential recombination events within the penton base, hexon and fiber knob genes as well as the rest of the genome. The numbering used in this analysis is based on NC_001405 annotation.

### Phylogenetic analysis

Phylogenetic trees were generated with MEGA6.0 using the neighbor-joining (NJ) method with the maximum composite likelihood nucleotide substitution model and bootstrap test of phylogeny with replicates set to 1000^33–35^. Phylogenetic trees were also generated with phyML version 3.1 using the maximum likelihood (ML) method (Supplementary Fig. S2)^36^. Genetic distances were computed in MEGA6.0 (Supplementary Fig. S1 and Supplementary Table S1).

### Recombination analysis

Potential genomic components were identified based on genetic distances and phylogenetic analyses. Bootscan analysis in the SimPlot package version 3.5.1 and RDP4 suite were used to test potential recombination events^37,38^. Bootscan was run with a window size of 1000 and a step size of 200. RDP4 was run with the following algorithms (RDP, GENECONV, Chimeara, MaxChi, Bootscan, SiScan and 3Seq) using default parameters, Bootscan outputs and RDP4 p-values are shown in Supplementary Figure 3. RDP4 p-values lower than 10^−5^ were considered significant. Breakpoint locations were estimated based on bootscan output when the recombination event was not identified by RDP4 package.

### Amino acid analysis

Genome annotations were harmonized in Artemis using NC_001405 as template. BJ04 and BJ09 ORFs were compared to 5 prototype sequences and the 30 remaining HAdV-C WGSs from GB. Unique amino acid changes in BJ04 and BJ09 were tested with PROVEAN, which scan orthologs for these changes, in order to assess whether these amino acid substitutions were likely to be detrimental for the protein function (http://provean.jcvi.org/seq_submit.php).

## Electronic supplementary material

Supplementary material
