# Supplementary material for: Whole genomic analysis of two potential recombinant strains within Human mastadenovirus species C previously found in Beijing, China
Source: Sci Rep. 2017 Nov 13;7:15380. doi: 10.1038/s41598-017-15336-2 (PMC5684218; doi:10.1038/s41598-017-15336-2)
Supplement: Supplementary file 1 — Supplementary material [file 41598_2017_15336_MOESM1_ESM.pdf]

Whole genomic analysis of two potential recombinant strains within Human mastadenovirus species C previously found in Beijing, China

Naiying Mao[#1], Zhen Zhu[#1], Pierre Rivailler[#1], Meng Chen[2], Qin Fan[1], Fang Huang[2*] and Wenbo Xu[1*]

Supplementary Table S1: Comparisons of nucleotide and amino acid sequences of BJ04 and BJ09 and strains of species C.

Supplementary Table S2: Nucleotide differences within 37 HAdV-C penton base sequences.

Supplementary Table S3 Amino acid analysis of the 37 ORFs encoded by the BJ04 genome.

Supplementary Table S4: Amino acid analysis of the 37 ORFs encoded by the BJ09 genome.

Supplementary Table S5: Amino acid differences between all 5 HAdV-C prototypes, CBJ113, BJ04 and BJ09 within all 37 ORFs.

Supplementary Table S6. Primers used for PCR amplification of the complete genome.

Supplementary Table S7: List of GB sequences of HAdV-C used in the analysis

Supplementary Figure S1: Pairwise p-distances between the 37 HAdV-C sequences for the 10 genomic regions. p-distances were computed in MEGA6. The lowest p-distances

for BJ04 and BJ09 are boxed and were used to generate Table 1. Average distance and maximum distance are shown for the WGSs.

Supplementary Figure S2: Maximum likelihood trees of the 37 HAdV-C sequences for the 10 genomic regions. Posterior probabilities greater than 0.7 are shown. BJ04 sequences are shown in red whereas BJ09 sequences are shown in green. The 5 HAdV-C prototype sequences are indicated with a black dot. The Chinese strain DD28 and CBJ113 are indicated with a black square. The other sequences used to generate Figure 1 (20 sequences dataset) are indicated with a black triangle.

Supplementary Figure S3: Recombination analysis for BJ04 and BJ09 genomes. Bootscan output is shown with the backbone in red and additional genetic partners in green and blue. RDP4 output is shown with the schematic sequence display and a compilation of the recombination information display.

Supplementary Figure S4: PROVEAN alignment of 168 orthologs for the position 274 in the DNA polymerase ORF. Identical sequences were discarded. BJ04 sequence is shown at the top and a consensus sequence with a 75% threshold is shown at the bottom. Amino acids identical to BJ04 sequence are shown with a dot and gaps are shown with a dash. Only the amino acids 260 to 288 of the DNA polymerase ORF are shown. The motif DXE of the ExoI region of the exonuclease domain of DNA polymerase is shown according to Liu et al. 2000.

Supplementary Figure S5: Neighbor joining phylogenetic tree on 37 amino acid sequences of the hexon ORF. Hexon types are shown with brackets. Bootstrap values greater than 70% are shown.

References

1 Chroboczek, J., Bieber, F. & Jacrot, B. The sequence of the genome of adenovirus type 5 and its comparison with the genome of adenovirus type 2. *Virology.* **186**, 280-285. (1992).

2 Hang, J. *et al.* Adenovirus type 4 respiratory infections with a concurrent outbreak of coxsackievirus A21 among United States Army Basic Trainees, a retrospective viral etiology study using next-generation sequencing. *J Med Virol.* **89**, 1387-1394. doi: 1310.1002/jmv.24792. Epub 22017 Mar 24793. (2017).

3 Lauer, K. P. *et al.* Natural variation among human adenoviruses: genome sequence and annotation of human adenovirus serotype 1. *J Gen Virol.* **85**, 2615-2625. (2004).

4 Liu, H., Naismith, J. H. & Hay, R. T. Identification of conserved residues contributing to the activities of adenovirus DNA polymerase. *J Virol.* **74**, 11681-11689. (2000).

5 Roberts,R.J., Akusjaervi,G., Alestroem,P., Gelinas,R.E., Gingeras,T.R., Sciaky,D. and Pettersson,U. A consensus sequence for the adenovirus-2 genome. Doerfler,W. (Ed.); ADENOVIRUS DNA: 1-51; Martinus Nijhoff Publishing, Boston (1986)

6 Walsh, M. P. *et al.* Computational analysis of two species C human adenoviruses provides evidence of a novel virus. *J Clin Microbiol.* **49**, 3482-3490. doi: 3410.1128/JCM.00156-00111. Epub 02011 Aug 00117. (2011).

7 Wang, Y. *et al.* Phylogenetic evidence for intratypic recombinant events in a novel human adenovirus C that causes severe acute respiratory infection in children. *Sci Rep.* **6:23014.**, 10.1038/srep23014. (2016).

8 Weaver, E. A. *et al.* Characterization of species C human adenovirus serotype 6 (Ad6). *Virology.* **412**, 19-27. doi: 10.1016/j.virol.2010.1010.1041. Epub 2011 Jan 1020. (2011).

Supplementary table 1. Comparisons of nucleotide (nt) and amino acid (aa) sequences of BJ04 and BJ09, and strains of Species C

Coding region	% Similarity							
	HAdV-1(AF534906)		HAdV-2(AC_000007)		HAdV-5(AC_000008)		HAdV-6(FJ349096)	
	nt (BJ04/BJ09)	aa (BJ04/BJ09)	nt (BJ04/BJ09)	aa (BJ04/BJ09)	nt (BJ04/BJ09)	aa (BJ04/BJ09)	nt (BJ04/BJ09)	aa (BJ04/BJ09)
E1B 55kDa protein	99.0/97.4	98.8/97.2	98.7/97.9	99.1/97.9	98.1/98.8	97.4/98.9	98.6/98.6	99.1/97.9
pIX	100.0/99.5	100.0/98.5	99.5/100.0	98.5/100.0	99.7/99.7	99.2/99.2	99.5/100.0	98.5/100.0
pIVa2	99.7/99.1	100.0/100.0	98.9/99.2	99.5/99.5	99.0/99.3	99.5/99.5	98.9/99.2	99.5/99.5
DNA polymerase	99.1/99.1	99.3/99.4	98.8/98.9	99.1/99.2	98.8/98.8	99.0/99.1	98.9/98.9	99.2/99.3
pTP	99.2/98.5	99.2/99.1	99.0/98.7	98.8/98.9	99.1/98.2	99.2/98.8	99.5/98.6	99.4/98.9
L1 52.5kDa protein	99.2/99.4	99.7/100.0	99.7/99.9	99.7/100.0	98.4/98.6	99.0/99.2	99.5/99.7	99.5/99.7
L1 pIIIa	99.3/99.6	99.8/100.0	99.4/99.7	99.8/99.8	98.8/99.1	99.8/100.0	99.0/99.3	99.6/99.8
L2 penton base	99.7/99.8	99.8/99.8	98.7/98.6	98.7/98.7	98.0/97.9	98.0/98.0	99.5/99.5	99.6/99.6
L2 pVII	99.1/99.3	100.0/100.0	98.3/98.4	100.0/100.0	98.6/98.8	100.0/100.0	99.4/99.6	100.0/100.0
L2 pV	99.8/99.4	99.7/99.4	98.3/98.1	99.1/98.9	98.0/97.6	99.1/98.9	98.8/98.5	99.7/99.4
L3 pVI	97.7/100.0	98.4/100.0	100.0/97.7	100.0/98.4	98.4/97.4	98.8/98.8	98.1/97.0	98.8/98.8
L3 hexon	85.2/85.6	90.9/90.9	99.9/98.8	100.0/100.0	82.0/81.5	86.5/86.5	88.7/88.8	84.1/84.1
E2A DBP	97.0/98.9	97.7/99.4	99.8/97.4	99.8/98.1	97.4/96.9	98.1/98.2	99.6/97.2	99.6/98.2
L4 100kDa protein	98.1/98.3	97.8/98.3	99.7/99.1	99.7/99.0	96.8/96.9	97.5/97.7	99.4/98.9	99.6/98.8
L4 pVIII	98.5/98.3	100.0/100.0	100.0/99.8	100.0/100.0	96.3/96.1	99.1/99.1	99.8/99.7	100.0/100.0
L5 fiber	72.1/72.1	72.5/72.5	99.9/99.8	99.8/99.8	72.4/72.4	69.1/69.1	68.0/68.0	67.6/67.6
E4 34kDa protein	97.5/99.3	98.6/100.0	97.5/99.3	98.6/100.0	97.5/99.7	98.6/100.0	97.5/99.0	98.6/100.0
WGS	94.3/94.5	-	99.2/98.9	-	93.7/93.8	-	96.6/96.4	-

Note: The maximum similarities of nucleotide (nt) and amino acid (aa) sequences of BJ04 were indicated by red and pink color, respectively; the maximum similarities of nucleotide (nt) and amino acid (aa) sequences of BJ09 were indicated by blue and green color, respectively.

Supplementary Table S2: Nucleotide differences within 37 HAdV -C penton base sequences

	P[1]	5[2,3]	6	51	67	90	96	120	147	192	213	274	276	348	363	375	441	444	455	458
KF268310.1	2	a	g	c	c	c	c	t	c	c	t	c	a	t	c	c	g	g	c	t
JX173077.1	2	a	g	c	c	c	c	t	c	c	t	c	a	t	c	c	g	g	c	t
KF268130.1	2	a	g	c	c	c	c	t	c	c	t	c	a	t	c	c	g	g	c	t
NC 001405	2	a	g	c	c	c	c	t	c	c	t	c	a	t	c	c	g	g	c	t
JX173084.1	2	a	g	c	c	c	c	t	c	c	t	c	a	t	c	c	g	g	c	t
KF429754.1	5	g	g	t	c	t	c	t	c	c	g	c	g	g	c	c	g	g	t	c
KF268127.1	5	g	g	t	c	t	c	t	c	c	g	c	g	g	c	c	g	g	t	c
M73260.1	5	g	g	t	c	t	c	t	c	c	g	c	g	g	c	c	g	g	t	c
KF951595.1	6	g	g	c	c	c	c	c	c	c	t	c	a	g	c	c	g	a	c	t
LC068713.1	6	g	g	c	c	c	c	c	c	c	t	c	a	g	c	c	g	g	c	t
LC068715.1	6	g	g	c	c	c	c	c	c	c	t	c	a	g	c	c	g	g	c	t
LC068714.1	6	g	g	c	c	c	c	c	c	c	t	c	a	g	c	c	g	g	c	t
LC068716.1	6	g	g	c	c	c	c	c	c	c	t	c	a	g	c	c	g	g	c	t
JX423389.1	6	g	g	c	c	c	c	c	c	c	t	c	a	g	c	c	g	g	c	t
HQ413315.1	6	g	g	c	c	a	c	c	c	c	t	c	a	g	c	c	g	g	c	t
FJ349096.1	6	g	g	c	c	a	c	c	c	c	t	c	a	g	c	c	g	g	c	t
HQ003817.1	1	g	g	c	c	c	c	c	c	c	t	c	a	g	c	c	g	g	c	t
KR699642.1	1	g	g	c	c	c	c	c	c	c	t	c	a	t	c	c	g	g	c	t
BJ09-2013-2	1	g	g	c	t	c	c	c	c	c	t	c	a	g	c	c	g	g	c	t
LC068720.1	1	g	g	c	c	c	c	c	c	c	t	c	a	g	c	c	g	g	c	t
LC068719.1	1	g	g	c	c	c	c	c	c	c	t	c	a	g	c	c	g	g	c	t
LC068717.1	1	g	g	c	c	c	c	c	c	c	t	c	a	g	t	c	g	g	c	t
LC068712.1	1	g	g	c	c	c	c	c	c	c	t	c	a	g	c	c	g	g	c	t
LC068718.1	1	g	g	c	c	c	c	c	c	c	t	c	a	g	c	c	g	g	c	t
BJ04-2012-2	1	g	g	c	c	c	c	c	c	c	t	c	a	g	c	c	g	g	c	t
JX173086.1	1	g	g	c	c	c	t	c	c	c	t	c	a	g	c	c	g	g	c	t
JX173085.1	1	g	g	c	c	c	c	c	c	c	t	c	a	g	c	c	g	g	c	t
JX173083.1	1	g	g	c	c	c	c	c	c	c	t	c	a	g	c	c	g	g	c	t
AF534906.1	1	g	g	c	c	c	c	c	c	c	t	c	a	g	c	c	g	g	c	t
JX173082.1	1	g	g	c	c	c	c	c	c	c	t	c	a	g	c	c	g	g	c	t
JX173078.1	1	g	g	c	c	c	c	c	c	c	t	c	a	g	c	c	g	g	c	t
JX173080.1	1	g	g	c	c	c	c	c	c	t	t	c	g	g	c	c	g	g	c	t
KF268129.1		g	g	c	c	t	c	c	c	c	g	t	g	g	c	c	g	g	c	t
KF268199.1		g	g	c	c	c	c	c	c	c	t	c	g	g	c	c	g	g	c	t
JX173079.1		g	g	c	c	c	c	c	c	c	t	c	g	t	c	c	g	g	c	a
KX384959.1		g	g	c	c	c	c	c	c	c	t	c	g	t	c	c	g	g	c	a
JX173081.1		g	a	c	c	t	c	t	t	c	g	t	g	g	c	a	a	g	c	-

1: P type is indicated.
2: Nucleotide position within the penton gene.
3: SNPs are indicated in green.

Supplementary Ta

	P[1]	459	460	461	462	465	471	477	489	492	513	519	528	534	549	561	564	576	585
KF268310.1	2	t	a	c	t	g	a	g	t	g	c	c	c	c	t	g	c	c	g
JX173077.1	2	t	a	c	t	g	a	g	t	g	c	c	c	c	t	g	c	c	g
KF268130.1	2	t	a	c	t	g	a	g	t	g	c	c	c	c	t	g	c	c	g
NC 001405	2	t	a	c	t	g	a	g	t	g	c	c	c	c	t	g	c	c	g
JX173084.1	2	t	a	c	t	g	a	g	t	g	c	c	c	c	t	g	c	c	g
KF429754.1	5	t	a	c	t	g	t	g	c	g	c	c	c	c	t	g	c	c	g
KF268127.1	5	t	a	c	t	g	t	g	c	g	c	c	c	c	t	g	c	c	g
M73260.1	5	t	a	c	t	g	t	g	c	g	c	c	c	c	t	g	c	c	g
KF951595.1	6	t	a	c	t	g	a	g	c	g	c	c	c	c	t	g	c	c	g
LC068713.1	6	t	a	c	t	g	a	g	c	g	c	c	c	c	t	g	c	c	g
LC068715.1	6	t	a	c	t	g	a	g	c	g	c	c	c	c	t	g	c	c	g
LC068714.1	6	t	a	c	t	g	a	g	c	g	c	c	c	c	t	g	c	c	g
LC068716.1	6	t	a	c	t	g	a	g	c	g	c	c	c	c	t	g	c	c	g
JX423389.1	6	t	a	c	t	g	a	g	c	g	c	c	c	c	t	g	c	c	g
HQ413315.1	6	t	a	c	t	g	a	g	c	g	c	c	c	c	t	g	c	c	g
FJ349096.1	6	t	a	c	t	g	a	g	c	g	c	c	c	c	t	g	c	c	g
HQ003817.1	1	t	a	c	t	g	a	g	t	g	c	c	c	c	t	g	c	c	g
KR699642.1	1	t	a	c	t	g	a	g	t	g	c	c	c	c	t	g	c	c	g
BJ09-2013-2	1	t	a	c	t	g	a	g	t	g	c	c	c	c	t	g	c	c	g
LC068720.1	1	t	a	c	t	g	a	g	t	g	c	c	c	c	t	g	c	c	g
LC068719.1	1	t	a	c	t	g	a	g	t	g	c	c	c	c	t	g	c	c	g
LC068717.1	1	t	a	c	t	g	a	g	c	g	c	c	c	c	t	g	c	c	g
LC068712.1	1	t	a	c	t	g	a	g	t	g	c	c	c	c	t	g	c	c	g
LC068718.1	1	t	a	c	t	g	a	g	t	g	c	c	c	c	t	g	c	c	g
BJ04-2012-2	1	t	a	c	t	g	a	g	t	g	c	c	c	c	t	g	c	c	g
JX173086.1	1	t	a	c	t	g	a	g	t	g	c	c	c	c	t	g	c	c	g
JX173085.1	1	t	a	c	t	g	a	g	t	g	c	c	c	c	t	g	c	c	g
JX173083.1	1	t	a	c	t	g	a	g	t	g	c	c	c	c	t	g	c	c	g
AF534906.1	1	t	a	c	t	g	a	g	t	g	c	c	c	a	t	g	c	c	g
JX173082.1	1	t	a	c	t	g	a	g	t	g	c	c	c	a	t	g	c	c	g
JX173078.1	1	t	a	c	t	g	a	g	t	g	c	c	c	c	t	g	c	c	g
JX173080.1	1	t	a	c	t	g	a	g	t	a	c	c	c	c	t	g	c	c	g
KF268129.1		t	a	c	t	g	t	g	c	g	c	c	c	c	t	t	c	c	g
KF268199.1		t	a	c	t	g	t	g	c	g	c	c	c	c	t	g	c	t	g
JX173079.1		g	a	c	c	a	t	g	c	g	c	a	a	c	g	g	c	c	a
KX384959.1		g	a	c	c	a	t	g	c	g	c	a	a	c	g	g	c	c	a
JX173081.1		-	g	t	t	g	t	a	c	g	a	c	c	c	g	g	t	c	g

1: P type is indicat

2: Nucleotide posit

3: SNPs are indica

Supplementary Ta

	P[1]	591	594	604	618	624	646	648	649	663	672	726	729	765	775	780	783	798	804
KF268310.1	2	g	g	c	c	a	a	a	c	a	t	g	g	g	t	c	c	c	g
JX173077.1	2	g	g	c	c	a	a	a	c	a	t	g	g	g	t	c	c	c	g
KF268130.1	2	g	g	c	c	a	a	a	c	a	t	g	g	g	t	c	c	c	g
NC 001405	2	g	g	c	c	a	a	a	c	a	t	g	g	g	t	c	c	c	g
JX173084.1	2	g	g	c	c	a	a	a	c	a	t	g	g	g	t	c	c	c	g
KF429754.1	5	a	g	c	c	a	a	a	c	c	t	g	g	g	t	c	c	c	g
KF268127.1	5	a	g	c	c	a	a	a	c	c	t	g	g	g	t	c	c	c	g
M73260.1	5	a	g	c	c	a	a	a	c	c	t	g	g	g	t	c	c	c	g
KF951595.1	6	g	g	c	c	a	a	a	t	a	t	g	g	g	t	c	c	c	g
LC068713.1	6	g	g	c	c	a	a	a	c	a	t	g	g	g	t	c	c	c	g
LC068715.1	6	g	g	a	c	a	a	a	c	a	t	g	g	g	t	c	c	c	g
LC068714.1	6	g	g	a	c	a	a	a	c	a	t	g	g	g	t	c	c	c	g
LC068716.1	6	g	g	c	c	a	a	a	c	a	t	g	g	g	t	c	c	c	g
JX423389.1	6	g	g	c	c	a	a	a	c	a	t	g	g	g	t	c	c	c	g
HQ413315.1	6	g	g	c	c	a	a	a	c	a	t	g	g	g	t	c	c	c	g
FJ349096.1	6	g	g	c	c	a	a	a	c	a	t	g	g	g	t	c	c	c	g
HQ003817.1	1	g	g	c	c	a	a	a	c	a	t	g	g	g	t	c	c	c	g
KR699642.1	1	g	g	c	c	a	a	a	c	a	t	g	g	g	t	c	t	c	g
BJ09-2013-2	1	g	g	c	c	a	a	a	c	a	t	g	g	g	t	c	c	c	g
LC068720.1	1	g	g	c	c	a	a	a	c	a	t	g	g	g	t	c	c	c	g
LC068719.1	1	g	g	c	c	a	a	a	c	a	t	g	g	g	t	c	c	c	g
LC068717.1	1	g	g	c	c	a	a	a	c	a	t	g	g	g	t	c	c	c	g
LC068712.1	1	g	g	c	t	a	a	a	c	a	t	g	g	g	t	c	c	c	g
LC068718.1	1	g	g	c	t	a	a	a	c	a	t	g	g	g	t	c	c	c	g
BJ04-2012-2	1	g	g	c	c	a	a	a	c	a	t	g	g	g	t	c	c	c	g
JX173086.1	1	g	g	c	c	a	a	a	c	a	t	g	g	g	t	c	c	c	g
JX173085.1	1	g	g	c	c	a	a	a	c	a	t	g	g	g	t	c	c	c	g
JX173083.1	1	g	g	c	c	a	a	a	c	a	t	g	g	g	t	c	c	c	g
AF534906.1	1	g	g	c	c	a	a	a	c	a	t	g	g	g	t	c	c	c	g
JX173082.1	1	g	g	c	c	a	a	a	c	a	t	g	a	g	t	c	c	c	g
JX173078.1	1	g	g	c	c	a	a	a	c	a	t	g	g	g	t	c	c	t	g
JX173080.1	1	g	g	c	c	a	a	a	c	a	t	a	g	g	t	c	c	c	g
KF268129.1		g	g	c	c	c	a	a	c	c	c	g	g	g	c	c	c	c	g
KF268199.1		g	g	c	c	a	a	a	c	a	t	g	g	g	t	t	c	c	g
JX173079.1		g	a	c	c	a	a	a	c	a	t	g	g	g	t	c	c	c	a
KX384959.1		g	a	c	c	a	a	a	c	a	t	g	g	g	t	c	c	c	a
JX173081.1		g	g	c	t	c	c	g	c	c	t	g	g	c	t	c	c	c	g

1: P type is indicat
2: Nucleotide posit
3: SNPs are indica

Supplementary Ta

	P[1]	816	819	821	831	835	844	882	888	900	909	911	914	926	928	935	948	952	975
KF268310.1	2	g	c	c	c	g	a	a	g	c	c	g	g	g	g	a	c	g	c
JX173077.1	2	g	c	c	c	g	a	a	g	c	c	g	g	g	g	a	c	g	c
KF268130.1	2	g	c	c	c	g	a	a	g	c	c	g	g	g	g	a	c	g	c
NC 001405	2	g	c	c	c	g	a	a	a	c	c	g	a	g	g	a	c	g	c
JX173084.1	2	g	c	c	c	a	a	a	g	c	c	g	g	a	g	a	c	g	c
KF429754.1	5	g	c	c	t	g	a	g	g	c	c	g	g	g	a	g	c	g	c
KF268127.1	5	g	c	c	t	g	a	g	g	c	c	g	g	g	a	g	c	g	c
M73260.1	5	g	c	c	t	g	a	g	g	c	c	g	g	g	a	g	c	g	c
KF951595.1	6	g	c	c	c	g	a	a	g	c	c	g	g	g	g	a	c	g	t
LC068713.1	6	g	c	c	c	g	a	a	g	c	c	g	g	g	g	a	c	g	t
LC068715.1	6	g	c	c	c	g	a	a	g	c	c	g	g	g	g	a	c	g	t
LC068714.1	6	g	c	c	c	g	a	a	g	c	c	g	g	g	g	a	c	g	t
LC068716.1	6	g	c	c	c	g	a	a	g	c	c	g	g	g	g	a	c	g	t
JX423389.1	6	g	c	c	c	g	a	a	g	c	c	g	g	g	g	a	c	g	t
HQ413315.1	6	g	c	c	c	g	a	a	g	c	c	g	g	g	g	a	c	g	t
FJ349096.1	6	g	c	c	c	g	a	a	g	c	c	g	g	g	g	a	c	g	t
HQ003817.1	1	g	c	c	c	g	a	a	g	c	c	g	g	g	g	a	c	g	c
KR699642.1	1	g	c	c	c	g	a	a	g	c	c	g	g	g	g	a	c	g	c
BJ09-2013-2	1	a	c	c	c	g	a	a	g	c	c	g	g	g	g	a	c	g	c
LC068720.1	1	g	c	c	c	g	a	a	g	c	c	g	g	g	g	a	t	g	c
LC068719.1	1	g	c	c	c	g	a	a	g	c	c	g	g	g	g	a	t	g	c
LC068717.1	1	g	c	c	c	g	a	a	g	c	c	g	g	g	g	a	t	g	c
LC068712.1	1	g	c	c	c	g	a	a	g	c	c	g	g	g	g	a	c	g	c
LC068718.1	1	g	c	c	c	g	a	a	g	c	c	g	g	g	g	a	c	g	c
BJ04-2012-2	1	g	c	c	c	g	a	a	g	c	c	g	g	g	g	a	c	g	c
JX173086.1	1	g	c	t	c	g	a	a	g	c	t	g	g	g	g	a	c	g	c
JX173085.1	1	g	c	c	c	g	a	a	g	c	t	g	g	g	g	a	c	g	c
JX173083.1	1	g	c	c	c	g	a	a	g	c	t	g	g	g	g	a	c	g	c
AF534906.1	1	g	c	c	c	g	a	a	g	c	c	g	g	g	g	a	c	g	c
JX173082.1	1	g	c	c	c	g	a	a	g	c	c	g	g	g	g	a	c	g	c
JX173078.1	1	g	c	c	c	g	c	a	g	c	c	g	g	g	g	a	c	g	c
JX173080.1	1	g	c	c	c	g	a	a	g	c	c	g	g	g	g	a	c	g	c
KF268129.1		g	t	c	c	g	a	g	g	t	c	g	g	g	g	g	c	g	c
KF268199.1		g	c	c	c	g	a	a	g	c	c	g	g	a	g	-	c	g	c
JX173079.1		g	c	c	c	g	a	g	g	c	c	g	g	g	g	g	c	g	c
KX384959.1		g	c	c	c	g	a	g	g	c	c	g	g	g	g	g	c	g	c
JX173081.1		g	t	c	c	g	a	a	g	c	c	a	g	g	g	g	c	a	c

1: P type is indicat
2: Nucleotide posit
3: SNPs are indica

Supplementary Ta

	P[1]	996	1044	1047	1053	1059	1066	1068	1077	1079	1080	1083	1095	1102	1107	1116
KF268310.1	2	g	g	g	g	t	g	g	a	a	a	t	t	-	-	g
JX173077.1	2	g	g	g	g	t	g	g	a	a	a	t	t	-	-	g
KF268130.1	2	g	g	g	g	t	g	g	a	a	a	t	t	-	-	g
NC 001405	2	g	g	g	g	t	g	g	a	a	a	t	t	-	-	g
JX173084.1	2	g	g	g	g	t	g	g	c	a	a	t	t	-	-	g
KF429754.1	5	g	t	g	g	t	g	a	c	a	a	t	t	-	-	g
KF268127.1	5	g	t	g	g	t	g	a	c	a	a	t	t	-	-	g
M73260.1	5	g	t	g	g	t	g	a	c	a	a	t	t	-	-	g
KF951595.1	6	g	g	g	g	t	g	g	c	a	a	t	t	g	a	g
LC068713.1	6	g	g	g	g	t	g	g	c	a	a	t	t	g	a	g
LC068715.1	6	g	g	g	g	t	g	g	c	a	a	t	t	g	a	g
LC068714.1	6	g	g	g	g	t	g	g	c	a	a	t	t	g	a	g
LC068716.1	6	g	g	g	g	t	g	g	c	a	a	t	t	g	a	g
JX423389.1	6	g	g	g	g	t	g	g	c	a	a	t	t	g	a	g
HQ413315.1	6	g	g	g	g	t	g	g	c	a	a	t	t	g	a	g
FJ349096.1	6	g	g	g	g	t	g	g	c	a	a	t	t	g	a	g
HQ003817.1	1	t	g	g	g	t	g	g	c	a	a	t	t	g	a	g
KR699642.1	1	g	g	g	g	t	g	g	c	a	a	t	t	g	a	g
BJ09-2013-2	1	g	g	g	g	t	g	g	c	a	a	t	t	g	a	g
LC068720.1	1	g	g	g	g	a	g	g	c	a	a	t	t	a	a	g
LC068719.1	1	g	g	g	g	a	g	g	c	a	a	t	t	a	a	g
LC068717.1	1	g	g	g	g	a	g	g	c	a	a	t	t	a	a	g
LC068712.1	1	g	g	g	g	t	g	g	c	a	a	t	t	g	a	g
LC068718.1	1	g	g	g	g	t	g	g	c	a	a	t	t	g	a	g
BJ04-2012-2	1	g	g	g	g	t	g	g	c	a	a	t	t	a	a	g
JX173086.1	1	g	g	g	g	t	g	g	c	a	a	t	t	g	a	g
JX173085.1	1	g	g	g	g	t	g	g	c	a	a	t	t	g	a	g
JX173083.1	1	g	g	g	g	t	g	g	c	a	a	t	t	g	a	g
AF534906.1	1	g	g	g	g	t	g	g	c	a	a	t	t	g	a	g
JX173082.1	1	g	g	g	g	t	g	g	c	a	a	t	t	g	a	g
JX173078.1	1	g	g	g	g	t	g	g	c	a	a	t	t	g	a	g
JX173080.1	1	g	g	g	g	t	g	g	c	a	a	t	t	g	a	g
KF268129.1		g	g	g	a	t	g	g	c	a	a	t	t	g	g	g
KF268199.1		g	g	g	g	t	g	g	c	a	a	t	t	-	-	g
JX173079.1		g	t	g	g	t	g	a	c	a	a	-	t	g	g	g
KX384959.1		g	t	g	g	t	g	a	c	a	a	-	t	g	g	g
JX173081.1		-	g	a	g	t	a	g	a	c	g	c	c	g	a	t

1: P type is indicat
2: Nucleotide posit
3: SNPs are indica

Supplementary Ta

	P [1]	1118	1122	1134	1146	1156	1170	1194	1196	1258	1260	1263	1266	1269	1281	1320
KF268310.1	2	t	g	g	c	a	g	c	a	a	c	g	c	c	c	c
JX173077.1	2	t	g	g	c	a	g	c	a	a	c	g	c	c	c	c
KF268130.1	2	t	g	g	c	a	g	c	a	a	c	g	c	c	c	c
NC 001405	2	t	g	g	c	a	g	c	a	a	c	g	c	c	c	c
JX173084.1	2	t	a	g	t	a	g	t	a	g	c	g	c	c	g	a
KF429754.1	5	t	g	g	c	a	g	c	a	a	c	a	c	c	g	c
KF268127.1	5	t	g	g	c	a	g	c	a	a	c	a	c	c	g	c
M73260.1	5	t	g	g	c	a	g	c	a	a	c	a	c	c	g	c
KF951595.1	6	t	g	g	t	a	g	c	a	g	c	g	c	c	g	a
LC068713.1	6	t	g	g	t	a	g	c	a	g	c	g	c	c	g	a
LC068715.1	6	t	g	g	t	a	g	c	a	g	c	g	c	c	g	a
LC068714.1	6	t	g	g	t	a	g	c	a	g	c	g	c	c	g	a
LC068716.1	6	t	g	g	t	a	g	c	a	g	c	g	c	c	g	a
JX423389.1	6	t	g	g	t	a	g	c	a	g	c	g	c	c	g	a
HQ413315.1	6	t	g	g	t	a	g	c	a	g	c	g	c	c	g	a
FJ349096.1	6	t	g	g	t	a	g	c	a	g	c	g	c	c	g	a
HQ003817.1	1	t	g	g	t	a	g	t	a	g	c	g	c	c	g	a
KR699642.1	1	t	g	g	t	t	g	t	a	g	c	g	c	c	g	a
BJ09-2013-2	1	t	g	g	t	a	g	t	a	g	c	g	c	c	g	a
LC068720.1	1	t	g	a	t	a	g	c	a	g	c	g	c	c	g	a
LC068719.1	1	t	g	a	t	a	g	c	a	g	c	g	c	c	g	a
LC068717.1	1	t	g	a	t	a	g	c	a	g	c	g	c	c	g	a
LC068712.1	1	t	g	g	t	a	g	t	a	g	c	g	c	c	g	a
LC068718.1	1	t	g	g	t	a	g	t	a	g	c	g	c	c	g	a
BJ04-2012-2	1	t	g	a	c	a	g	t	a	g	c	g	c	c	g	a
JX173086.1	1	t	g	g	t	a	g	t	a	g	c	g	c	c	g	a
JX173085.1	1	t	g	g	t	a	g	t	a	g	c	g	c	c	g	a
JX173083.1	1	t	g	g	t	a	g	t	a	g	c	g	c	c	g	a
AF534906.1	1	t	g	g	t	a	g	t	a	g	c	g	c	c	g	a
JX173082.1	1	t	g	g	t	a	g	t	a	g	c	g	c	c	g	a
JX173078.1	1	t	g	g	t	a	g	t	a	g	c	g	c	t	g	a
JX173080.1	1	t	g	g	t	a	g	t	a	g	c	g	c	c	g	a
KF268129.1		t	g	g	t	a	g	t	a	a	c	g	c	c	g	c
KF268199.1		t	a	g	t	a	g	t	a	g	c	g	c	c	g	a
JX173079.1		t	g	g	c	a	g	c	a	a	c	g	c	c	g	a
KX384959.1		t	g	g	c	a	g	c	a	a	c	g	c	c	g	a
JX173081.1		g	g	g	c	a	a	t	c	a	t	g	t	c	g	a

1: P type is indicat
2: Nucleotide posit
3: SNPs are indica

Supplementary Ta

	P [1]	1335	1372	1413	1461	1464	1686	1701
KF268310.1	2	a	a	g	g	c	c	c
JX173077.1	2	a	a	g	g	c	c	c
KF268130.1	2	a	a	g	g	c	c	c
NC 001405	2	a	a	g	g	c	c	c
JX173084.1	2	a	a	g	g	c	c	c
KF429754.1	5	a	c	g	a	c	c	c
KF268127.1	5	a	c	g	a	c	c	c
M73260.1	5	a	c	g	a	c	c	c
KF951595.1	6	c	c	g	g	c	c	c
LC068713.1	6	c	c	g	g	c	c	c
LC068715.1	6	c	c	g	g	c	c	c
LC068714.1	6	c	c	g	g	c	c	t
LC068716.1	6	c	c	g	g	c	c	c
JX423389.1	6	c	c	g	g	c	c	c
HQ413315.1	6	c	c	g	g	c	c	c
FJ349096.1	6	c	c	g	g	c	c	c
HQ003817.1	1	c	a	g	g	c	c	c
KR699642.1	1	c	a	g	g	c	c	c
BJ09-2013-2	1	c	a	g	g	c	c	c
LC068720.1	1	c	a	g	g	c	t	c
LC068719.1	1	c	a	g	g	c	t	c
LC068717.1	1	c	a	g	g	c	c	c
LC068712.1	1	c	a	g	g	c	c	c
LC068718.1	1	c	a	g	g	c	c	c
BJ04-2012-2	1	c	a	g	g	c	c	c
JX173086.1	1	c	a	g	g	c	c	c
JX173085.1	1	c	a	g	g	t	c	c
JX173083.1	1	c	a	g	g	c	c	c
AF534906.1	1	c	a	g	g	c	c	c
JX173082.1	1	c	a	g	g	c	c	c
JX173078.1	1	c	a	g	g	c	c	c
JX173080.1	1	t	a	g	g	c	c	c
KF268129.1		c	c	g	g	c	c	c
KF268199.1		c	c	g	g	c	c	c
JX173079.1		c	c	g	g	c	c	c
KX384959.1		c	c	g	g	c	c	c
JX173081.1		c	c	a	g	t	c	c

1: P type is indicat
2: Nucleotide posit
3: SNPs are indica

Supplementary Table S3: amino acid analysis of the 37 ORFs encoded by the BJ04 genome

Protein name	Transcription unit	Length (amimo acid)	Number of conserved amino acid [1]	Number of amino acid changes				
				Total	Specific to type 2	Shared by type 2	Not shared by type 2	BJ04 specific
Control protein E1A	E1A	289	278	11	0	5	5	1
Control protein E1B 19K	E1B	178	175	3	0	3	0	0
Control protein E1B 55K	E1B	498	487	11	0	10	0	1
Capsid protein IX	IX	140	138	2	0	0	2	0
Encapsidation protein IVa2	IVa2	449	442	7	0	5	2	0
DNA polymerase	E2B	1198	1173	25	0	15	7	3
Protein 13.6K	L1	145	137	8	1	7	0	0
Terminal protein precursor pTP	E2B	671	663	8	0	2	2	4
Encapsidation protein 52K	L1	415	409	6	0	5	0	1
Capsid protein precursor pIIIa	L1	585	582	3	0	2	0	1
Penton base (capsid protein III)	L2	574	563	11	0	7	3	1
Core protein precursor pVII	L2	198	198	0	0	0	0	0
Core protein V	L2	368	362	6	0	4	2	0
Core protein precursor pX	L2	80	80	0	0	0	0	0
Capsid protein precursor pVI	L3	250	244	6	2	4	0	0
Hexon (capsid protein II)	L3	968	773	195	48	147	0	0
Protease	L3	204	201	3	0	3	0	0
Single-stranded DNA-binding protein	E2A-L	529	511	18	0	17	0	1
Hexon assembly protein 100K	L4	805	779	26	1	23	1	1
Protein 33K	L4	228	217	11	1	9	0	1
Encapsidation protein 22K	L4	195	185	10	2	7	0	1
Capsid protein precursor pVIII	L4	227	225	2	0	2	0	0
Control protein E3 12.5K	E3	107	89	18	1	16	0	1
Membrane glycoprotein E3 CR1-alpha	E3	61	45	16	0	15	1	0
Membrane glycoprotein E3 gp19K	E3	159	119	40	0	39	0	1
Membrane glycoprotein E3 CR1-beta	E3	101	60	41	1	40	0	0
Membrane protein E3 RID-alpha	E3	91	78	13	0	13	0	0
Membrane protein E3 RID-beta	E3	130	92	38	2	36	0	0
Control protein E3 14.7K	E3	128	92	36	0	36	0	0
Protein U	U	55	44	11	0	11	0	0
Fiber (capsid protein IV)	L5	582	339	243	88	154	0	1
Control protein E4orf6/7	E4	150	145	5	0	3	0	2
Control protein E4 34K	E4	294	290	4	0	0	2	2
Control protein E4orf4	E4	114	111	3	0	1	1	1
Control protein E4orf3	E4	116	114	2	0	0	1	1
Control protein E4orf2	E4	130	128	2	0	2	0	0
Control protein E4orf1	E4	128	128	0	0	0	0	0
**Total [2]**		11540	10696	844	147	643	29	25

1: BJ04 ORFs were compared to the homologs of the 5 prototypes.

2: These numbers were used to generate Figure 3A.

Supplementary Table S4: amino acid analysis of the 37 ORFs encoded by BJ09 genome

				Number of amino acid changes									
							Specific to						
									Type				
Protein name	Transcription unit	Length (Amino acid)	Amino acid conserved [1]	Total	Shared by CBJ113 and any other prototype	Shared by any prototype, not by CBJ113	CBJ113	BJ09	1	2	5	6	57
Control protein E1A	E1A	289	278	11	6	4	0	1					
Control protein E1B 19K	E1B	177	174	3	3		0						
Control protein E1B 55K	E1B	497	484	12	5	5	0	2			5		
Capsid protein IX	IX	140	138	2	2		0						
Encapsidation protein IVa2	IVa2	449	442	7	6	1	0						
DNA polymerase	E2B	1198	1174	24	15	7	0	2			1		
Protein 13.6K	L1	145	137	8	8		0			1			
Terminal protein precursor pTP	E2B	676	666	10	4	1	0	5					
Encapsidation protein 52K	L1	415	410	5	5		0						
Capsid protein precursor pIIIa	L1	585	582	3	3		0						
Penton base (capsid protein III)	L2	574	562	12	10	1	0	1					
Core protein precursor pVII	L2	198	198	0	0		0						
Core protein V	L2	368	359	9	6	2	0	1					
Core protein precursor pX	L2	80	80	0	0		0						
Capsid protein precursor pVI	L3	250	243	7	6	1	0		2				
Hexon (capsid protein II)	L3	968	773	195	195		0			48			
Protease	L3	204	200	4	3		1						1
Single-stranded DNA-binding protein	E2A-L	529	511	18	17		1		2				
Hexon assembly protein 100K	L4	806	779	27	24	2	1		2	1	1		
Protein 33K	L4	227	215	12	9	1	1	1		1			
Encapsidation protein 22K	L4	194	180	14	10	1	1	2		1			
Capsid protein precursor pVIII	L4	227	225	2	2		0						
Control protein E3 12.5K	E3	107	90	17	17		0			1			
Membrane glycoprotein E3 CR1-alpha	E3	61	45	16	15	1	0						
Membrane glycoprotein E3 gp19K	E3	159	120	39	39		0						
Membrane glycoprotein E3 CR1-beta	E3	61	45	16	16		0						
Membrane protein E3 RID-alpha	E3	91	78	13	13		0						
Membrane protein E3 RID-beta	E3	130	92	38	38		0			2			
Control protein E3 14.7K	E3	128	91	37	36	1	0						
Protein U	U	55	44	11	11		0						
Fiber (capsid protein IV)	L5	582	339	243	241	1	1			88			
Control protein E4orf6/7	E4	150	147	3	3		0				1		
Control protein E4 34K	E4	294	292	2	2		0						
Control protein E4orf4	E4	114	112	2	2		0				1		
Control protein E4orf3	E4	116	115	1	1		0				1		
Control protein E4orf2	E4	130	127	3	2	1	0						
Control protein E4orf1	E4	128	128	0	0		0						
**Total** [2]		11502	10675	826	775	30	6	15	6	143	10	0	1

1: BJ09 ORFs were compared to homologs encoded by the genomes of the 5 prototypes and CBJ113

2: These numbers were used to generate Figure 4A.

Sequence category	ORF	Control protein E1A												Control protein E1B 19K			Control protein E1B 5									
	position in ORF	29	68	74	81	94	149	204	209	210	223	264	276	157	159	160	20	25	32	41	51	53	88	98	114	116
1	AF534906	A	D	A	L	H	Q	A	M	V	R	R	P	E	A	R	F	G	T	N	T	A	F	N	F	A
2	NC 001405	A	E	V	F	H	H	T	L	V	R	Q	S	E	A	R	S	G	A	N	T	-	F	N	S	A
5	AC 000008	A	D	V	L	H	H	T	M	A	R	Q	P	Q	E	A	S	C	A	D	-	-	Y	T	C	A
6	FJ349096	A	E	V	F	H	H	T	L	V	R	Q	S	E	A	R	S	G	A	N	T	-	F	N	S	A
57	HQ003817	A	E	V	F	H	H	T	L	V	I	Q	S	E	A	R	F	G	T	N	T	A	F	N	S	A
C	KR699642	A	E	V	F	H	H	T	L	V	R	Q	S	E	A	R	S	G	A	N	-	T	F	N	S	A
4	BJ04	T	D	V	L	H	Q	T	M	V	R	Q	P	E	A	R	S	G	A	N	A	-	F	N	S	A
9	BJ09	A	D	V	L	Y	H	T	M	V	R	Q	P	E	A	R	S	G	A	N	-	-	Y	T	C	S
BJ4 type 2 only		0	0	0	0	0	0	0	0	0	0	0	0	0	0	0	0	0	0	0	0	0	0	0	0	0
BJ4 not type2		0	1	0	1	0	1	0	1	0	0	0	1	0	0	0	0	0	0	0	0	0	0	0	0	0
BJ9 any type		1	1	1	1	0	1	1	1	1	1	1	1	1	1	1	1	1	1	1	1	1	1	1	1	0
BJ9 vs CBJ113		1	0	1	0	0	1	1	0	1	1	1	0	1	1	1	1	1	1	1	1	0	0	0	0	0
BJ9 any type not CBJ113		0	1	0	1	0	0	0	1	0	0	0	1	0	0	0	0	0	0	0	0	0	1	1	1	0
BJ9 any type and CBJ113		0	0	1	0	0	1	1	0	1	1	1	0	1	1	1	1	1	1	1	0	0	0	0	0	0

Note: The ORFs core protein precursor pVII, core protein precursor pX and control protein E4orf1 are 100% conserved.

Sequence category	ORF	5K				Capsid protein IX		Encapsidation protein IVa2																		
	position in ORF	120	124	130	136	3	123	27	33	55	83	130	137	163	4	33	34	48	56	64	89	99	101	136	176	254
1	AF534906	V	T	R	V	T	E	A	H	G	H	D	R	Q	V	P	C	P	R	P	V	A	D	T	V	N
2	NC 001405	A	T	R	V	A	D	A	H	G	H	H	Q	Q	V	P	R	P	R	P	V	V	D	S	I	N
5	AC 000008	A	A	R	I	T	D	A	H	G	Q	D	R	H	A	T	R	P	G	S	V	A	D	T	V	N
6	FJ349096	A	T	R	V	A	D	T	H	G	H	D	Q	Q	V	P	R	P	R	P	V	A	D	T	V	N
57	HQ003817	V	T	R	V	T	E	A	Y	E	H	D	R	Q	V	P	R	S	R	P	V	A	N	T	V	N
C	KR699642	A	T	R	V	A	D	A	H	G	H	D	Q	Q	V	P	R	P	R	P	V	A	D	T	V	N
4	BJ04	A	T	R	V	T	E	A	H	G	H	D	R	Q	V	P	R	P	G	P	I	A	D	T	V	H
9	BJ09	A	A	K	I	A	D	A	H	G	H	D	R	Q	V	P	R	P	G	P	V	A	D	T	V	N
BJ4 type 2 only		0	0	0	0	0	0	0	0	0	0	0	0	0	0	0	0	0	0	0	0	0	0	0	0	0
BJ4 not type2		0	0	0	0	1	1	0	0	0	0	1	1	0	0	0	0	0	1	0	0	1	0	1	1	0
BJ9 any type		1	1	0	1	1	1	1	1	1	1	1	1	1	1	1	1	1	1	1	1	1	1	1	1	1
BJ9 vs CBJ113		1	0	0	0	1	1	1	1	1	1	1	0	1	1	1	1	1	0	1	1	1	1	1	1	1
BJ9 any type not CBJ113		0	1	0	1	0	0	0	0	0	0	0	1	0	0	0	0	0	1	0	0	0	0	0	0	0
BJ9 any type and CBJ113		1	0	0	0	1	1	1	1	1	1	1	0	1	1	1	1	1	0	1	0	1	1	1	1	0

Note: The ORFs core protein

Sequence category	ORF	DNA polymerase															Protein 13.6K									
	position in ORF	274	321	322	345	386	405	613	648	738	897	926	1005	1046	1112	1173	14	19	67	140	142	143	144	145	36	64
1	AF534906	R	V	D	V	N	T	S	E	I	Q	T	P	E	T	D	V	V	W	R	E	L	L	Q	P	A
2	NC 001405	R	V	D	I	S	T	S	D	V	E	S	P	E	T	D	V	V	W	L	H	R	G	T	P	A
5	AC 000008	R	A	N	I	S	I	S	D	V	Q	S	P	E	T	D	I	I	W	L	R	R	G	T	P	A
6	FJ349096	R	A	D	I	S	I	S	D	V	E	S	P	E	T	D	V	V	W	R	E	L	L	Q	P	A
57	HQ003817	R	V	D	I	S	T	S	D	I	Q	T	P	N	I	N	V	V	G	R	E	L	L	Q	S	A
C	KR699642	R	A	D	I	S	I	S	D	V	E	S	P	K	T	D	V	V	W	L	H	R	G	T	P	A
4	BJ04	C	V	D	I	S	T	S	D	I	Q	T	P	E	T	D	V	V	W	L	H	R	G	T	P	T
9	BJ09	R	V	D	I	S	T	N	D	I	Q	T	H	E	T	D	V	V	W	L	H	R	G	T	P	A
BJ4 type 2 only		0	0	0	0	0	0	0	0	0	0	0	0	0	0	0	0	0	0	0	1	0	0	0	0	0
BJ4 not type2		0	0	0	0	0	0	0	0	1	1	1	0	0	0	0	0	0	0	0	0	0	0	0	0	0
BJ9 any type		1	1	1	1	1	1	0	1	1	1	1	0	1	1	1	1	1	1	1	1	1	1	1	1	1
BJ9 vs CBJ113		1	0	1	1	1	0	0	1	0	0	0	0	0	1	1	1	1	1	1	1	1	1	1	1	1
BJ9 any type not CBJ113		0	1	0	0	0	1	0	0	1	1	1	0	1	0	0	0	0	0	0	0	0	0	0	0	0
BJ9 any type and CBJ113		0	0	1	1	1	0	0	1	0	0	0	0	0	1	1	1	1	1	1	1	1	1	1	1	0

Note: The ORFs core protein

Sequence category	ORF	Terminal protein precursor pTP												Encapsidation protein 52K						Capsid protein precu	
	position in ORF	195	278	283	301	398	399	400	401	402	432	527	612	30	41	49	56	74	79	88	108
1	AF534906	A	R	A	R	-	-	-	-	-	D	A	F	S	A	Y	A	P	H	P	G
2	NC 001405	V	H	A	R	-	-	-	-	-	D	A	F	S	A	Y	A	P	H	P	G
5	AC 000008	A	R	A	R	-	-	-	-	-	D	A	L	P	V	Y	A	P	Y	P	G
6	FJ349096	A	R	A	R	-	-	-	-	-	D	A	F	S	A	Y	T	P	H	P	S
57	HQ003817	A	R	A	R	-	-	-	-	-	D	A	F	S	A	D	A	P	H	S	G
C	KR699642	A	R	A	K	-	-	-	-	-	D	A	F	S	A	Y	A	P	H	P	G
4	BJ04	A	R	T	R	-	-	-	-	-	N	T	F	S	A	Y	A	S	H	P	G
9	BJ09	A	R	A	R	E	E	E	E	E	D	A	F	S	A	Y	A	P	H	P	G
BJ4 type 2 only		0	0	0	0	0	0	0	0	0	0	0	0	0	0	0	0	0	0	0	0
BJ4 not type2		1	1	0	0	0	0	0	0	0	0	0	0	0	0	0	0	0	0	0	0
BJ9 any type		1	1	1	1	0	0	0	0	0	1	1	1	1	1	1	1	1	1	1	1
BJ9 vs CBJ113		1	1	1	0	0	0	0	0	0	1	1	1	1	1	1	1	1	1	1	1
BJ9 any type not CBJ113		0	0	0	1	0	0	0	0	0	0	0	0	0	0	0	0	0	0	0	0
BJ9 any type and CBJ113		1	1	0	0	0	0	0	0	0	0	0	1	1	1	1	1	0	1	1	1

Note: The ORFs core protein

Sequence category	ORF	ursor pIIIa	Penton base (capsid protein III)													Core protein V									Ca
	position in ORF	474	2	23	152	153	157	305	310	312	332	368	386	420	458	73	80	152	250	255	283	289	326	342	50
1	AF534906	L	R	P	S	L	K	G	G	N	E	A	T	A	S	R	T	A	A	A	R	A	I	A	T
2	NC 001405	F	Q	P	S	L	K	D	G	N	E	-	T	T	S	R	T	T	A	A	R	T	T	A	T
5	AC 000008	L	R	P	L	P	N	G	S	S	E	-	T	T	R	K	T	A	A	V	G	A	T	A	T
6	FJ349096	L	R	P	S	L	K	G	G	N	E	A	T	A	R	R	T	A	A	A	R	A	T	A	A
57	HQ003817	L	R	P	S	L	K	G	G	N	D	A	T	A	S	R	T	A	A	A	R	A	T	A	T
C	KR699642	L	R	P	S	L	K	G	G	N	E	A	S	A	S	R	S	A	A	A	R	A	T	T	T
4	BJ04	V	R	P	S	L	K	G	G	N	E	T	T	A	S	R	T	A	A	A	R	A	T	A	T
9	BJ09	L	R	S	S	L	K	G	G	N	E	A	T	A	S	R	T	A	T	A	R	A	T	A	T
BJ4 type 2 only		0	0	0	0	0	0	0	0	0	0	0	0	0	0	0	0	0	0	0	0	0	0	0	0
BJ4 not type2		0	1	0	0	0	0	1	0	0	0	0	0	1	0	0	0	1	0	0	0	1	0	0	0
BJ9 any type		1	1	0	1	1	1	1	1	1	1	1	1	1	1	1	1	1	0	1	1	1	1	1	1
BJ9 vs CBJ113		1	1	0	1	1	1	1	1	1	1	1	0	1	1	1	0	1	0	1	1	1	1	0	1
BJ9 any type not CBJ113		0	0	0	0	0	0	0	0	0	0	0	1	0	0	0	1	0	0	0	0	0	0	1	0
BJ9 any type and CBJ113		1	1	0	1	1	1	1	1	1	1	0	0	1	1	1	0	1	0	1	1	1	1	0	1

Note: The ORFs core protein

Sequence category	ORF	psid protein precursor pVI																								
	position in ORF	51	119	124	126	199	221	132	136	137	138	139	140	141	142	143	144	145	146	147	148	149	150	151	152	153
1	AF534906	V	A	L	D	S	L	S	E	Q	E	E	P	T	Q	E	M	A	E	E	L	E	D	E	E	E
2	NC 001405	I	A	S	E	S	M	S	E	Q	T	E	D	S	G	R	A	V	A	-	-	E	D	E	E	E
5	AC 000008	V	A	S	E	T	L	P	D	E	A	A	T	A	L	E	I	N	L	-	-	E	E	E	D	D
6	FJ349096	V	A	S	E	S	L	S	E	Q	N	E	T	A	Q	-	-	-	-	-	-	V	D	A	Q	E
57	HQ003817	V	A	S	E	S	L	S	D	E	D	D	T	Q	V	Q	V	A	A	-	-	E	D	D	Q	D
C	KR699642	V	V	L	D	S	L	S	E	Q	T	E	D	S	G	R	A	V	A	-	-	E	D	E	E	E
4	BJ04	I	A	S	E	S	M	S	E	Q	T	E	D	S	G	R	A	V	A	-	-	E	D	E	E	E
9	BJ09	V	A	L	D	S	L	S	E	Q	T	E	D	S	G	R	A	V	A	-	-	E	D	E	E	E
BJ4 type 2 only		1	0	0	0	0	1	0	0	0	1	0	1	1	1	1	1	1	0	0	0	0	0	0	0	0
BJ4 not type2		0	0	0	0	0	0	0	0	0	0	0	0	0	0	0	0	0	0	0	0	0	0	0	0	0
BJ9 any type		1	1	1	1	1	1	1	1	1	1	1	1	1	1	1	1	1	1	1	1	1	1	1	1	1
BJ9 vs CBJ113		1	0	1	1	1	1	1	1	1	1	1	1	1	1	1	1	1	1	1	1	1	1	1	1	1
BJ9 any type not CBJ113		0	1	0	0	0	0	0	0	0	0	0	0	0	0	0	0	0	0	0	0	0	0	0	0	0
BJ9 any type and CBJ113		1	0	1	1	1	1	1	1	1	1	1	1	1	1	1	1	1	1	0	0	1	1	1	1	1

Note: The ORFs core protein

Sequence category	ORF																								
	position in ORF	154	155	157	159	160	161	162	163	164	165	166	167	168	169	170	171	172	173	178	179	183	184	186	187
1	AF534906	A	E	E	A	E	E	E	A	E	A	P	Q	A	D	Q	K	V	K	Y	A	L	A	E	K
2	NC 001405	E	D	D	E	E	E	E	E	E	Q	N	A	R	D	Q	A	T	K	Y	A	L	S	E	T
5	AC 000008	D	N	D	V	D	E	Q	A	E	Q	-	-	-	-	-	-	-	Q	F	G	Y	S	I	N
6	FJ349096	L	D	E	N	E	A	N	E	A	Q	A	R	E	Q	E	Q	A	K	Y	A	L	S	I	K
57	HQ003817	D	D	E	E	Q	L	P	Q	Q	R	N	G	-	-	-	-	-	K	Y	A	F	A	E	A
C	KR699642	E	D	D	E	E	E	E	E	E	Q	N	A	R	D	Q	A	T	K	Y	A	L	S	E	T
4	BJ04	E	D	D	E	E	E	E	E	E	Q	N	A	R	D	Q	A	T	K	Y	A	L	S	E	T
9	BJ09	E	D	D	E	E	E	E	E	E	Q	N	A	R	D	Q	A	T	K	Y	A	L	S	E	T
BJ4 type 2 only		1	0	0	0	0	0	0	0	0	0	0	1	1	0	0	1	1	0	0	0	0	0	0	1
BJ4 not type2		0	0	0	0	0	0	0	0	0	0	0	0	0	0	0	0	0	0	0	0	0	0	0	0
BJ9 any type		1	1	1	1	1	1	1	1	1	1	1	1	1	1	1	1	1	1	1	1	1	1	1	1
BJ9 vs CBJ113		1	1	1	1	1	1	1	1	1	1	1	1	1	1	1	1	1	1	1	1	1	1	1	1
BJ9 any type not CBJ113		0	0	0	0	0	0	0	0	0	0	0	0	0	0	0	0	0	0	0	0	0	0	0	0
BJ9 any type and CBJ113		1	1	1	1	1	1	1	1	1	1	1	1	1	1	1	1	1	1	1	1	1	1	1	1

Note: The ORFs core protein

Sequence category	ORF	189	190	191	193	196	197	198	199	200	201	202	203	204	205	206	207	208	209	210	213	214	215	221	227
														Loop 1											
	position in ORF	189	190	191	193	196	197	198	199	200	201	202	203	204	205	206	207	208	209	210	213	214	215	221	227
1	AF534906	T	A	N	L	V	S	D	-	T	Q	T	E	G	-	-	N	P	V	F	P	T	Y	V	N
2	NC 001405	T	K	S	L	G	S	D	N	A	E	T	Q	A	-	-	K	P	V	Y	P	S	Y	I	N
5	AC 000008	T	K	E	I	G	V	E	G	Q	-	-	-	-	-	-	T	P	K	Y	K	T	F	I	Y
6	FJ349096	T	K	E	L	G	T	A	D	A	T	V	A	G	A	G	K	E	I	F	K	T	F	V	N
57	HQ003817	N	K	N	L	G	T	N	G	A	A	T	E	G	-	N	K	E	I	Y	K	T	Y	I	N
C	KR699642	T	K	S	L	G	S	D	N	A	E	T	Q	A	-	-	K	P	V	Y	P	S	Y	I	N
4	BJ04	T	K	S	L	G	S	D	N	A	E	T	Q	A	-	-	K	P	V	Y	P	S	Y	I	N
9	BJ09	T	K	S	L	G	S	D	N	A	E	T	Q	A	-	-	K	P	V	Y	P	S	Y	I	N
BJ4 type 2 only		0	0	1	0	0	0	0	1	0	1	0	1	1	0	0	0	0	0	0	0	1	0	0	0
BJ4 not type2		0	0	0	0	0	0	0	0	0	0	0	0	0	0	0	0	0	0	0	0	0	0	0	0
BJ9 any type		1	1	1	1	1	1	1	1	1	1	1	1	1	1	1	1	1	1	1	1	1	1	1	1
BJ9 vs CBJ113		1	1	1	1	1	1	1	1	1	1	1	1	1	1	1	1	1	1	1	1	1	1	1	1
BJ9 any type not CBJ113		0	0	0	0	0	0	0	0	0	0	0	0	0	0	0	0	0	0	0	0	0	0	0	0
BJ9 any type and CBJ113		1	1	1	1	1	1	1	1	1	1	1	1	1	0	0	1	1	1	1	1	1	1	1	1

Note: The ORFs core protein

Sequence category	ORF																								
	position in ORF	229	230	231	232	233	234	235	254	258	259	263	264	265	267	268	269	270	272	273	274	275	276	278	279
1	AF534906	A	E	A	T	A	S	G	R	K	N	G	I	L	A	N	N	Q	A	L	E	S	K	E	M
2	NC 001405	A	D	A	N	A	A	G	R	P	F	S	V	L	P	D	E	K	V	P	L	P	K	D	L
5	AC 000008	T	E	I	N	H	A	A	K	E	N	G	I	L	K	Q	Q	N	K	L	E	S	Q	E	M
6	FJ349096	A	D	A	T	A	A	G	R	S	N	G	V	M	-	E	Q	N	K	L	E	S	Q	E	M
57	HQ003817	A	E	S	S	V	A	G	R	S	N	G	V	M	-	E	Q	N	K	L	E	S	Q	E	M
C	KR699642	A	D	A	N	A	A	G	R	P	F	S	V	L	P	D	E	K	V	P	L	P	K	D	L
4	BJ04	A	D	A	N	A	A	G	R	P	F	S	V	L	P	D	E	K	V	P	L	P	K	D	L
9	BJ09	A	D	A	N	A	A	G	R	P	F	S	V	L	P	D	E	K	V	P	L	P	K	D	L
BJ4 type 2 only		0	0	0	0	0	0	0	0	1	1	1	0	0	1	1	1	1	1	1	1	1	0	1	1
BJ4 not type2		0	0	0	0	0	0	0	0	0	0	0	0	0	0	0	0	0	0	0	0	0	0	0	0
BJ9 any type		1	1	1	1	1	1	1	1	1	1	1	1	1	1	1	1	1	1	1	1	1	1	1	1
BJ9 vs CBJ113		1	1	1	1	1	1	1	1	1	1	1	1	1	1	1	1	1	1	1	1	1	1	1	1
BJ9 any type not CBJ113		0	0	0	0	0	0	0	0	0	0	0	0	0	0	0	0	0	0	0	0	0	0	0	0
BJ9 any type and CBJ113		1	1	1	1	1	1	1	1	1	1	1	1	1	1	1	1	1	1	1	1	1	1	1	1

Note: The ORFs core protein

Sequence category	ORF	Hexon (capsid protein II)																							
	position in ORF	283	284	285	286	287	288	289	290	292	293	294	295	296	297	299	300	308	309	316	319	321	322	323	324
1	AF534906	A	P	S	G	T	A	M	N	E	R	N	A	V	Q	S	I	N	M	I	K	S	K	T	D
2	NC 001405	S	N	T	T	S	L	N	D	Q	G	N	A	T	K	K	V	N	M	L	K	G	K	G	D
5	AC 000008	S	T	T	E	A	T	A	G	N	G	D	N	L	T	K	V	D	I	I	M	T	I	K	E
6	FJ349096	S	T	S	T	N	A	T	N	E	V	N	N	I	Q	T	V	N	M	L	K	K	M	G	D
57	HQ003817	S	T	S	V	N	A	M	N	E	A	N	A	I	Q	K	L	N	M	L	K	G	K	S	D
C	KR699642	S	N	T	T	S	L	N	D	Q	G	N	A	T	K	K	V	N	M	L	K	G	K	G	D
4	BJ04	S	N	T	T	S	L	N	D	Q	G	N	A	T	K	K	V	N	M	L	K	G	K	G	D
9	BJ09	S	N	T	T	S	L	N	D	Q	G	N	A	T	K	K	V	N	M	L	K	G	K	G	D
BJ4 type 2 only		0	1	0	0	1	1	1	1	1	0	0	0	1	1	0	0	0	0	0	0	0	0	0	0
BJ4 not type2		0	0	0	0	0	0	0	0	0	0	0	0	0	0	0	0	0	0	0	0	0	0	0	0
BJ9 any type		1	1	1	1	1	1	1	1	1	1	1	1	1	1	1	1	1	1	1	1	1	1	1	1
BJ9 vs CBJ113		1	1	1	1	1	1	1	1	1	1	1	1	1	1	1	1	1	1	1	1	1	1	1	1
BJ9 any type not CBJ113		0	0	0	0	0	0	0	0	0	0	0	0	0	0	0	0	0	0	0	0	0	0	0	0
BJ9 any type and CBJ113		1	1	1	1	1	1	1	1	1	1	1	1	1	1	1	1	1	1	1	1	1	1	1	1

Note: The ORFs core protein

Sequence category	ORF																								
																				Loop 2					
	position in ORF	325	327	328	329	330	331	335	436	437	438	440	442	443	444	445	447	448	450	451	452	454	455	456	457
1	AF534906	E	S	K	A	M	L	A	I	G	V	D	Y	Q	G	I	S	N	-	-	-	N	G	N	P
2	NC 001405	E	S	K	A	M	L	S	I	G	V	D	Y	Q	A	I	A	N	N	G	S	D	N	G	D
5	AC 000008	G	S	R	E	L	M	S	V	I	N	E	L	T	K	V	P	K	-	-	-	T	G	Q	E
6	FJ349096	K	A	K	V	M	L	A	I	G	I	D	F	Q	A	V	T	T	A	A	N	D	Q	G	N
57	HQ003817	D	S	K	A	M	L	S	I	G	V	D	Y	Q	A	I	A	T	-	-	N	N	G	G	A
C	KR699642	E	S	K	A	M	L	S	I	G	V	D	Y	Q	A	I	A	N	N	G	S	D	N	G	D
4	BJ04	E	S	K	A	M	L	S	I	G	V	D	Y	Q	A	I	A	N	N	G	S	D	N	G	D
9	BJ09	E	S	K	A	M	L	S	I	G	V	D	Y	Q	A	I	A	N	N	G	S	D	N	G	D
BJ4 type 2 only		0	0	0	0	0	0	0	0	0	0	0	0	0	0	0	0	0	1	1	1	0	1	0	1
BJ4 not type2		0	0	0	0	0	0	0	0	0	0	0	0	0	0	0	0	0	0	0	0	0	0	0	0
BJ9 any type		1	1	1	1	1	1	1	1	1	1	1	1	1	1	1	1	1	1	1	1	1	1	1	1
BJ9 vs CBJ113		1	1	1	1	1	1	1	1	1	1	1	1	1	1	1	1	1	1	1	1	1	1	1	1
BJ9 any type not CBJ113		0	0	0	0	0	0	0	0	0	0	0	0	0	0	0	0	0	0	0	0	0	0	0	0
BJ9 any type and CBJ113		1	1	1	1	1	1	1	1	1	1	1	1	1	1	1	1	1	1	1	1	1	1	1	1

Note: The ORFs core protein

Sequence category	ORF																								
	position in ORF	458	459	461	462	463	464	465	466	468	469	470	474	481	509	511	514	517	521	834	841	867	926	927	928
1	AF534906	Q	N	T	K	N	D	D	-	A	A	R	G	L	T	T	E	P	S	D	L	F	F	E	V
2	NC 001405	T	T	T	K	D	E	T	-	A	T	R	G	M	N	T	E	D	T	E	L	V	F	E	V
5	AC 000008	N	G	E	K	D	A	T	E	S	D	K	R	M	S	S	K	D	T	D	L	F	F	E	V
6	FJ349096	T	T	Q	K	D	S	T	-	A	E	R	G	M	N	T	E	D	T	D	I	V	L	R	W
57	HQ003817	T	T	A	Q	D	N	T	-	A	E	R	G	M	N	T	E	D	T	D	L	F	F	E	V
C	KR699642	T	T	T	K	D	E	T	-	A	T	R	G	M	N	T	E	D	T	E	L	V	F	E	V
4	BJ04	T	T	T	K	D	E	T	-	A	T	R	G	M	N	T	E	D	T	E	L	V	F	E	V
9	BJ09	T	T	T	K	D	E	T	-	A	T	R	G	M	N	T	E	D	T	E	L	V	F	E	V
BJ4 type 2 only		0	0	0	0	0	1	0	0	0	1	0	0	0	0	0	0	0	0	1	0	0	0	0	0
BJ4 not type2		0	0	0	0	0	0	0	0	0	0	0	0	0	0	0	0	0	0	0	0	0	0	0	0
BJ9 any type		1	1	1	1	1	1	1	1	1	1	1	1	1	1	1	1	1	1	1	1	1	1	1	1
BJ9 vs CBJ113		1	1	1	1	1	1	1	1	1	1	1	1	1	1	1	1	1	1	1	1	1	1	1	1
BJ9 any type not CBJ113		0	0	0	0	0	0	0	0	0	0	0	0	0	0	0	0	0	0	0	0	0	0	0	0
BJ9 any type and CBJ113		1	1	1	1	1	1	1	0	1	1	1	1	1	1	1	1	1	1	1	1	1	1	1	1

Note: The ORFs core protein

Sequence category	ORF																								
	position in ORF	929	931	932	933	935	936	937	938	939	940	941	942	943	944	945	946	947	948	949	950	951	952	953	954
1	AF534906	D	M	D	E	T	L	L	Y	V	L	F	E	V	F	D	-	-	-	-	-	-	-	-	-
2	NC 001405	D	M	D	E	T	L	L	Y	V	L	F	E	V	F	D	-	-	-	-	-	-	-	-	-
5	AC 000008	D	M	D	E	T	L	L	Y	V	L	F	E	V	F	D	-	-	-	-	-	-	-	-	-
6	FJ349096	I	W	T	S	P	F	F	M	F	C	L	K	S	L	T	W	S	V	C	T	S	R	T	A
57	HQ003817	D	M	D	E	T	L	L	Y	V	L	F	E	V	F	D	-	-	-	-	-	-	-	-	-
C	KR699642	D	M	D	E	T	L	L	Y	V	L	F	E	V	F	D	-	-	-	-	-	-	-	-	-
4	BJ04	D	M	D	E	T	L	L	Y	V	L	F	E	V	F	D	-	-	-	-	-	-	-	-	-
9	BJ09	D	M	D	E	T	L	L	Y	V	L	F	E	V	F	D	-	-	-	-	-	-	-	-	-
BJ4 type 2 only		0	0	0	0	0	0	0	0	0	0	0	0	0	0	0	0	0	0	0	0	0	0	0	0
BJ4 not type2		0	0	0	0	0	0	0	0	0	0	0	0	0	0	0	0	0	0	0	0	0	0	0	0
BJ9 any type		1	1	1	1	1	1	1	1	1	1	1	1	1	1	1	1	1	1	1	1	1	1	1	1
BJ9 vs CBJ113		1	1	1	1	1	1	1	1	1	1	1	1	1	1	1	1	1	1	1	1	1	1	1	1
BJ9 any type not CBJ113		0	0	0	0	0	0	0	0	0	0	0	0	0	0	0	0	0	0	0	0	0	0	0	0
BJ9 any type and CBJ113		1	1	1	1	1	1	1	1	1	1	1	1	1	1	1	0	0	0	0	0	0	0	0	0

Note: The ORFs core protein

Sequence category	ORF																								
	position in ORF	955	956	957	958	959	960	961	962	963	964	966	968	969	970	971	972	973	974	976	977	978	979	980	981
1	AF534906	-	-	-	-	V	V	R	V	H	Q	H	G	V	I	-	-	-	-	T	V	Y	L	R	T
2	NC 001405	-	-	-	-	V	V	R	V	H	Q	H	G	V	I	-	-	-	-	T	V	Y	L	R	T
5	AC 000008	-	-	-	-	V	V	R	V	H	R	H	G	V	I	-	-	-	-	T	V	Y	L	R	T
6	FJ349096	A	S	S	R	P	C	T	C	A	R	S	P	A	T	P	Q	H	K	A	S	N	I	N	N
57	HQ003817	-	-	-	-	V	V	R	V	H	Q	H	G	V	I	-	-	-	-	T	V	Y	L	R	T
C	KR699642	-	-	-	-	V	V	R	V	H	Q	H	G	V	I	-	-	-	-	T	V	Y	L	R	T
4	BJ04	-	-	-	-	V	V	R	V	H	Q	H	G	V	I	-	-	-	-	T	V	Y	L	R	T
9	BJ09	-	-	-	-	V	V	R	V	H	Q	H	G	V	I	-	-	-	-	T	V	Y	L	R	T
BJ4 type 2 only		0	0	0	0	0	0	0	0	0	0	0	0	0	0	0	0	0	0	0	0	0	0	0	0
BJ4 not type2		0	0	0	0	0	0	0	0	0	0	0	0	0	0	0	0	0	0	0	0	0	0	0	0
BJ9 any type		1	1	1	1	1	1	1	1	1	1	1	1	1	1	1	1	1	1	1	1	1	1	1	1
BJ9 vs CBJ113		1	1	1	1	1	1	1	1	1	1	1	1	1	1	1	1	1	1	1	1	1	1	1	1
BJ9 any type not CBJ113		0	0	0	0	0	0	0	0	0	0	0	0	0	0	0	0	0	0	0	0	0	0	0	0
BJ9 any type and CBJ113		0	0	0	0	1	1	1	1	1	1	1	1	1	1	0	0	0	0	1	1	1	1	1	1

Note: The ORFs core protein

Sequence category	ORF							Protease				Single-stranded DNA-binding protein																
	position in ORF	982	983	984	985	987	988	9	61	63	187	47	51	67	77	82	84	95	153	157	160	167	170	175	236	327	373	377
1	AF534906	P	F	S	A	N	A	K	N	R	S	L	L	R	A	N	P	P	M	E	P	S	M	L	S	S	Q	K
2	NC 001405	P	F	S	A	N	A	K	N	R	S	L	L	R	T	I	S	P	T	K	S	S	I	L	S	N	K	Q
5	AC 000008	P	F	S	A	N	A	K	N	H	S	M	I	R	A	I	P	P	T	E	P	I	M	V	S	N	K	Q
6	FJ349096	S	C	R	H	L	Q	K	N	R	S	L	L	R	T	I	S	P	M	K	S	S	I	L	S	N	K	Q
57	HQ003817	P	F	S	A	N	A	K	D	R	R	L	L	R	A	N	P	S	T	E	P	S	M	L	S	N	K	Q
C	KR699642	P	F	S	A	N	A	R	D	R	S	L	L	R	A	N	P	P	M	E	P	S	M	L	T	N	Q	K
4	BJ04	P	F	S	A	N	A	K	N	R	S	L	L	C	T	I	S	P	T	K	S	S	I	L	S	N	K	Q
9	BJ09	P	F	S	A	N	A	R	D	R	S	L	L	R	A	N	P	P	M	E	P	S	M	L	T	N	Q	K
BJ4 type 2 only		0	0	0	0	0	0	0	0	0	0	0	0	0	0	0	0	0	0	0	0	0	0	0	0	0	0	0
BJ4 not type2		0	0	0	0	0	0	0	0	0	0	0	0	0	0	0	0	0	0	0	0	0	0	0	0	0	0	0
BJ9 any type		1	1	1	1	1	1	0	1	1	1	1	1	1	1	1	1	1	1	1	1	1	1	1	0	1	1	1
BJ9 vs CBJ113		1	1	1	1	1	1	1	1	1	1	1	1	1	1	1	1	1	1	1	1	1	1	1	1	1	1	1
BJ9 any type not CBJ113		0	0	0	0	0	0	0	0	0	0	0	0	0	0	0	0	0	0	0	0	0	0	0	0	0	0	0
BJ9 any type and CBJ113		1	1	1	1	1	1	0	1	1	1	1	1	0	1	1	1	1	1	1	1	1	1	1	0	1	1	1

Note: The ORFs core protein

Sequence category	ORF			Hexon assembly protein 100K																							
	position in ORF	442	473	7	14	44	72	77	85	122	195	209	234	239	240	290	307	319	351	352	354	442	586	747	753	787	788
1	AF534906	H	G	E	S	T	D	K	E	A	A	D	S	H	C	Q	P	E	T	D	K	R	S	H	E	S	K
2	NC 001405	H	A	E	F	I	D	E	Q	A	A	D	S	H	C	R	P	E	A	D	E	R	S	D	Q	S	Q
5	AC 000008	H	A	K	S	I	N	E	E	A	A	N	S	H	S	Q	P	Q	A	D	E	C	N	H	E	F	Q
6	FJ349096	H	A	E	F	I	D	E	Q	A	A	D	S	H	C	R	P	E	A	D	E	R	S	D	Q	S	Q
57	HQ003817	N	A	E	S	I	D	E	Q	A	T	D	N	Y	S	R	P	Q	A	N	E	R	S	D	Q	S	Q
C	KR699642	H	A	E	F	T	D	E	Q	D	A	D	S	H	C	R	L	E	A	D	K	R	S	H	E	S	Q
4	BJ04	H	A	E	F	I	D	E	Q	T	A	D	S	H	C	R	P	E	A	D	E	R	S	D	Q	S	Q
9	BJ09	H	A	E	F	T	N	E	Q	D	A	D	S	H	C	R	P	E	A	D	K	R	S	H	E	S	Q
BJ4 type 2 only		0	0	0	0	0	0	0	0	0	0	0	0	0	0	0	0	0	0	0	0	0	0	0	0	0	0
BJ4 not type2		0	0	0	0	0	0	0	0	0	0	0	0	0	0	0	0	0	0	0	0	0	0	0	0	0	0
BJ9 any type		1	1	1	1	1	1	1	1	0	1	1	1	1	1	1	1	1	1	1	1	1	1	1	1	1	1
BJ9 vs CBJ113		1	1	1	1	1	0	1	1	1	1	1	1	1	1	1	0	1	1	1	1	1	1	1	1	1	1
BJ9 any type not CBJ113		0	0	0	0	0	1	0	0	0	0	0	0	0	0	0	1	0	0	0	0	0	0	0	0	0	0
BJ9 any type and CBJ113		1	1	1	1	1	0	1	1	0	1	1	1	1	1	1	0	1	1	1	1	1	1	1	1	1	1

Note: The ORFs core protein

Sequence category	ORF				Encapsidation protein 22K													Protein 33K												
	position in ORF	790	803	808	36	41	50	61	72	75	76	77	78	108	118	120	122	36	41	50	61	72	75	76	77	78	130	136	141	185
1	AF534906	G	R	P	M	E	A	S	S	G	P	S	M	P	A	A	A	M	E	A	S	S	G	P	S	M	G	V	D	S
2	NC 001405	R	C	P	M	D	A	S	L	V	P	S	I	P	A	A	V	M	D	A	S	L	V	P	S	I	G	V	D	S
5	AC 000008	G	R	P	M	E	V	S	S	G	S	S	M	H	A	A	A	M	E	V	S	S	G	S	S	M	G	V	D	S
6	FJ349096	C	R	P	M	D	A	S	S	V	P	S	I	P	A	A	A	M	D	A	S	S	V	P	S	I	V	V	D	S
57	HQ003817	C	R	S	T	D	A	S	S	V	P	S	I	P	A	A	A	T	D	A	S	S	V	P	S	I	G	V	E	S
C	KR699642	R	R	P	M	E	A	S	L	V	P	S	I	P	T	S	A	M	E	A	S	L	V	P	S	I	G	V	D	N
4	BJ04	R	R	P	M	D	A	S	L	V	P	N	I	P	A	A	V	M	D	A	S	L	V	P	N	I	G	V	D	S
9	BJ09	R	R	P	M	E	A	L	L	V	P	S	I	P	A	S	A	M	E	A	L	L	V	P	S	I	G	I	D	S
BJ4 type 2 only		1	0	0	0	0	0	0	1	0	0	0	0	0	0	0	1	0	0	0	0	1	0	0	0	0	0	0	0	0
BJ4 not type2		0	1	0	0	0	0	0	0	0	0	0	0	0	0	0	0	0	0	0	0	0	0	0	0	0	0	0	0	0
BJ9 any type		1	1	1	1	1	1	0	1	1	1	1	1	1	1	0	1	1	1	1	0	1	1	1	1	1	1	0	1	1
BJ9 vs CBJ113		1	1	1	1	1	1	0	1	1	1	1	1	1	0	1	1	1	1	1	0	1	1	1	1	1	1	0	1	0
BJ9 any type not CBJ113		0	0	0	0	0	0	0	0	0	0	0	0	0	1	0	0	0	0	0	0	0	0	0	0	0	0	0	0	1
BJ9 any type and CBJ113		1	1	1	1	1	1	0	1	1	1	0	1	1	0	0	1	1	1	1	0	1	1	1	0	1	1	0	1	0

Note: The ORFs core protein

Sequence category	ORF			Capsid protein precursor pVIII		Control protein E3 12.5K																					
	position in ORF	187	191	127	160	2	8	12	15	26	31	38	50	51	53	56	65	73	77	78	82	88	100	2	5	9	10
1	AF534906	S	T	K	S	T	Q	T	D	G	C	E	T	T	V	T	L	E	C	V	V	P	T	M	T	G	F
2	NC 001405	S	T	K	S	T	R	T	D	G	C	E	T	T	V	T	K	E	C	V	V	P	A	S	S	S	L
5	AC 000008	S	T	T	P	L	Q	K	V	D	C	D	T	A	G	A	Q	D	C	V	I	L	A	N	S	G	Y
6	FJ349096	S	A	K	S	T	Q	T	D	G	C	E	T	T	V	T	K	E	V	F	-	-	-	S	S	S	L
57	HQ003817	S	A	K	S	T	Q	T	D	D	S	E	T	T	V	T	K	E	C	V	V	P	A	S	S	S	L
C	KR699642	I	T	K	S	T	R	T	D	G	C	E	T	T	V	T	K	E	C	V	V	P	A	S	S	S	L
4	BJ04	S	T	K	S	T	R	T	D	G	C	E	I	T	V	T	K	E	C	V	V	P	A	S	S	S	L
9	BJ09	I	T	K	S	T	R	T	D	G	C	E	T	T	V	T	K	E	C	V	V	P	A	S	S	S	L
BJ4 type 2 only		0	0	0	0	0	1	0	0	0	0	0	0	0	0	0	0	0	0	0	0	0	0	0	0	0	0
BJ4 not type2		0	0	0	0	0	0	0	0	0	0	0	0	0	0	0	0	0	0	0	0	0	0	0	0	0	0
BJ9 any type		0	1	1	1	1	1	1	1	1	1	1	1	1	1	1	1	1	1	1	1	1	1	1	1	1	1
BJ9 vs CBJ113		1	1	1	1	1	1	1	1	1	1	1	1	1	1	1	1	1	1	1	1	1	1	1	1	1	1
BJ9 any type not CBJ113		0	0	0	0	0	0	0	0	0	0	0	0	0	0	0	0	0	0	0	0	0	0	0	0	0	0
BJ9 any type and CBJ113		0	1	1	1	1	1	1	1	1	1	1	0	1	1	1	1	1	1	1	1	1	1	1	1	1	1

Note: The ORFs core protein

Sequence category	ORF	Membrane glycoprotein E3 CR1-alpha														Membran																			
	position in ORF	12	14	15	18	21	26	33	36	42	47	49	51	56	61	2	3	5	8	9	11	14	15	16	17	18	19	20	21	23	34	35	54	59	61
1	AF534906	A	G	N	G	L	T	F	A	L	A	V	I	I	R	I	K	I	S	L	T	A	V	C	S	A	A	K	Q	E	K	S	I	D	A
2	NC 001405	-	-	N	G	V	T	L	A	V	T	V	T	F	Q	M	R	M	G	L	A	A	V	C	S	A	A	K	K	E	K	S	I	D	I
5	AC 000008	N	-	G	R	V	C	L	T	L	V	I	I	F	R	I	R	I	G	L	T	S	A	H	G	T	T	Q	K	D	A	A	L	N	I
6	FJ349096	-	-	N	G	V	T	L	A	V	T	V	T	F	R	M	R	M	G	L	A	A	V	C	S	A	A	K	K	E	K	S	I	D	I
57	HQ003817	-	-	N	G	V	T	L	A	V	T	V	I	F	R	M	R	I	C	M	A	A	V	C	S	A	A	K	K	E	K	S	I	D	I
C	KR699642	-	-	N	E	V	T	L	A	V	T	I	T	F	R	M	R	M	G	L	A	A	V	C	S	A	A	K	K	E	K	S	I	D	I
4	BJ04	-	-	N	G	V	T	L	A	V	T	V	T	F	R	M	R	M	G	L	A	A	V	C	S	A	A	K	K	E	K	S	I	D	I
9	BJ09	-	-	N	G	V	T	L	A	V	T	I	T	F	R	M	R	M	G	L	A	A	V	C	S	A	A	K	K	E	K	S	I	D	I
BJ4 type 2 only		0	0	0	0	0	0	0	0	0	0	0	0	0	0	0	0	0	0	0	0	0	0	0	0	0	0	0	0	0	0	0	0	0	0
BJ4 not type2		0	0	0	0	0	0	0	0	0	0	0	0	0	1	0	0	0	0	0	0	0	0	0	0	0	0	0	0	0	0	0	0	0	0
BJ9 any type		1	1	1	1	1	1	1	1	1	1	1	1	1	1	1	1	1	1	1	1	1	1	1	1	1	1	1	1	1	1	1	1	1	1
BJ9 vs CBJ113		1	1	1	0	1	1	1	1	1	1	1	1	1	1	1	1	1	1	1	1	1	1	1	1	1	1	1	1	1	1	1	1	1	1
BJ9 any type not CBJ113		0	0	0	1	0	0	0	0	0	0	0	0	0	0	0	0	0	0	0	0	0	0	0	0	0	0	0	0	0	0	0	0	0	0
BJ9 any type and CBJ113		0	0	1	0	1	1	1	1	1	1	1	1	1	1	1	1	1	1	1	1	1	1	1	1	1	1	1	1	1	1	1	1	1	1

Note: The ORFs core protein

Sequence category	ORF	e glycoprotein E3 gp19K																														
	position in ORF	63	75	76	77	86	87	88	90	92	94	118	120	128	131	135	136	137	145	147	155	1	2	3	4	5	6	7	8	9	10	11
1	AF534906	K	P	N	N	N	D	S	V	L	E	K	V	F	L	V	I	T	I	F	E	M	C	Q	V	G	K	A	R	H	T	P
2	NC 001405	K	T	N	D	E	N	R	T	M	K	K	L	S	L	A	L	A	L	Y	D	-	-	-	-	-	-	-	-	-	-	-
5	AC 000008	K	T	T	E	K	S	H	T	M	T	N	V	C	M	V	L	A	I	Y	E	-	-	-	-	-	-	-	-	-	-	-
6	FJ349096	K	T	N	D	E	N	R	T	M	K	K	L	S	L	A	L	A	L	Y	D	-	-	-	-	-	-	-	-	-	-	-
57	HQ003817	K	T	N	D	E	N	H	T	M	K	K	L	S	L	A	L	A	L	Y	D	-	-	-	-	-	-	-	-	-	-	-
C	KR699642	K	T	N	D	E	N	R	T	M	K	K	L	S	L	A	L	A	L	Y	D	-	-	-	-	-	-	-	-	-	-	-
4	BJ04	R	T	N	D	E	N	R	T	M	K	K	L	S	L	A	L	A	L	Y	D	-	-	-	-	-	-	-	-	-	-	-
9	BJ09	K	T	N	D	E	N	R	T	M	K	K	L	S	L	A	L	A	L	Y	D	-	-	-	-	-	-	-	-	-	-	-
BJ4 type 2 only		0	0	0	0	0	0	0	0	0	0	0	0	0	0	0	0	0	0	0	0	0	0	0	0	0	0	0	0	0	0	0
BJ4 not type2		0	0	0	0	0	0	0	0	0	0	0	0	0	0	0	0	0	0	0	0	0	0	0	0	0	0	0	0	0	0	0
BJ9 any type		1	1	1	1	1	1	1	1	1	1	1	1	1	1	1	1	1	1	1	1	1	1	1	1	1	1	1	1	1	1	1
BJ9 vs CBJ113		1	1	1	1	1	1	1	1	1	1	1	1	1	1	1	1	1	1	1	1	1	1	1	1	1	1	1	1	1	1	1
BJ9 any type not CBJ113		0	0	0	0	0	0	0	0	0	0	0	0	0	0	0	0	0	0	0	0	0	0	0	0	0	0	0	0	0	0	0
BJ9 any type and CBJ113		0	1	1	1	1	1	1	1	1	1	1	1	1	1	1	1	1	1	1	1	0	0	0	0	0	0	0	0	0	0	0

Note: The ORFs core protein

Sequence category	ORF																																Membrane glycoprotein E3 C
	position in ORF	12	13	14	15	16	18	19	20	21	22	23	24	26	27	28	29	30	31	32	33	35	36	37	41	43	44	45	46	47	48	49	50	51
1	AF534906	T	T	L	K	S	F	P	G	L	R	A	P	F	N	S	T	C	P	V	E	I	Q	-	Q	A	F	D	M	V	D	T	V	N
2	NC 001405	-	-	-	M	T	S	T	I	A	P	T	T	Y	R	N	T	-	-	-	-	-	-	-	-	-	-	-	-	-	-	-	-	-
5	AC 000008	-	-	-	-	-	-	-	-	M	S	A	S	F	G	Q	H	L	S	R	G	V	P	V	R	T	L	T	E	M	T	N	T	T
6	FJ349096	-	-	-	M	T	S	T	I	A	P	T	T	Y	R	N	T	-	-	-	-	-	-	-	-	-	-	-	-	-	-	-	-	-
57	HQ003817	-	-	-	M	T	S	T	I	A	P	T	T	Y	R	N	T	-	-	-	-	-	-	-	-	-	-	-	-	-	-	-	-	-
C	KR699642	-	-	-	-	-	-	-	-	-	-	-	-	-	-	-	-	-	-	-	-	-	-	-	-	-	-	-	-	-	-	-	-	-
4	BJ04	-	-	-	M	T	S	T	I	A	P	T	T	Y	R	N	T	-	-	-	-	-	-	-	-	-	-	-	-	-	-	-	-	-
9	BJ09	-	-	-	-	-	-	-	-	-	-	-	-	-	-	-	-	-	-	-	-	-	-	-	-	-	-	-	-	-	-	-	-	-
BJ4 type 2 only		0	0	0	0	0	0	0	0	0	0	0	0	0	0	0	0	0	0	0	0	0	0	0	0	0	0	0	0	0	0	0	0	0
BJ4 not type2		0	0	0	0	0	0	0	0	0	0	0	0	0	0	0	0	0	0	0	0	0	0	0	0	0	0	0	0	0	0	0	0	0
BJ9 any type		1	1	1	1	1	1	1	1	0	0	0	0	0	0	0	0	1	1	1	1	1	1	1	1	1	1	1	1	1	1	1	1	1
BJ9 vs CBJ113		1	1	1	1	1	1	1	1	1	1	1	1	1	1	1	1	1	1	1	1	1	1	1	1	1	1	1	1	1	1	1	1	1
BJ9 any type not CBJ113		0	0	0	0	0	0	0	0	0	0	0	0	0	0	0	0	0	0	0	0	0	0	0	0	0	0	0	0	0	0	0	0	0
BJ9 any type and CBJ113		0	0	0	0	0	0	0	0	0	0	0	0	0	0	0	0	0	0	0	0	0	0	0	0	0	0	0	0	0	0	0	0	0

Note: The ORFs core protein

Sequence category	ORF	CR1-beta																											
	position in ORF	52	53	54	55	60	62	63	64	65	69	70	74	76	77	79	98	104	112	118	120	122	123	131	132	133	134	135	136
1	AF534906	S	Y	N	T	T	T	Q	D	M	S	T	N	A	N	G	S	K	K	N	N	D	G	N	T	C	S	F	S
2	NC 001405	-	-	-	T	T	A	L	N	L	H	A	D	A	S	D	I	R	R	N	H	E	K	K	P	C	S	L	L
5	AC 000008	N	A	A	A	T	T	T	N	T	S	A	N	D	N	G	I	K	S	H	N	D	G	K	H	M	F	F	S
6	FJ349096	-	-	-	T	K	A	L	N	L	H	A	D	A	S	G	I	R	R	N	H	E	K	K	P	C	S	L	L
57	HQ003817	-	-	-	T	K	A	L	N	L	H	A	D	A	S	G	I	R	R	N	H	E	K	K	P	C	S	L	L
C	KR699642	-	-	-	-	-	-	-	-	-	-	-	-	-	-	-	I	R	R	N	H	E	K	K	P	C	S	L	L
4	BJ04	-	-	-	T	T	A	L	N	L	H	A	D	A	S	D	I	R	R	N	H	E	K	K	P	C	S	L	L
9	BJ09	-	-	-	-	-	-	-	-	-	-	-	-	-	-	-	I	R	R	N	H	E	K	K	P	C	S	L	L
BJ4 type 2 only		0	0	0	0	0	0	0	0	0	0	0	0	0	0	1	0	0	0	0	0	0	0	0	0	0	0	0	0
BJ4 not type2		0	0	0	0	0	0	0	0	0	0	0	0	0	0	0	0	0	0	0	0	0	0	0	0	0	0	0	0
BJ9 any type		1	1	1	0	0	0	0	0	0	0	0	0	0	0	0	1	1	1	1	1	1	1	1	1	1	1	1	1
BJ9 vs CBJ113		1	1	1	1	1	1	1	1	1	1	1	1	1	1	1	1	1	1	1	1	1	1	1	1	1	1	1	1
BJ9 any type not CBJ113		0	0	0	0	0	0	0	0	0	0	0	0	0	0	0	0	0	0	0	0	0	0	0	0	0	0	0	0
BJ9 any type and CBJ113		0	0	0	0	0	0	0	0	0	0	0	0	0	0	0	1	1	1	1	1	1	1	1	1	1	1	1	1

Note: The ORFs core protein

**Sequence category**	**ORF**				**Membrane protein E3 RID-alpha**																															
	position in ORF	137	138	139	6	12	25	26	34	47	53	62	66	70	82	83	86	3	4	5	6	12	13	14	16	17	18	21	24	25	27	30	36	45
1	AF534906	F	A	V	F	I	A	N	A	T	I	C	V	I	T	A	E	I	T	W	I	F	Y	T	T	V	V	Q	K	P	Q	V	R	T
2	NC 001405	L	Q	Y	L	V	A	H	P	I	V	F	V	I	R	T	D	R	S	V	I	F	C	A	P	V	L	Q	A	P	K	I	Q	N
5	AC 000008	L	T	V	F	V	S	H	A	T	I	C	V	F	R	T	E	F	T	V	T	I	C	T	S	A	F	P	-	K	Q	I	R	N
6	FJ349096	L	Q	Y	L	V	A	H	P	I	V	F	I	I	R	T	D	R	I	V	T	F	C	A	P	V	L	Q	A	P	K	I	Q	N
57	HQ003817	L	Q	Y	L	V	A	H	P	I	V	F	I	I	R	T	D	R	I	V	T	F	C	A	P	V	L	Q	A	P	K	I	Q	N
C	KR699642	L	Q	Y	L	V	A	H	P	I	V	F	V	I	R	T	D	R	S	V	I	F	C	A	P	V	L	Q	A	P	K	I	Q	N
4	BJ04	L	Q	Y	L	V	A	H	P	I	V	F	V	I	R	T	D	R	S	V	I	F	C	A	P	V	L	Q	A	P	K	I	Q	N
9	BJ09	L	Q	Y	L	V	A	H	P	I	V	F	V	I	R	T	D	R	S	V	I	F	C	A	P	V	L	Q	A	P	K	I	Q	N
BJ4 type 2 only		0	0	0	0	0	0	0	0	0	0	0	0	0	0	0	0	0	1	0	0	0	0	0	0	0	0	0	0	0	0	0	0	0
BJ4 not type2		0	0	0	0	0	0	0	0	0	0	0	0	0	0	0	0	0	0	0	0	0	0	0	0	0	0	0	0	0	0	0	0	0
BJ9 any type		1	1	1	1	1	1	1	1	1	1	1	1	1	1	1	1	1	1	1	1	1	1	1	1	1	1	1	1	1	1	1	1	1
BJ9 vs CBJ113		1	1	1	1	1	1	1	1	1	1	1	1	1	1	1	1	1	1	1	1	1	1	1	1	1	1	1	1	1	1	1	1	1
BJ9 any type not CBJ113		0	0	0	0	0	0	0	0	0	0	0	0	0	0	0	0	0	0	0	0	0	0	0	0	0	0	0	0	0	0	0	0	0
BJ9 any type and CBJ113		1	1	1	1	1	1	1	1	1	1	1	1	1	1	1	1	1	1	1	1	1	1	1	1	1	1	1	1	1	1	1	1	1

Note: The ORFs core protein

Sequence category	ORF	Membrane protein E3 RID-beta																													
	position in ORF	46	47	53	62	69	82	83	88	90	94	96	97	98	99	100	102	103	104	108	111	114	117	3	4	9	13	20	23	26	28	29
1	AF534906	E	K	T	F	I	N	A	S	S	A	A	I	I	Q	M	P	I	V	I	A	H	P	D	G	M	I	Q	K	A	Q	E
2	NC 001405	K	Q	A	M	F	N	A	N	P	V	A	V	I	P	L	Q	V	I	-	I	P	P	E	S	L	N	E	K	S	R	E
5	AC 000008	E	K	A	M	L	K	R	N	P	A	A	M	L	P	L	Q	V	V	F	A	P	T	D	T	M	I	E	R	A	Q	Q
6	FJ349096	K	Q	A	M	F	N	A	N	P	A	N	V	I	P	L	Q	V	I	-	I	P	P	E	S	L	N	E	K	S	R	E
57	HQ003817	K	Q	A	M	F	N	A	N	P	A	N	V	I	P	L	Q	V	I	-	I	P	P	E	S	L	N	E	K	S	R	E
C	KR699642	K	Q	A	M	F	N	A	N	P	V	A	V	I	P	L	Q	V	I	-	I	P	P	E	S	L	N	E	K	S	R	E
4	BJ04	K	Q	A	M	F	N	A	N	P	V	A	V	I	P	L	Q	V	I	-	I	P	P	E	S	L	N	E	K	S	R	E
9	BJ09	K	Q	A	M	F	N	A	N	P	V	A	V	I	P	L	Q	V	I	-	I	P	P	E	S	L	N	E	K	S	R	E
BJ4 type 2 only		0	0	0	0	0	0	0	0	0	1	0	0	0	0	0	0	0	0	0	0	0	0	0	0	0	0	0	0	0	0	0
BJ4 not type2		0	0	0	0	0	0	0	0	0	0	0	0	0	0	0	0	0	0	0	0	0	0	0	0	0	0	0	0	0	0	0
BJ9 any type		1	1	1	1	1	1	1	1	1	1	1	1	1	1	1	1	1	1	1	1	1	1	1	1	1	1	1	1	1	1	1
BJ9 vs CBJ113		1	1	1	1	1	1	1	1	1	1	1	1	1	1	1	1	1	1	1	1	1	1	1	1	1	1	1	1	1	1	1
BJ9 any type not CBJ113		0	0	0	0	0	0	0	0	0	0	0	0	0	0	0	0	0	0	0	0	0	0	0	0	0	0	0	0	0	0	0
BJ9 any type and CBJ113		1	1	1	1	1	1	1	1	1	1	1	1	1	1	1	1	1	1	0	1	1	1	1	1	1	1	1	1	1	1	1

Note: The ORFs core protein

Sequence category	ORF	Control protein E3 14.7K																											
	position in ORF	31	32	35	36	51	57	60	61	62	63	64	65	66	74	75	80	81	86	90	93	95	101	103	105	108	118	121	122
1	AF534906	L	N	L	Q	L	V	E	K	D	H	S	N	H	A	K	L	L	R	V	Q	V	L	F	C	P	L	L	R
2	NC 001405	L	K	V	E	V	L	E	K	T	T	T	G	N	T	Q	V	L	K	V	H	A	L	F	Y	P	M	I	R
5	AC 000008	M	N	L	Q	L	V	D	S	N	T	T	G	H	T	K	V	V	K	I	H	V	I	S	C	P	L	L	K
6	FJ349096	L	K	V	E	V	L	E	K	T	T	T	G	N	T	Q	V	L	K	V	H	A	L	F	Y	P	M	I	R
57	HQ003817	L	K	V	E	V	L	E	K	T	T	T	G	N	T	Q	V	L	K	V	H	A	L	F	Y	P	M	I	R
C	KR699642	L	K	V	E	V	L	E	K	T	T	T	G	N	T	Q	V	L	K	V	H	A	L	F	Y	S	M	I	R
4	BJ04	L	K	V	E	V	L	E	K	T	T	T	G	N	T	Q	V	L	K	V	H	A	L	F	Y	P	M	I	R
9	BJ09	L	K	V	E	V	L	E	K	T	T	T	G	N	T	Q	V	L	K	V	H	A	L	F	Y	P	M	I	R
BJ4 type 2 only		0	0	0	0	0	0	0	0	0	0	0	0	0	0	0	0	0	0	0	0	0	0	0	0	0	0	0	0
BJ4 not type2		0	0	0	0	0	0	0	0	0	0	0	0	0	0	0	0	0	0	0	0	0	0	0	0	0	0	0	0
BJ9 any type		1	1	1	1	1	1	1	1	1	1	1	1	1	1	1	1	1	1	1	1	1	1	1	1	1	1	1	1
BJ9 vs CBJ113		1	1	1	1	1	1	1	1	1	1	1	1	1	1	1	1	1	1	1	1	1	1	1	1	0	1	1	1
BJ9 any type not CBJ113		0	0	0	0	0	0	0	0	0	0	0	0	0	0	0	0	0	0	0	0	0	0	0	0	1	0	0	0
BJ9 any type and CBJ113		1	1	1	1	1	1	1	1	1	1	1	1	1	1	1	1	1	1	1	1	1	1	1	1	0	1	1	1

Sequence category	ORF			
	position in ORF	7	11	14
1	AF534906	E	Q	S
2	NC 001405	E	H	F
5	AC 000008	D	Q	T
6	FJ349096	E	H	F
57	HQ003817	E	H	F
C	KR699642	E	H	F
4	BJ04	E	H	F
9	BJ09	E	H	F
BJ4 type 2 only		0	0	0
BJ4 not type2		0	0	0
BJ9 any type		1	1	1
BJ9 vs CBJ113		1	1	1
BJ9 any type not CBJ113		0	0	0
BJ9 any type and CBJ113		1	1	1

Note: The ORFs core protein

Sequence category	ORF	Protein U																												
	position in ORF	19	27	29	31	42	48	49	52	51	56	59	68	71	74	81	82	83	87	88	98	100	101	107	108	110	111	114	116	117
1	AF534906	K	R	R	R	L	K	D	S	L	V	H	S	A	K	Q	N	I	T	E	S	E	T	V	T	G	I	V	T	A
2	NC 001405	K	K	R	R	M	K	D	T	V	D	H	S	T	K	Q	N	V	T	Q	S	D	T	I	T	G	A	V	T	T
5	AC 000008	R	R	K	Q	L	R	N	T	L	V	N	N	S	E	Q	N	V	S	P	N	E	I	V	T	E	A	V	A	A
6	FJ349096	R	K	L	K	M	K	D	T	L	V	H	S	S	Q	N	T	I	S	Q	T	E	T	V	S	G	A	M	T	T
57	HQ003817	R	K	L	K	M	K	D	T	L	V	H	S	S	Q	N	T	I	S	Q	T	E	T	V	S	G	A	M	T	T
C	KR699642	K	K	R	R	M	K	D	T	V	D	H	S	T	K	Q	N	V	T	Q	S	D	T	I	T	G	A	V	T	T
4	BJ04	K	K	R	R	M	K	D	T	V	D	H	S	T	K	Q	N	V	T	Q	S	D	T	I	T	G	A	V	T	T
9	BJ09	K	K	R	R	M	K	D	T	V	D	H	S	T	K	Q	N	V	T	Q	S	D	T	I	T	G	A	V	T	T
BJ4 type 2 only		0	0	0	0	0	0	0	0	1	1	0	0	1	0	0	0	0	0	0	0	1	0	1	0	0	0	0	0	0
BJ4 not type2		0	0	0	0	0	0	0	0	0	0	0	0	0	0	0	0	0	0	0	0	0	0	0	0	0	0	0	0	0
BJ9 any type		1	1	1	1	1	1	1	1	1	1	1	1	1	1	1	1	1	1	1	1	1	1	1	1	1	1	1	1	1
BJ9 vs CBJ113		1	1	1	1	1	1	1	1	1	1	1	1	1	1	1	1	1	1	1	1	1	1	1	1	1	1	1	1	1
BJ9 any type not CBJ113		0	0	0	0	0	0	0	0	0	0	0	0	0	0	0	0	0	0	0	0	0	0	0	0	0	0	0	0	0
BJ9 any type and CBJ113		1	1	1	1	1	1	1	1	1	1	1	1	1	1	1	1	1	1	1	1	1	1	1	1	1	1	1	1	1

Note: The ORFs core protein

Sequence category	ORF																								
	position in ORF	118	121	122	123	124	125	126	128	129	138	147	148	150	153	154	163	166	167	168	169	170	171	172	175
1	AF534906	A	V	V	A	G	N	S	T	V	Q	K	G	L	S	E	G	S	A	T	D	N	N	T	I
2	NC 001405	A	I	V	T	S	G	A	S	V	Q	K	G	I	S	D	A	S	G	S	D	S	D	T	V
5	AC 000008	A	M	V	A	G	N	T	T	M	H	Q	G	L	S	E	G	T	T	T	D	S	S	T	I
6	FJ349096	S	V	V	S	D	N	T	T	M	Q	K	E	L	L	D	A	S	A	T	D	N	N	A	I
57	HQ003817	S	V	I	S	D	N	T	T	M	Q	K	E	L	L	D	A	S	A	T	G	N	N	A	I
C	KR699642	A	I	V	T	S	G	A	S	V	Q	K	G	I	S	D	A	S	G	S	D	S	N	T	V
4	BJ04	A	I	V	T	S	G	A	S	V	Q	K	G	I	S	D	A	S	G	S	D	S	D	T	V
9	BJ09	A	I	V	T	S	G	A	S	V	Q	K	G	I	S	D	A	S	G	S	D	S	D	T	V
BJ4 type 2 only		0	1	0	1	1	1	1	1	0	0	0	0	1	0	0	0	0	1	1	0	0	1	0	1
BJ4 not type2		0	0	0	0	0	0	0	0	0	0	0	0	0	0	0	0	0	0	0	0	0	0	0	0
BJ9 any type		1	1	1	1	1	1	1	1	1	1	1	1	1	1	1	1	1	1	1	1	1	1	1	1
BJ9 vs CBJ113		1	1	1	1	1	1	1	1	1	1	1	1	1	1	1	1	1	1	1	1	1	0	1	1
BJ9 any type not CBJ113		0	0	0	0	0	0	0	0	0	0	0	0	0	0	0	0	0	0	0	0	0	1	0	0
BJ9 any type and CBJ113		1	1	1	1	1	1	1	1	1	1	1	1	1	1	1	1	1	1	1	1	1	0	1	1

Note: The ORFs core protein

Sequence category	ORF																								
	position in ORF	177	178	181	184	185	189	190	191	192	193	194	196	198	199	203	205	206	207	208	209	212	214	215	216
1	AF534906	T	S	I	T	N	G	V	N	M	E	N	L	N	N	L	L	R	V	A	G	Q	T	N	D
2	NC 001405	A	S	L	A	T	G	I	N	M	E	D	I	V	N	I	I	K	I	S	G	Q	A	Q	N
5	AC 000008	A	S	L	A	T	G	I	D	L	K	E	I	T	Q	L	L	K	Y	G	A	H	T	D	D
6	FJ349096	A	S	L	A	N	A	V	T	M	E	N	L	N	N	L	L	K	I	G	G	Q	A	T	D
57	HQ003817	T	S	L	A	N	A	V	T	M	E	N	L	N	N	L	L	K	I	G	G	Q	A	T	D
C	KR699642	A	T	L	A	T	G	I	N	M	E	D	I	V	N	I	I	K	I	S	G	Q	A	Q	N
4	BJ04	A	T	L	A	T	G	I	N	M	E	D	I	V	N	I	I	K	I	S	G	Q	A	Q	N
9	BJ09	A	T	L	A	T	G	I	N	M	E	D	I	V	N	I	I	K	I	S	G	Q	A	Q	N
BJ4 type 2 only		0	0	0	0	0	0	0	0	0	0	1	0	1	0	1	1	0	0	1	0	0	0	1	1
BJ4 not type2		0	0	0	0	0	0	0	0	0	0	0	0	0	0	0	0	0	0	0	0	0	0	0	0
BJ9 any type		1	0	1	1	1	1	1	1	1	1	1	1	1	1	1	1	1	1	1	1	1	1	1	1
BJ9 vs CBJ113		1	1	1	1	1	1	1	1	1	1	1	1	1	1	1	1	1	1	1	1	1	1	1	1
BJ9 any type not CBJ113		0	0	0	0	0	0	0	0	0	0	0	0	0	0	0	0	0	0	0	0	0	0	0	0
BJ9 any type and CBJ113		1	0	1	1	1	1	1	1	1	1	1	1	1	1	1	1	1	1	1	1	1	1	1	1

Note: The ORFs core protein

**Sequence category**	**ORF**																								
	position in ORF	217	218	219	222	223	226	229	230	231	232	233	234	236	240	243	245	247	248	249	252	253	254	255	256
1	AF534906	S	H	A	V	G	Q	A	I	D	N	N	A	H	T	I	Y	T	S	G	E	L	K	T	G
2	NC 001405	S	D	T	V	V	P	T	V	E	Q	N	S	R	A	I	Y	S	S	N	E	I	K	T	G
5	AC 000008	L	N	T	V	A	P	T	I	N	N	T	S	Q	T	L	F	S	Q	G	Q	L	N	V	A
6	FJ349096	S	H	A	L	G	Q	A	V	H	N	N	L	H	T	I	F	T	S	G	E	L	K	T	G
57	HQ003817	S	H	A	L	G	Q	A	V	H	N	N	L	H	T	I	F	T	S	G	E	L	K	T	G
C	KR699642	S	D	T	V	V	P	T	V	E	Q	N	S	R	A	I	Y	S	S	N	E	I	K	T	G
4	BJ04	S	D	T	V	V	P	T	V	E	Q	N	S	R	A	I	Y	S	S	N	E	I	K	T	G
9	BJ09	S	D	T	V	V	P	T	V	E	Q	N	S	R	A	I	Y	S	S	N	E	I	K	T	G
BJ4 type 2 only		0	1	0	0	1	0	0	0	1	1	0	0	1	1	0	0	0	0	1	0	1	0	0	0
BJ4 not type2		0	0	0	0	0	0	0	0	0	0	0	0	0	0	0	0	0	0	0	0	0	0	0	0
BJ9 any type		1	1	1	1	1	1	1	1	1	1	1	1	1	1	1	1	1	1	1	1	1	1	1	1
BJ9 vs CBJ113		1	1	1	1	1	1	1	1	1	1	1	1	1	1	1	1	1	1	1	1	1	1	1	1
BJ9 any type not CBJ113		0	0	0	0	0	0	0	0	0	0	0	0	0	0	0	0	0	0	0	0	0	0	0	0
BJ9 any type and CBJ113		1	1	1	1	1	1	1	1	1	1	1	1	1	1	1	1	1	1	1	1	1	1	1	1

Note: The ORFs core protein

Sequence category	ORF																								
	position in ORF	257	259	260	261	262	263	264	266	267	273	280	281	283	285	292	293	295	296	297	302	306	309	312	313
1	AF534906	G	V	R	V	D	S	V	R	R	D	S	Q	R	K	Y	V	S	S	T	L	K	H	T	T
2	NC 001405	G	M	R	I	N	N	-	N	L	D	T	K	R	K	Y	I	A	S	-	I	R	Y	N	A
5	AC 000008	G	L	R	I	D	S	Q	R	R	S	N	Q	N	R	F	I	S	A	-	I	K	Y	T	A
6	FJ349096	D	L	Y	V	D	S	-	-	-	-	-	-	-	-	-	-	-	-	-	-	-	-	-	A
57	HQ003817	D	L	Y	V	D	S	-	-	-	-	-	-	-	-	-	-	-	-	-	-	-	-	-	A
C	KR699642	G	M	R	I	N	N	-	N	L	D	T	K	R	K	Y	I	A	S	-	I	R	Y	N	A
4	BJ04	G	M	R	I	N	N	-	N	L	D	T	K	R	K	Y	I	A	S	-	I	R	Y	N	A
9	BJ09	G	M	R	I	N	N	-	N	L	D	T	K	R	K	Y	I	A	S	-	I	R	Y	N	A
BJ4 type 2 only		0	1	0	0	1	1	0	1	1	0	1	1	0	0	0	0	1	0	0	0	1	0	1	0
BJ4 not type2		0	0	0	0	0	0	0	0	0	0	0	0	0	0	0	0	0	0	0	0	0	0	0	0
BJ9 any type		1	1	1	1	1	1	1	1	1	1	1	1	1	1	1	1	1	1	1	1	1	1	1	1
BJ9 vs CBJ113		1	1	1	1	1	1	1	1	1	1	1	1	1	1	1	1	1	1	1	1	1	1	1	1
BJ9 any type not CBJ113		0	0	0	0	0	0	0	0	0	0	0	0	0	0	0	0	0	0	0	0	0	0	0	0
BJ9 any type and CBJ113		1	1	1	1	1	1	0	1	1	1	1	1	1	1	1	1	1	1	0	1	1	1	1	1

Note: The ORFs core protein

**Sequence category**	**ORF**	**Fiber (capsid protein IV)**																							
	position in ORF	314	315	316	317	318	321	322	323	324	325	326	327	328	331	334	335	337	338	340	342	343	348	349	350
1	AF534906	G	N	S	-	K	E	V	N	L	K	T	T	K	I	T	D	V	A	H	A	Q	G	N	D
2	NC 001405	S	N	N	T	K	E	V	S	I	K	K	S	S	N	N	T	I	A	N	G	K	D	T	N
5	AC 000008	S	N	N	S	K	E	V	N	L	S	T	A	K	M	A	T	I	A	N	G	D	G	S	P
6	FJ349096	G	P	N	-	Q	H	I	N	L	N	T	T	K	A	N	T	I	T	N	G	K	E	T	D
57	HQ003817	G	P	N	-	Q	H	I	N	L	N	T	T	K	A	N	T	I	T	N	G	K	E	T	D
C	KR699642	S	N	N	T	K	E	V	S	I	K	K	S	S	N	N	T	I	A	N	G	K	D	T	N
4	BJ04	S	N	N	T	K	E	V	S	I	K	K	S	S	N	N	T	I	A	N	G	K	D	T	N
9	BJ09	S	N	N	T	K	E	V	S	I	K	K	S	S	N	N	T	I	A	N	G	K	D	T	N
BJ4 type 2 only		0	0	0	1	0	0	0	1	1	0	1	1	1	1	0	0	0	0	0	0	0	1	0	1
BJ4 not type2		0	0	0	0	0	0	0	0	0	0	0	0	0	0	0	0	0	0	0	0	0	0	0	0
BJ9 any type		1	1	1	1	1	1	1	1	1	1	1	1	1	1	1	1	1	1	1	1	1	1	1	1
BJ9 vs CBJ113		1	1	1	1	1	1	1	1	1	1	1	1	1	1	1	1	1	1	1	1	1	1	1	1
BJ9 any type not CBJ113		0	0	0	0	0	0	0	0	0	0	0	0	0	0	0	0	0	0	0	0	0	0	0	0
BJ9 any type and CBJ113		1	1	1	1	1	1	1	1	1	1	1	1	1	1	1	1	1	1	1	1	1	1	1	1

Note: The ORFs core protein

Sequence category	ORF																								
	position in ORF	351	354	355	356	357	360	364	366	368	369	370	371	372	374	375	377	378	382	388	389	394	396	397	398
1	AF534906	T	S	T	N	T	L	L	L	L	D	Y	D	S	G	G	I	P	T	T	T	V	N	K	S
2	NC 001405	T	S	P	D	I	I	I	S	I	D	Y	N	E	G	A	I	T	A	N	S	I	N	K	N
5	AC 000008	N	A	P	N	T	L	I	H	L	E	F	D	S	K	A	V	P	T	S	T	V	N	K	N
6	FJ349096	-	S	S	N	G	I	I	S	I	Q	Y	N	T	G	A	V	A	T	S	S	M	S	I	N
57	HQ003817	-	S	S	N	G	I	I	S	I	Q	Y	D	T	G	A	V	A	T	S	S	M	S	I	N
C	KR699642	T	S	P	D	I	I	I	S	I	D	Y	N	E	G	A	I	T	A	N	S	I	N	K	N
4	BJ04	T	S	P	D	I	I	I	S	I	D	Y	N	E	G	A	I	T	A	N	S	I	N	K	N
9	BJ09	T	S	P	D	I	I	I	S	I	D	Y	N	E	G	A	I	T	A	N	S	I	N	K	N
BJ4 type 2 only		0	0	0	1	1	0	0	0	0	0	0	0	1	0	0	0	1	1	1	0	1	0	0	0
BJ4 not type2		0	0	0	0	0	0	0	0	0	0	0	0	0	0	0	0	0	0	0	0	0	0	0	0
BJ9 any type		1	1	1	1	1	1	1	1	1	1	1	1	1	1	1	1	1	1	1	1	1	1	1	1
BJ9 vs CBJ113		1	1	1	1	1	1	1	1	1	1	1	1	1	1	1	1	1	1	1	1	1	1	1	1
BJ9 any type not CBJ113		0	0	0	0	0	0	0	0	0	0	0	0	0	0	0	0	0	0	0	0	0	0	0	0
BJ9 any type and CBJ113		1	1	1	1	1	1	1	1	1	1	1	1	1	1	1	1	1	1	1	1	1	1	1	1

Note: The ORFs core protein

Sequence category	ORF																							
	position in ORF	399	401	409	415	416	417	418	419	420	422	424	427	435	437	440	441	445	447	448	449	450	451	452	455
1	AF534906	D	K	D	Q	I	H	S	E	K	A	L	V	V	A	S	A	R	S	L	A	P	I	S	I
2	NC 001405	D	K	D	R	I	H	S	D	N	C	F	V	V	A	A	A	S	D	L	S	S	M	T	V
5	AC 000008	N	K	A	R	L	N	A	E	K	A	L	V	I	A	S	V	K	S	L	A	P	I	S	V
6	FJ349096	N	R	D	R	I	A	S	D	K	C	L	A	I	G	S	A	S	N	M	A	S	I	N	L
57	HQ003817	N	R	D	R	I	A	S	D	K	C	L	A	I	G	S	A	S	N	M	A	S	I	N	L
C	KR699642	D	K	D	R	I	H	S	D	N	C	F	V	V	A	A	A	S	D	L	S	S	M	T	V
4	BJ04	D	K	D	R	I	H	S	D	N	C	F	V	V	A	A	A	S	D	L	S	S	M	T	V
9	BJ09	D	K	D	R	I	H	S	D	N	C	F	V	V	A	A	A	S	D	L	S	S	M	T	V
BJ4 type 2 only		0	0	0	0	0	0	0	0	1	0	1	0	0	0	1	0	0	1	0	1	0	1	1	0
BJ4 not type2		0	0	0	0	0	0	0	0	0	0	0	0	0	0	0	0	0	0	0	0	0	0	0	0
BJ9 any type		1	1	1	1	1	1	1	1	1	1	1	1	1	1	1	1	1	1	1	1	1	1	1	1
BJ9 vs CBJ113		1	1	1	1	1	1	1	1	1	1	1	1	1	1	1	1	1	1	1	1	1	1	1	1
BJ9 any type not CBJ113		0	0	0	0	0	0	0	0	0	0	0	0	0	0	0	0	0	0	0	0	0	0	0	0
BJ9 any type and CBJ113		1	1	1	1	1	1	1	1	1	1	1	1	1	1	1	1	1	1	1	1	1	1	1	1

Note: The ORFs core protein

Sequence category	ORF																								
		knob																							
	position in ORF	456	458	459	460	461	462	465	466	467	471	472	473	475	477	478	479	485	487	488	490	491	492	493	496
1	AF534906	S	A	H	I	I	L	N	E	H	M	N	H	S	D	P	Q	K	D	L	N	A	T	A	N
2	NC 001405	A	V	S	I	F	L	D	Q	N	M	E	N	S	K	K	H	N	N	S	N	A	N	P	N
5	AC 000008	Q	A	H	L	I	I	D	E	N	L	N	N	F	D	P	E	N	D	L	E	G	T	A	N
6	FJ349096	S	V	N	L	V	L	D	D	N	M	S	N	S	D	K	Q	N	D	S	N	G	Q	P	Y
57	HQ003817	S	V	N	L	V	L	D	D	N	M	S	N	S	D	K	Q	N	D	S	N	G	Q	P	Y
C	KR699642	A	V	S	I	F	L	D	Q	N	M	E	N	S	K	K	H	N	N	S	N	A	N	P	N
4	BJ04	A	V	S	I	F	L	D	Q	N	M	E	N	S	K	K	H	N	N	S	N	A	N	P	N
9	BJ09	A	V	S	I	F	L	D	Q	N	M	E	N	S	K	K	H	N	N	S	N	A	N	P	N
BJ4 type 2 only		1	0	1	0	1	0	0	1	0	0	1	0	0	1	0	1	0	1	0	0	0	1	0	0
BJ4 not type2		0	0	0	0	0	0	0	0	0	0	0	0	0	0	0	0	0	0	0	0	0	0	0	0
BJ9 any type		1	1	1	1	1	1	1	1	1	1	1	1	1	1	1	1	1	1	1	1	1	1	1	1
BJ9 vs CBJ113		1	1	1	1	1	1	1	1	1	1	1	1	1	1	1	1	1	1	1	1	1	1	1	1
BJ9 any type not CBJ113		0	0	0	0	0	0	0	0	0	0	0	0	0	0	0	0	0	0	0	0	0	0	0	0
BJ9 any type and CBJ113		1	1	1	1	1	1	1	1	1	1	1	1	1	1	1	1	1	1	1	1	1	1	1	1

Note: The ORFs core protein

Sequence category	ORF	505	510	511	512	513	517	526	528	530	533	534	535	543	545	546	547	549	550	551	553	556	559	561	563
	position in ORF	505	510	511	512	513	517	526	528	530	533	534	535	543	545	546	547	549	550	551	553	556	559	561	563
1	AF534906	K	T	Q	S	R	S	N	E	E	M	T	L	D	N	-	Q	T	P	A	T	I	S	S	P
2	NC 001405	L	T	Q	S	Q	N	H	D	T	M	I	L	S	S	T	E	S	E	V	T	M	T	S	E
5	AC 000008	S	S	H	G	K	S	N	D	T	V	T	L	Q	T	G	D	T	P	-	A	M	S	D	-
6	FJ349096	K	T	Q	S	K	S	N	D	S	L	H	F	D	-	-	-	N	Q	V	K	I	S	S	N
57	HQ003817	K	T	Q	S	K	S	N	D	S	L	H	F	D	-	-	-	N	Q	V	K	I	S	S	N
C	KR699642	L	T	Q	S	Q	N	H	D	T	M	I	L	S	S	T	E	S	E	V	T	M	T	S	E
4	BJ04	L	T	Q	S	Q	N	H	D	T	M	I	L	S	S	T	E	S	E	V	T	M	T	S	E
9	BJ09	L	T	Q	S	Q	N	H	D	T	M	I	L	S	S	T	E	S	E	V	T	M	T	S	E
BJ4 type 2 only		1	0	0	0	1	1	1	0	0	0	1	0	1	1	1	1	1	1	0	0	0	1	0	1
BJ4 not type2		0	0	0	0	0	0	0	0	0	0	0	0	0	0	0	0	0	0	0	0	0	0	0	0
BJ9 any type		1	1	1	1	1	1	1	1	1	1	1	1	1	1	1	1	1	1	1	1	1	1	1	1
BJ9 vs CBJ113		1	1	1	1	1	1	1	1	1	1	1	1	1	1	1	1	1	1	1	1	1	1	1	1
BJ9 any type not CBJ113		0	0	0	0	0	0	0	0	0	0	0	0	0	0	0	0	0	0	0	0	0	0	0	0
BJ9 any type and CBJ113		1	1	1	1	1	1	1	1	1	1	1	1	1	1	1	1	1	1	1	1	1	1	1	1

Note: The ORFs core protein

Sequence category	ORF										Control protein E4orf6/7					Control protein E4 34K				Control protein E4orf4		
	position in ORF	565	566	567	569	570	571	572	576	584	66	68	69	84	88	61	90	91	112	38	58	67
1	AF534906	N	Q	T	I	G	Q	T	N	A	P	E	Q	N	R	Y	L	V	M	H	I	A
2	NC 001405	G	K	-	T	T	E	T	N	A	P	E	Q	D	S	Y	L	V	M	H	I	A
5	AC 000008	G	H	N	I	N	E	I	S	A	P	K	Q	D	S	Y	L	V	M	H	I	S
6	FJ349096	G	Q	-	T	N	D	K	N	A	P	E	Q	D	S	Y	L	V	M	H	I	A
57	HQ003817	G	Q	-	T	N	D	K	N	S	P	E	Q	D	S	Y	L	I	V	H	M	A
C	KR699642	G	K	-	T	T	E	T	N	A	P	K	Q	D	S	Y	L	V	M	H	I	S
4	BJ04	G	K	-	T	T	E	T	N	A	S	E	R	D	S	F	I	I	V	R	M	A
9	BJ09	G	K	-	T	T	E	T	N	A	P	K	Q	D	S	Y	L	V	M	H	I	S
BJ4 type 2 only		0	1	0	0	1	0	0	0	0	0	0	0	0	0	0	0	0	0	0	0	0
BJ4 not type2		0	0	0	0	0	0	0	0	0	0	0	0	0	0	0	0	1	1	0	1	0
BJ9 any type		1	1	1	1	1	1	1	1	1	1	1	1	1	1	1	1	1	1	1	1	1
BJ9 vs CBJ113		1	1	1	1	1	1	1	1	1	1	1	1	1	1	1	1	1	1	1	1	1
BJ9 any type not CBJ113		0	0	0	0	0	0	0	0	0	0	0	0	0	0	0	0	0	0	0	0	0
BJ9 any type and CBJ113		1	1	0	1	1	1	1	1	1	0	1	0	1	1	0	0	1	1	0	1	1

Note: The ORFs core protein

Sequence category	ORF	Control protein E4orf3		Control protein E4orf2			Total
	position in ORF	33	70	30	48	86	
1	AF534906	I	V	A	S	A	
2	NC 001405	I	V	A	S	A	
5	AC 000008	R	V	S	S	A	
6	FJ349096	I	V	A	S	A	
57	HQ003817	I	V	A	S	V	
C	KR699642	R	V	A	L	A	
4	BJ04	R	I	A	S	A	
9	BJ09	R	V	A	S	A	
BJ4 type 2 only		0	0	0	0	0	147
BJ4 not type2		1	0	0	0	0	29
BJ9 any type		1	1	1	1	1	
BJ9 vs CBJ113		1	1	1	0	1	
BJ9 any type not CBJ113		0	0	0	1	0	30
BJ9 any type and CBJ113		1	0	1	0	1	775

Note: The ORFs core protein

Supplementary Table S6. Primers used for PCR amplification of the complete genome.

Primer	Sequences (5'-3' orientation)	Positon	GB reference strain/ accession No.
HAdV-0001S	GGGGCATCATCATAATATACCT	1--18nt	HAdV-1/AC_000017
HAdV-3482A	GCAGGCCAGCACCAAGTGATC	3462-3482nt	HAdV-1/AC_000017
HAdV-3177S	GGCTTGAGCACAACATACTGA	3177-3197nt	HAdV-1/AC_000017
HAdV-6080A	GACAGCGATGCGGAAGAGAGT	6060-6080nt	HAdV-1/AC_000017
HAdV-5968S	TCAAGGAAGGTGATTGGTTTA	5968-5988nt	HAdV-1/AC_000017
HAdV-10458A	GGTACATCTACGGATATCATC	10438-10458nt	HAdV-1/AC_000017
HAdV-10194S	TGGTATGCGCCCGTGTTGAT	10194-10213nt	HAdV-1/AC_000017
HAdV-14758A	TGCCTGCCCACTTTCAAGTAG	14738-14758nt	HAdV-1/AC_000017
HAdV-15784S	GAGTCCAGCGAGTGACCATTAC	15784-15805nt	HAdV-1/AC_000017
HAdV-19212A	AGCTAGAGCGTTGTAGGCAG	19193-19212nt	HAdV-2/J01917
HAdV-21504S	CCCATTCTCCAGTAACTTTA	21504-21523nt	HAdV-2/J01917
HAdV-26646A	GCAAGCAACTATGGCGTACTT	26626-26646nt	HAdV-2/J01917
HAdV-26456S	TTCCCAGCATCGCTACAACC	26456-26475nt	HAdV-2/J01917
HAdV-31503A	GGCTAGCTTTCCATCTGTCAC	31483-31503nt	HAdV-2/J01917
HAdV-31413S	CGTACAGTCACAAGCCCCACT	31413-31433nt	HAdV-2/J01917
HAdV-35937A	GGGGCATCATCATAATATACCT	35920-35937nt	HAdV-2/J01917

Supplementary Table S7: List of GB sequences of human adenovirus C used in the analysis

GenBank ID	Annotation	Complete name	PHF nomenclature	Country	Year	35 sequences dataset [1]	20 sequences dataset [2]	Other GenBank ID	Reference
AF534906	reference type 1			USA	1953	yes	yes	AC_000017	Lauer et al., JGenVirol. 2004
FJ349096	reference type 6			USA	1953	yes	yes		Walsh et al. JClinMicrobiol., 2011
AC_000008	reference type 5			USA	1953	yes	yes		Chroboczek et al., Virology 1992
NC_001405	reference type 2			USA	1953	yes	yes	AC_000007; J01917	Roberts et al. 1986
HQ413315	strain Tonsil 99			USA	1953	yes			Weaver et al., Virology, 2011
LC068712.1	strain: 870550			Japan	1987	yes	yes		Iida et al., unpublished
LC068713.1	strain: 870550			Japan	1987	yes			Iida et al., unpublished
KF268127.1		human/USA/CL_42/1988/5	P5H5F5	USA	1988	yes			Madupu et al., unpublished
KF429744.1		human/USA/Pitts_00107/1988/1	P1H1F1	USA	1988	no (gaps)			Madupu et al., unpublished
KF429754.1		human/USA/Pitts_00149/1990/5-F268310	P5H5F5	USA	1990	yes			Madupu et al., unpublished
KF268310.1		human/USA/Pitts_00109/1992/2	P2H2F2	USA	1992	yes	yes		Madupu et al., unpublished
LC068714.1	strain: 930113			Japan	1993	yes			Iida et al., unpublished
LC068715.1	strain: 940162			Japan	1994	yes			Iida et al., unpublished
JX173078.1		human/ARG/A15812/2000/1	P1H1F1	Argentina	2000	yes	yes		Madupu et al., unpublished
HQ003817.1	reference type 57	human/RUS/16700/2001/57	P1H57F6	Russia	2001	yes	yes		Walsh et al. JClinMicrobiol., 2011
JX173081.1		human/EGY/E53/2001/2	P2H2F2	Egypt	2001	yes			Madupu et al., unpublished
JX173080.1		human/EGY/E13/2001/1	P1H1F1	Egypt	2001	yes			Madupu et al., unpublished
JX173079.1		human/ARG/A15932/2002/2	P2H2F2	Argentina	2002	yes	yes		Madupu et al., unpublished
KX384959.1	strain T215/Ft Jackson South Carolina USA/2002			USA	2002	yes			Hang et al. JMedVirol. 2017
JX173082.1		human/USA/VT384/2003/1	P1H1F1	USA	2003	yes			Madupu et al., unpublished
JX173083.1		human/USA/VT2672/2003/1	P1H1F1	USA	2003	yes	yes		Madupu et al., unpublished
JX173085.1		human/USA/VT2612/2003/1	P1H1F1	USA	2003	yes			Madupu et al., unpublished
LC068716.1	strain: 1030787			Japan	2003	yes	yes		Iida et al., unpublished
JX173084.1		human/USA/VT5544/2003/2	P2H2F2	USA	2003	yes			Madupu et al., unpublished
LC068718.1	strain: 1040502			Japan	2004	yes	yes		Iida et al., unpublished
JX173086.1		human/USA/VT13862/2004/1	P1H1F1	USA	2004	yes	yes		Madupu et al., unpublished
KF268130.1		human/USA/UFL_Adv2/2004/2	P2H2F2	USA	2004	yes	yes		Madupu et al., unpublished
LC068717.1	strain: 1040264			Japan	2004	yes			Iida et al., unpublished
KF268129.1		human/USA/UFL_Adv6/2005/6	P6H6F6	USA	2005	yes	yes		Madupu et al., unpublished
JX173077.1		human/ARG/A8649/2005/2	P2H2F2	Argentina	2005	yes			Madupu et al., unpublished
KF268331.1		human/USA/UFL_Adv1/2005/1	P1H1F1	USA	2005	no (gaps)			Madupu et al., unpublished
LC068719.1	strain: 1050156			Japan	2005	yes			Iida et al., unpublished
LC068720.1	strain: 1050158			Japan	2005	yes			Iida et al., unpublished
JX423389.1		human/USA/ak31_AdV6/2007/6	P6H6F6	USA	2007	yes			Madupu et al., unpublished
KF268199.1		human/USA/UFL_Adv5/2008/5	P2/H5/F5	USA	2008	yes	yes		Madupu et al., unpublished
KR699642	strain CBJ113			China	2009	yes	yes		Wang et al., Sc.Rep. 2016
KF951595.1	strain DD28			China	2013	yes	yes		An et al., unpublished
AY339865						no (frameshift)			Sugarman et al., unpublished
AY601635	strain NHRC Ad5FS 7151			USA		no (frameshift)			Tibbetts et al., unpublished

1: The reason for not including the sequence in the analysis is given in parenthesis.
2: Dataset used to generate the trees in Figure 1

		1	2	3	4	5	6	7	9	10	11	12	13	14	15	16	17	18	19	20	21	22	23	24	25	26	27	28	30	31	32	33	34	35	36	37	45
1	AF534906_1953																																				
2	NC_001405_1953	0\046																																			
3	M73260_1953	0\051	0\050																																		
4	FJ349096_1953	0\044	0\022	0\049																																	
5	HQ413315_1953	0\044	0\022	0\049	0\000																																
6	LC068712vl_1987	0\044	0\024	0\049	0\004	0\005																															
7	LC068713vl_1987	0\044	0\024	0\049	0\004	0\004	0\001																														
9	KF268127vl_1988	0\050	0\050	0\001	0\048	0\049	0\048	0\048																													
10	KF429754vl_1990	0\050	0\050	0\001	0\048	0\049	0\048	0\048	0\000																												
11	KF268310vl_1992	0\045	0\002	0\049	0\022	0\022	0\023	0\023	0\049	0\049																											
12	LC068714vl_1993	0\044	0\024	0\049	0\004	0\005	0\001	0\001	0\048	0\048	0\023																										
13	LC068715vl_1994	0\045	0\024	0\049	0\004	0\004	0\001	0\001	0\049	0\049	0\023	0\001																									
14	JX173078vl_2000	0\004	0\045	0\050	0\043	0\043	0\043	0\043	0\050	0\050	0\045	0\044	0\044																								
15	JX173080vl_2001	0\020	0\033	0\051	0\031	0\031	0\031	0\032	0\051	0\051	0\033	0\032	0\032	0\020																							
16	JX173081vl_2001	0\048	0\015	0\054	0\031	0\031	0\031	0\032	0\054	0\054	0\015	0\032	0\032	0\048	0\033																						
17	HQ003817vl_2001	0\040	0\029	0\050	0\019	0\019	0\018	0\018	0\050	0\050	0\029	0\018	0\018	0\040	0\028	0\034																					
18	JX173079vl_2002	0\044	0\009	0\051	0\024	0\024	0\025	0\025	0\051	0\051	0\009	0\025	0\025	0\044	0\032	0\015	0\029																				
19	KX384959vl_2002	0\046	0\008	0\051	0\024	0\024	0\024	0\024	0\050	0\050	0\008	0\024	0\024	0\045	0\033	0\016	0\031	0\004																			
20	JX173085vl_2003	0\004	0\045	0\050	0\043	0\043	0\043	0\043	0\049	0\049	0\044	0\044	0\044	0\004	0\019	0\047	0\040	0\044	0\044																		
21	JX173083vl_2003	0\004	0\044	0\049	0\042	0\042	0\042	0\043	0\049	0\049	0\044	0\043	0\043	0\004	0\019	0\047	0\040	0\044	0\044	0\001																	
22	JX173082vl_2003	0\002	0\046	0\051	0\044	0\044	0\044	0\044	0\050	0\050	0\046	0\045	0\045	0\004	0\020	0\048	0\041	0\045	0\046	0\004	0\004																
23	JX173084vl_2003	0\046	0\004	0\050	0\024	0\024	0\025	0\026	0\050	0\050	0\004	0\026	0\026	0\046	0\034	0\016	0\030	0\010	0\010	0\045	0\045	0\046															
24	LC068716vl_2003	0\045	0\025	0\049	0\005	0\005	0\002	0\002	0\049	0\049	0\024	0\002	0\002	0\044	0\032	0\032	0\019	0\025	0\025	0\044	0\043	0\045	0\026														
25	JX173086vl_2004	0\004	0\045	0\050	0\043	0\043	0\043	0\043	0\049	0\049	0\044	0\043	0\044	0\004	0\019	0\047	0\040	0\044	0\044	0\000	0\001	0\004	0\045	0\044													
26	KF268130vl_2004	0\046	0\002	0\049	0\021	0\022	0\023	0\023	0\049	0\049	0\001	0\023	0\023	0\045	0\033	0\015	0\029	0\009	0\008	0\045	0\044	0\046	0\004	0\024	0\045												
27	LC068718vl_2004	0\044	0\024	0\049	0\005	0\005	0\000	0\001	0\049	0\049	0\023	0\001	0\001	0\043	0\031	0\032	0\018	0\025	0\024	0\043	0\042	0\044	0\025	0\002	0\043	0\023											
28	LC068717vl_2004	0\044	0\024	0\049	0\004	0\004	0\001	0\001	0\048	0\048	0\023	0\001	0\001	0\043	0\032	0\031	0\018	0\024	0\024	0\043	0\043	0\044	0\025	0\002	0\043	0\023	0\001										
30	JX173077vl_2005	0\045	0\002	0\049	0\021	0\022	0\023	0\023	0\049	0\049	0\000	0\023	0\023	0\045	0\033	0\015	0\029	0\009	0\008	0\044	0\044	0\045	0\004	0\024	0\044	0\001	0\023	0\023									
31	KF268129vl_2005	0\050	0\043	0\054	0\028	0\028	0\028	0\028	0\053	0\053	0\043	0\028	0\028	0\049	0\047	0\047	0\037	0\043	0\043	0\049	0\049	0\050	0\044	0\028	0\049	0\043	0\028	0\028	0\042								
32	LC068720vl_2005	0\044	0\024	0\049	0\005	0\005	0\002	0\002	0\049	0\049	0\023	0\002	0\002	0\043	0\032	0\032	0\018	0\024	0\024	0\043	0\043	0\044	0\025	0\003	0\043	0\023	0\002	0\001	0\023	0\028							
33	LC068719vl_2005	0\044	0\024	0\049	0\005	0\005	0\002	0\002	0\048	0\048	0\023	0\002	0\002	0\043	0\032	0\031	0\018	0\024	0\024	0\043	0\042	0\044	0\025	0\003	0\043	0\023	0\002	0\001	0\023	0\028	0\000						
34	JX423389vl_2007	0\044	0\026	0\049	0\006	0\006	0\004	0\003	0\048	0\048	0\025	0\004	0\003	0\043	0\031	0\031	0\019	0\025	0\026	0\043	0\043	0\044	0\026	0\004	0\043	0\025	0\004	0\004	0\025	0\028	0\004	0\004					
35	KF268199vl_2008	0\049	0\051	0\008	0\049	0\049	0\048	0\049	0\008	0\008	0\050	0\049	0\049	0\048	0\050	0\054	0\049	0\051	0\051	0\048	0\048	0\049	0\051	0\049	0\048	0\051	0\048	0\049	0\050	0\053	0\049	0\049	0\049				
36	KR699642_2009	0\043	0\008	0\051	0\023	0\024	0\023	0\024	0\051	0\051	0\007	0\024	0\024	0\042	0\031	0\016	0\029	0\010	0\009	0\042	0\041	0\043	0\010	0\025	0\042	0\007	0\024	0\024	0\007	0\044	0\024	0\024	0\026	0\051			
37	KF951595vl_2013	0\044	0\024	0\049	0\004	0\004	0\001	0\001	0\048	0\048	0\023	0\001	0\001	0\044	0\032	0\032	0\018	0\025	0\024	0\043	0\043	0\044	0\026	0\002	0\043	0\023	0\001	0\001	0\023	0\028	0\002	0\002	0\003	0\049	0\024		
45	BJ04x2012x2	0\044	0\005	0\051	0\024	0\024	0\025	0\025	0\050	0\050	0\005	0\025	0\025	0\043	0\032	0\014	0\028	0\009	0\010	0\042	0\042	0\044	0\006	0\026	0\042	0\005	0\025	0\025	0\005	0\042	0\025	0\025	0\025	0\050	0\009	0\025	
46	BJ09x2013x2	0\043	0\009	0\051	0\026	0\026	0\025	0\026	0\051	0\051	0\009	0\026	0\026	0\042	0\031	0\017	0\030	0\010	0\011	0\041	0\041	0\043	0\010	0\027	0\041	0\009	0\026	0\026	0\009	0\044	0\026	0\026	0\026	0\049	0\005	0\026	0\008

average_distance 0\031
maximum_distance 0\054

p_distance_WGS

Supplementary_Figure_S1

	1	2	3	4	5	6	7	8	10	11	12	13	14	15	16	17	18	19	20	21	22	23	24	25	26	27	28	30	31	32	33	34	35	36	37	45
1 AF534906_1953																																				
2 NC_001405_1953	0.013																																			
3 M73260_1953	0.013	0.008																																		
4 FJ349096_1953	0.013	0.002	0.008																																	
5 HQ413315_1953	0.013	0.002	0.008	0.000																																
6 LC068712.1_1987	0.013	0.002	0.008	0.001	0.002																															
7 LC068713.1_1987	0.013	0.003	0.008	0.002	0.002	0.001																														
9 KF268127.1_1988	0.013	0.008	0.000	0.008	0.008	0.008	0.008	0.011																												
10 KF429754.1_1990	0.013	0.008	0.000	0.008	0.008	0.008	0.008	0.011																												
11 KF268310.1_1992	0.013	0.002	0.008	0.002	0.002	0.001	0.002	0.011	0.008																											
12 LC068714.1_1993	0.013	0.002	0.008	0.002	0.002	0.000	0.001	0.011	0.008	0.002																										
13 LC068715.1_1994	0.013	0.002	0.008	0.002	0.002	0.000	0.002	0.011	0.008	0.002	0.000																									
14 JX173078.1_2000	0.009	0.011	0.011	0.010	0.010	0.010	0.011	0.008	0.011	0.010	0.010	0.011																								
15 JX173080.1_2001	0.007	0.012	0.012	0.012	0.012	0.012	0.012	0.008	0.011	0.011	0.012	0.012	0.010																							
16 JX173081.1_2001	0.008	0.012	0.012	0.012	0.012	0.012	0.012	0.008	0.012	0.012	0.012	0.012	0.010	0.002																						
17 HQ003817.1_2001	0.007	0.009	0.012	0.009	0.009	0.009	0.009	0.008	0.012	0.009	0.009	0.009	0.007	0.009	0.009																					
18 JX173079.1_2002	0.008	0.008	0.011	0.007	0.007	0.007	0.008	0.009	0.010	0.007	0.008	0.008	0.008	0.010	0.010	0.005																				
19 KX384959.1_2002	0.012	0.003	0.007	0.003	0.003	0.003	0.003	0.011	0.007	0.003	0.003	0.003	0.011	0.011	0.011	0.010	0.007																			
20 JX173085.1_2003	0.006	0.011	0.011	0.011	0.011	0.011	0.011	0.001	0.011	0.011	0.011	0.011	0.008	0.008	0.008	0.008	0.008	0.010																		
21 JX173083.1_2003	0.007	0.009	0.010	0.009	0.009	0.009	0.010	0.003	0.010	0.009	0.009	0.010	0.009	0.007	0.007	0.009	0.010	0.009	0.003																	
22 JX173082.1_2003	0.006	0.013	0.013	0.012	0.012	0.012	0.013	0.003	0.012	0.012	0.013	0.013	0.009	0.009	0.009	0.008	0.009	0.012	0.003	0.005																
23 JX173084.1_2003	0.014	0.010	0.009	0.009	0.010	0.009	0.010	0.012	0.009	0.010	0.010	0.010	0.011	0.014	0.015	0.012	0.011	0.010	0.012	0.013	0.013															
24 LC068716.1_2003	0.013	0.002	0.008	0.002	0.002	0.000	0.002	0.011	0.008	0.001	0.001	0.001	0.010	0.012	0.012	0.009	0.008	0.003	0.011	0.010	0.013	0.010														
25 JX173086.1_2004	0.006	0.011	0.011	0.011	0.011	0.011	0.011	0.001	0.011	0.011	0.011	0.011	0.008	0.008	0.008	0.007	0.008	0.010	0.000	0.002	0.003	0.012	0.011													
26 KF268130.1_2004	0.013	0.002	0.008	0.001	0.001	0.001	0.002	0.011	0.008	0.001	0.002	0.002	0.010	0.012	0.012	0.009	0.007	0.003	0.011	0.009	0.012	0.009	0.002	0.011												
27 LC068718.1_2004	0.013	0.002	0.008	0.002	0.002	0.000	0.001	0.011	0.008	0.001	0.000	0.001	0.010	0.012	0.012	0.009	0.007	0.003	0.011	0.009	0.012	0.010	0.001	0.011	0.001											
28 LC068717.1_2004	0.012	0.002	0.008	0.001	0.002	0.001	0.001	0.010	0.008	0.001	0.001	0.001	0.010	0.011	0.011	0.008	0.007	0.003	0.010	0.009	0.012	0.009	0.001	0.010	0.001	0.001										
30 JX173077.1_2005	0.013	0.002	0.008	0.002	0.002	0.002	0.002	0.011	0.008	0.001	0.002	0.002	0.011	0.012	0.012	0.008	0.007	0.003	0.011	0.009	0.012	0.010	0.002	0.011	0.002	0.002	0.001									
31 KF268129.1_2005	0.015	0.013	0.015	0.013	0.013	0.013	0.013	0.013	0.015	0.013	0.013	0.013	0.012	0.014	0.014	0.013	0.014	0.013	0.014	0.013	0.015	0.016	0.013	0.013	0.013	0.013	0.013	0.013								
32 LC068720.1_2005	0.012	0.002	0.008	0.001	0.002	0.001	0.001	0.011	0.008	0.001	0.001	0.001	0.010	0.011	0.012	0.008	0.007	0.003	0.011	0.009	0.012	0.010	0.001	0.010	0.001	0.001	0.000	0.001	0.013							
33 LC068719.1_2005	0.012	0.002	0.008	0.001	0.002	0.001	0.001	0.011	0.008	0.001	0.001	0.001	0.010	0.011	0.012	0.008	0.007	0.003	0.011	0.009	0.012	0.009	0.001	0.010	0.001	0.001	0.000	0.001	0.013	0.000						
34 JX423389.1_2007	0.010	0.009	0.009	0.009	0.009	0.009	0.009	0.009	0.009	0.009	0.009	0.009	0.008	0.009	0.010	0.010	0.009	0.008	0.009	0.008	0.010	0.012	0.009	0.009	0.008	0.009	0.009	0.009	0.012	0.009	0.009					
35 KF268199.1_2008	0.012	0.012	0.009	0.012	0.012	0.012	0.012	0.011	0.009	0.011	0.012	0.012	0.011	0.013	0.013	0.012	0.010	0.011	0.011	0.012	0.012	0.011	0.012	0.011	0.011	0.012	0.012	0.012	0.016	0.012	0.012	0.011				
36 KR699642_2009	0.011	0.003	0.007	0.003	0.003	0.002	0.003	0.010	0.007	0.002	0.003	0.003	0.010	0.010	0.010	0.009	0.007	0.003	0.010	0.008	0.011	0.010	0.003	0.010	0.003	0.003	0.002	0.003	0.013	0.002	0.002	0.008	0.011			
37 KF951595.1_2013	0.013	0.002	0.008	0.002	0.002	0.001	0.001	0.011	0.008	0.001	0.001	0.001	0.010	0.012	0.012	0.009	0.007	0.003	0.011	0.009	0.012	0.010	0.001	0.011	0.001	0.001	0.001	0.002	0.013	0.001	0.001	0.009	0.012	0.003		
45 BJ04-2012-2	0.007	0.009	0.010	0.009	0.009	0.009	0.010	0.005	0.010	0.009	0.009	0.010	0.004	0.008	0.008	0.006	0.006	0.009	0.004	0.006	0.006	0.010	0.010	0.004	0.009	0.009	0.009	0.009	0.011	0.009	0.009	0.007	0.010	0.009	0.009	
46 BJ09-2013-2	0.012	0.011	0.007	0.011	0.011	0.011	0.012	0.010	0.007	0.011	0.011	0.012	0.011	0.014	0.014	0.011	0.010	0.011	0.010	0.011	0.011	0.011	0.012	0.010	0.011	0.011	0.011	0.012	0.017	0.011	0.011	0.012	0.006	0.011	0.011	0.008

p distance 1-7000

	1	2	3	4	5	6	7	9	10	11	12	13	14	15	16	17	18	19	20	21	22	23	24	25	26	27	28	30	31	32	33	34	35	36	37	45
1 AF534906_1953																																				
2 NC_001405_1953	0.008																																			
3 M73260_1953	0.013	0.011																																		
4 FJ349096_1953	0.007	0.006	0.010																																	
5 HQ413315_1953	0.007	0.006	0.010	0.000																																
6 LC068712.1_1987	0.007	0.006	0.010	0.001	0.001																															
7 LC068713.1_1987	0.008	0.007	0.010	0.001	0.002	0.001																														
9 KF268127.1_1988	0.012	0.011	0.000	0.010	0.010	0.010	0.010																													
10 KF429754.1_1990	0.012	0.011	0.000	0.010	0.010	0.010	0.010	0.000																												
11 KF268310.1_1992	0.007	0.004	0.009	0.003	0.003	0.003	0.003	0.008	0.008																											
12 LC068714.1_1993	0.008	0.007	0.011	0.002	0.002	0.001	0.001	0.010	0.010	0.004																										
13 LC068715.1_1994	0.008	0.007	0.011	0.002	0.002	0.001	0.001	0.010	0.010	0.004	0.001																									
14 JX173078.1_2000	0.005	0.006	0.012	0.006	0.006	0.006	0.006	0.011	0.011	0.005	0.007	0.007																								
15 JX173080.1_2001	0.008	0.006	0.011	0.006	0.006	0.006	0.007	0.011	0.011	0.005	0.007	0.007	0.005																							
16 JX173081.1_2001	0.017	0.016	0.020	0.016	0.016	0.016	0.017	0.019	0.019	0.015	0.017	0.017	0.014	0.013																						
17 HQ003817.1_2001	0.005	0.007	0.012	0.006	0.006	0.006	0.007	0.012	0.012	0.006	0.007	0.007	0.004	0.007	0.016																					
18 JX173079.1_2002	0.007	0.004	0.011	0.006	0.006	0.006	0.006	0.010	0.010	0.004	0.006	0.006	0.005	0.006	0.016	0.007																				
19 KX384959.1_2002	0.009	0.006	0.010	0.006	0.007	0.007	0.007	0.010	0.010	0.005	0.007	0.007	0.007	0.007	0.016	0.007	0.006																			
20 JX173085.1_2003	0.006	0.004	0.010	0.005	0.005	0.005	0.005	0.009	0.009	0.003	0.005	0.005	0.004	0.005	0.015	0.005	0.003	0.005																		
21 JX173083.1_2003	0.006	0.004	0.009	0.004	0.004	0.004	0.005	0.009	0.009	0.003	0.005	0.005	0.004	0.004	0.014	0.005	0.003	0.004	0.000																	
22 JX173082.1_2003	0.002	0.008	0.013	0.008	0.008	0.008	0.008	0.013	0.013	0.007	0.009	0.009	0.005	0.008	0.017	0.006	0.008	0.009	0.006	0.006																
23 JX173084.1_2003	0.008	0.007	0.010	0.007	0.007	0.007	0.008	0.010	0.010	0.005	0.008	0.008	0.008	0.008	0.017	0.009	0.007	0.008	0.006	0.005	0.009															
24 LC068716.1_2003	0.010	0.009	0.011	0.005	0.005	0.004	0.005	0.011	0.011	0.006	0.005	0.005	0.009	0.009	0.019	0.010	0.009	0.010	0.008	0.008	0.011	0.010														
25 JX173086.1_2004	0.006	0.004	0.010	0.005	0.005	0.005	0.005	0.009	0.009	0.003	0.005	0.005	0.004	0.005	0.015	0.005	0.003	0.005	0.000	0.000	0.006	0.006	0.008													
26 KF268130.1_2004	0.008	0.005	0.010	0.003	0.003	0.003	0.004	0.010	0.010	0.002	0.004	0.004	0.006	0.007	0.016	0.007	0.005	0.006	0.004	0.004	0.008	0.006	0.007	0.004												
27 LC068718.1_2004	0.008	0.006	0.010	0.002	0.002	0.000	0.001	0.010	0.010	0.003	0.001	0.001	0.006	0.007	0.017	0.007	0.006	0.007	0.005	0.005	0.008	0.007	0.005	0.005	0.004											
28 LC068717.1_2004	0.008	0.006	0.010	0.001	0.002	0.001	0.001	0.010	0.010	0.003	0.001	0.001	0.006	0.007	0.017	0.007	0.006	0.007	0.005	0.005	0.008	0.007	0.005	0.005	0.004	0.001										
30 JX173077.1_2005	0.007	0.004	0.009	0.003	0.004	0.003	0.004	0.009	0.009	0.000	0.004	0.004	0.005	0.006	0.016	0.006	0.004	0.005	0.003	0.003	0.007	0.005	0.007	0.003	0.002	0.004	0.004									
31 KF268129.1_2005	0.021	0.020	0.020	0.019	0.020	0.019	0.020	0.020	0.020	0.018	0.020	0.020	0.020	0.020	0.024	0.020	0.019	0.019	0.018	0.018	0.022	0.020	0.019	0.018	0.019	0.020	0.019	0.018								
32 LC068720.1_2005	0.008	0.006	0.010	0.003	0.003	0.003	0.003	0.010	0.010	0.003	0.004	0.004	0.006	0.007	0.016	0.007	0.006	0.006	0.005	0.004	0.008	0.007	0.007	0.005	0.003	0.003	0.002	0.003	0.019							
33 LC068719.1_2005	0.008	0.006	0.010	0.003	0.003	0.003	0.003	0.010	0.010	0.003	0.004	0.004	0.006	0.007	0.016	0.007	0.006	0.006	0.005	0.004	0.008	0.007	0.007	0.005	0.003	0.003	0.002	0.003	0.019	0.000						
34 JX423389.1_2007	0.010	0.009	0.009	0.005	0.005	0.005	0.004	0.009	0.009	0.006	0.005	0.005	0.008	0.008	0.018	0.008	0.008	0.009	0.006	0.006	0.010	0.009	0.008	0.006	0.007	0.005	0.005	0.007	0.021	0.007	0.007					
35 KF268199.1_2008	0.011	0.009	0.010	0.009	0.009	0.009	0.009	0.010	0.010	0.008	0.010	0.010	0.009	0.008	0.017	0.010	0.008	0.008	0.008	0.007	0.011	0.011	0.011	0.008	0.009	0.009	0.009	0.009	0.018	0.010	0.010	0.010				
36 KR699642_2009	0.006	0.006	0.011	0.004	0.004	0.004	0.004	0.010	0.010	0.003	0.005	0.005	0.005	0.007	0.017	0.005	0.006	0.007	0.005	0.005	0.007	0.008	0.007	0.005	0.004	0.004	0.004	0.004	0.019	0.004	0.004	0.008	0.010			
37 KF951595.1_2013	0.008	0.007	0.010	0.001	0.002	0.001	0.001	0.010	0.010	0.003	0.001	0.001	0.006	0.007	0.017	0.007	0.006	0.007	0.005	0.005	0.008	0.008	0.005	0.005	0.004	0.001	0.001	0.004	0.020	0.003	0.003	0.005	0.010	0.004		
45 BJ04-2012-2	0.008	0.006	0.012	0.007	0.007	0.007	0.007	0.011	0.011	0.005	0.007	0.007	0.006	0.007	0.017	0.007	0.005	0.007	0.003	0.003	0.008	0.008	0.010	0.003	0.006	0.007	0.007	0.005	0.020	0.007	0.007	0.008	0.010	0.007	0.007	
46 BJ09-2013-2	0.006	0.005	0.010	0.005	0.005	0.005	0.005	0.010	0.010	0.003	0.006	0.006	0.005	0.005	0.015	0.005	0.003	0.005	0.002	0.001	0.006	0.006	0.008	0.002	0.004	0.005	0.005	0.004	0.019	0.005	0.005	0.006	0.008	0.005	0.005	0.003

pLdistanceL7001-14150

		1	2	3	4	5	6	7	9	10	11	12	13	14	15	16
1	AF534906_1953															
2	NC_001405_1953	0.008														
3	M73260_1953	0.015	0.015													
4	FJ349096_1953	0.003	0.009	0.013												
5	HQ413315_1953	0.003	0.009	0.013	0.000											
6	LC068712.1_1987	0.001	0.008	0.015	0.003	0.003										
7	LC068713.1_1987	0.003	0.008	0.013	0.001	0.001	0.003									
9	KF268127.1_1988	0.015	0.015	0.000	0.013	0.013	0.015	0.013								
10	KF429754.1_1990	0.015	0.015	0.000	0.013	0.013	0.015	0.013	0.000							
11	KF268310.1_1992	0.006	0.001	0.013	0.008	0.008	0.006	0.007	0.013	0.013						
12	LC068714.1_1993	0.004	0.009	0.015	0.002	0.002	0.004	0.001	0.015	0.015	0.008					
13	LC068715.1_1994	0.003	0.009	0.014	0.001	0.001	0.003	0.001	0.014	0.014	0.008	0.001				
14	JX173078.1_2000	0.002	0.009	0.016	0.005	0.005	0.002	0.004	0.016	0.016	0.008	0.005	0.005			
15	JX173080.1_2001	0.003	0.009	0.016	0.006	0.006	0.003	0.005	0.016	0.016	0.008	0.006	0.006	0.005		
16	JX173081.1_2001	0.027	0.029	0.028	0.026	0.026	0.026	0.026	0.028	0.028	0.028	0.028	0.027	0.028	0.028	
17	HQ003817.1_2001	0.001	0.008	0.015	0.003	0.003	0.001	0.003	0.015	0.015	0.006	0.004	0.003	0.002	0.003	0.026
18	JX173079.1_2002	0.014	0.015	0.014	0.013	0.013	0.014	0.012	0.014	0.014	0.014	0.013	0.013	0.015	0.015	0.029
19	KX384959.1_2002	0.014	0.015	0.014	0.013	0.013	0.014	0.012	0.014	0.014	0.014	0.013	0.013	0.015	0.015	0.029
20	JX173085.1_2003	0.002	0.008	0.016	0.004	0.004	0.002	0.003	0.016	0.016	0.007	0.005	0.004	0.003	0.004	0.026
21	JX173083.1_2003	0.001	0.008	0.015	0.003	0.003	0.001	0.003	0.015	0.015	0.006	0.004	0.003	0.002	0.003	0.027
22	JX173082.1_2003	0.001	0.008	0.016	0.004	0.004	0.002	0.003	0.016	0.016	0.007	0.005	0.004	0.003	0.004	0.028
23	JX173084.1_2003	0.005	0.006	0.016	0.007	0.007	0.005	0.006	0.016	0.016	0.005	0.008	0.007	0.006	0.006	0.029
24	LC068716.1_2003	0.003	0.008	0.013	0.001	0.001	0.003	0.000	0.013	0.013	0.007	0.001	0.001	0.004	0.005	0.026
25	JX173086.1_2004	0.002	0.009	0.016	0.005	0.005	0.002	0.004	0.016	0.016	0.008	0.005	0.005	0.003	0.005	0.028
26	KF268130.1_2004	0.006	0.001	0.013	0.008	0.008	0.006	0.007	0.013	0.013	0.000	0.008	0.008	0.008	0.008	0.028
27	LC068718.1_2004	0.001	0.008	0.015	0.003	0.003	0.000	0.003	0.015	0.015	0.006	0.004	0.003	0.002	0.003	0.026
28	LC068717.1_2004	0.005	0.009	0.016	0.005	0.005	0.005	0.004	0.016	0.016	0.008	0.005	0.005	0.006	0.007	0.029
30	JX173077.1_2005	0.006	0.001	0.013	0.008	0.008	0.006	0.007	0.013	0.013	0.000	0.008	0.008	0.008	0.008	0.028
31	KF268129.1_2005	0.012	0.016	0.013	0.012	0.012	0.012	0.012	0.013	0.013	0.015	0.013	0.012	0.013	0.013	0.024
32	LC068720.1_2005	0.004	0.009	0.016	0.005	0.005	0.004	0.005	0.016	0.016	0.008	0.006	0.005	0.005	0.006	0.030
33	LC068719.1_2005	0.004	0.009	0.016	0.005	0.005	0.004	0.005	0.016	0.016	0.008	0.006	0.005	0.005	0.006	0.030
34	JX423389.1_2007	0.003	0.008	0.013	0.001	0.001	0.003	0.000	0.013	0.013	0.007	0.001	0.001	0.004	0.005	0.026
35	KF268199.1_2008	0.005	0.012	0.014	0.005	0.005	0.005	0.005	0.014	0.014	0.011	0.006	0.005	0.006	0.006	0.026
36	KR699642_2009	0.002	0.008	0.016	0.005	0.005	0.002	0.004	0.016	0.016	0.006	0.005	0.005	0.003	0.005	0.028
37	KF951595.1_2013	0.004	0.009	0.015	0.002	0.002	0.004	0.001	0.015	0.015	0.008	0.002	0.002	0.005	0.006	0.028
45	BJ04-2012-2	**0.002**	0.007	0.015	0.005	0.005	**0.002**	0.004	0.015	0.015	0.006	0.005	0.005	0.003	0.005	0.027
46	BJ09-2013-2	**0.002**	0.008	0.016	0.004	0.004	**0.002**	0.003	0.016	0.016	0.007	0.005	0.004	0.003	0.004	0.028

		17	18	19	20	21	22	23	24	25	26	27	28	30	31	32	33	34	35	36	37	45
18	JX173079.1_2002	0.014																				
19	KX384959.1_2002	0.014	0.000																			
20	JX173085.1_2003	0.002	0.015	0.015																		
21	JX173083.1_2003	0.001	0.014	0.014	0.001																	
22	JX173082.1_2003	0.002	0.015	0.015	0.002	0.002																
23	JX173084.1_2003	0.005	0.016	0.016	0.005	0.005	0.005															
24	LC068716.1_2003	0.003	0.012	0.012	0.003	0.003	0.003	0.006														
25	JX173086.1_2004	0.002	0.015	0.015	0.002	0.001	0.003	0.006	0.004													
26	KF268130.1_2004	0.006	0.014	0.014	0.007	0.006	0.007	0.005	0.007	0.008												
27	LC068718.1_2004	0.001	0.014	0.014	0.002	0.001	0.002	0.005	0.003	0.002	0.006											
28	LC068717.1_2004	0.005	0.015	0.015	0.005	0.005	0.005	0.008	0.004	0.006	0.008	0.005										
30	JX173077.1_2005	0.006	0.014	0.014	0.007	0.006	0.007	0.005	0.007	0.008	0.000	0.006	0.008									
31	KF268129.1_2005	0.012	0.016	0.016	0.013	0.012	0.013	0.015	0.012	0.013	0.015	0.012	0.015	0.015								
32	LC068720.1_2005	0.004	0.016	0.016	0.005	0.004	0.005	0.007	0.005	0.005	0.008	0.004	0.002	0.008	0.015							
33	LC068719.1_2005	0.004	0.016	0.016	0.005	0.004	0.005	0.007	0.005	0.005	0.008	0.004	0.002	0.008	0.015	0.000						
34	JX423389.1_2007	0.003	0.012	0.012	0.003	0.003	0.003	0.006	0.000	0.004	0.007	0.003	0.004	0.007	0.012	0.005	0.005					
35	KF268199.1_2008	0.005	0.012	0.012	0.006	0.005	0.006	0.006	0.005	0.006	0.011	0.005	0.007	0.011	0.011	0.008	0.008	0.005				
36	KR699642_2009	0.002	0.014	0.014	0.003	0.002	0.003	0.005	0.004	0.003	0.006	0.002	0.006	0.006	0.013	0.005	0.005	0.004	0.006			
37	KF951595.1_2013	0.004	0.013	0.013	0.005	0.004	0.005	0.008	0.001	0.005	0.008	0.004	0.005	0.008	0.013	0.006	0.006	0.001	0.006	0.005		
45	BJ04-2012-2	**0.002**	0.014	0.014	0.003	**0.002**	0.003	0.005	0.004	0.003	0.006	**0.002**	0.003	0.006	0.013	0.003	0.003	0.004	0.006	0.003	0.005	
46	BJ09-2013-2	**0.002**	0.015	0.015	**0.002**	**0.002**	**0.002**	0.005	0.003	0.003	0.007	**0.002**	0.005	0.007	0.013	0.005	0.005	0.003	0.006	0.003	0.005	0.003

pLdistanceL14151-15866

	1	2	3	4	5	6	7	9	10	11	12	13	14	15	16	17	18	19	20	21	22	23	24	25	26	27	28	30	31	32	33	34	35	36	37	45
1 AF534906_1953																																				
2 NC_001405_1953	0.016																																			
3 M73260_1953	0.017	0.014																																		
4 FJ349096_1953	0.013	0.014	0.011																																	
5 HQ413315_1953	0.014	0.015	0.012	0.001																																
6 LC068712.1_1987	0.008	0.013	0.011	0.004	0.005																															
7 LC068713.1_1987	0.013	0.014	0.011	0.001	0.002	0.004																														
9 KF268127.1_1988	0.017	0.013	0.000	0.011	0.012	0.011	0.011																													
10 KF429754.1_1990	0.017	0.013	0.000	0.011	0.012	0.011	0.011	0.000																												
11 KF268310.1_1992	0.015	0.001	0.013	0.013	0.014	0.012	0.013	0.013	0.013																											
12 LC068714.1_1993	0.012	0.013	0.011	0.001	0.002	0.005	0.001	0.011	0.011	0.012																										
13 LC068715.1_1994	0.013	0.014	0.011	0.001	0.002	0.004	0.001	0.011	0.011	0.013	0.001																									
14 JX173078.1_2000	0.000	0.016	0.018	0.012	0.013	0.008	0.012	0.017	0.017	0.015	0.012	0.012																								
15 JX173080.1_2001	0.003	0.018	0.019	0.014	0.015	0.010	0.014	0.019	0.019	0.017	0.013	0.014	0.003																							
16 JX173081.1_2001	0.018	0.016	0.014	0.012	0.013	0.012	0.012	0.014	0.014	0.015	0.012	0.012	0.018	0.020																						
17 HQ003817.1_2001	0.013	0.014	0.014	0.007	0.008	0.008	0.007	0.013	0.013	0.014	0.007	0.007	0.013	0.015	0.014																					
18 JX173079.1_2002	0.011	0.013	0.016	0.011	0.012	0.014	0.011	0.015	0.015	0.012	0.011	0.011	0.011	0.013	0.017	0.014																				
19 KX384959.1_2002	0.011	0.012	0.017	0.014	0.015	0.014	0.014	0.016	0.016	0.011	0.013	0.014	0.011	0.013	0.018	0.015	0.003																			
20 JX173085.1_2003	0.002	0.016	0.018	0.012	0.013	0.008	0.012	0.017	0.017	0.015	0.011	0.012	0.002	0.004	0.018	0.013	0.011	0.010																		
21 JX173083.1_2003	0.002	0.016	0.018	0.012	0.013	0.008	0.012	0.017	0.017	0.015	0.011	0.012	0.002	0.004	0.018	0.013	0.011	0.010	0.000																	
22 JX173082.1_2003	0.000	0.016	0.017	0.013	0.014	0.008	0.013	0.017	0.017	0.015	0.012	0.013	0.000	0.003	0.018	0.013	0.011	0.011	0.002	0.002																
23 JX173084.1_2003	0.015	0.001	0.013	0.013	0.014	0.012	0.013	0.013	0.013	0.001	0.012	0.013	0.015	0.017	0.015	0.014	0.012	0.011	0.015	0.015	0.015															
24 LC068716.1_2003	0.013	0.014	0.011	0.001	0.002	0.004	0.001	0.011	0.011	0.013	0.001	0.001	0.013	0.014	0.013	0.007	0.011	0.014	0.012	0.012	0.013	0.013														
25 JX173086.1_2004	0.003	0.016	0.018	0.012	0.013	0.008	0.012	0.017	0.017	0.015	0.012	0.012	0.002	0.004	0.018	0.013	0.011	0.011	0.000	0.000	0.003	0.015	0.013													
26 KF268130.1_2004	0.015	0.002	0.013	0.013	0.014	0.013	0.013	0.012	0.012	0.001	0.013	0.013	0.016	0.018	0.016	0.014	0.012	0.012	0.015	0.015	0.015	0.001	0.014	0.016												
27 LC068718.1_2004	0.008	0.013	0.011	0.004	0.005	0.000	0.004	0.011	0.011	0.013	0.005	0.004	0.008	0.010	0.012	0.008	0.015	0.015	0.008	0.008	0.008	0.013	0.004	0.008	0.013											
28 LC068717.1_2004	0.013	0.014	0.011	0.001	0.002	0.004	0.001	0.011	0.011	0.013	0.002	0.001	0.012	0.014	0.012	0.007	0.011	0.014	0.012	0.012	0.013	0.013	0.001	0.012	0.013	0.004										
30 JX173077.1_2005	0.015	0.001	0.013	0.013	0.014	0.012	0.013	0.013	0.013	0.001	0.012	0.013	0.015	0.017	0.015	0.014	0.012	0.011	0.015	0.015	0.015	0.001	0.013	0.015	0.001	0.013	0.013									
31 KF268129.1_2005	0.018	0.019	0.017	0.014	0.016	0.014	0.014	0.017	0.017	0.019	0.014	0.014	0.018	0.020	0.020	0.016	0.017	0.018	0.018	0.018	0.018	0.019	0.015	0.018	0.018	0.015	0.014	0.019								
32 LC068720.1_2005	0.013	0.014	0.011	0.001	0.002	0.004	0.001	0.011	0.011	0.013	0.002	0.001	0.012	0.014	0.012	0.007	0.011	0.014	0.012	0.012	0.013	0.013	0.001	0.012	0.013	0.004	0.000	0.013	0.014							
33 LC068719.1_2005	0.013	0.014	0.011	0.001	0.002	0.004	0.001	0.011	0.011	0.013	0.002	0.001	0.012	0.014	0.012	0.007	0.011	0.014	0.012	0.012	0.013	0.013	0.001	0.012	0.013	0.004	0.000	0.013	0.014	0.000						
34 JX423389.1_2007	0.013	0.014	0.011	0.001	0.002	0.004	0.001	0.011	0.011	0.013	0.001	0.001	0.012	0.014	0.012	0.007	0.011	0.014	0.012	0.012	0.013	0.013	0.001	0.012	0.013	0.004	0.001	0.013	0.014	0.001	0.001					
35 KF268199.1_2008	0.008	0.015	0.013	0.008	0.009	0.003	0.008	0.012	0.012	0.014	0.008	0.008	0.007	0.009	0.013	0.009	0.015	0.015	0.008	0.008	0.008	0.014	0.008	0.008	0.014	0.003	0.007	0.014	0.017	0.007	0.007	0.008				
36 KR699642_2009	0.003	0.017	0.019	0.014	0.015	0.010	0.014	0.018	0.018	0.016	0.013	0.014	0.002	0.005	0.019	0.015	0.012	0.011	0.003	0.003	0.003	0.016	0.014	0.003	0.017	0.010	0.014	0.016	0.018	0.014	0.014	0.014	0.009			
37 KF951595.1_2013	0.014	0.015	0.012	0.002	0.003	0.005	0.002	0.012	0.012	0.014	0.002	0.002	0.013	0.015	0.013	0.008	0.012	0.015	0.013	0.013	0.014	0.014	0.002	0.013	0.014	0.005	0.002	0.014	0.015	0.002	0.002	0.002	0.009	0.015		
45 BJ04-2012-2	0.009	0.010	0.015	0.010	0.011	**0.005**	0.010	0.015	0.015	0.009	0.009	0.010	0.008	0.010	0.015	0.010	0.015	0.015	0.008	0.008	0.009	0.009	0.010	0.008	0.009	**0.005**	0.009	0.009	0.017	0.009	0.009	0.010	0.007	0.010	0.011	
46 BJ09-2013-2	0.004	0.018	0.019	0.014	0.015	0.010	0.014	0.019	0.019	0.017	0.013	0.014	0.004	0.005	0.020	0.015	0.012	0.012	**0.002**	**0.002**	0.004	0.017	0.014	0.003	0.017	0.010	0.014	0.017	0.019	0.014	0.014	0.014	0.010	0.005	0.015	0.009

pLdistanceL15867-18837

		1	2	3	4	5	6	7	9	10	11	12	13	14	15	16	17	18	19	20	21	22	23	24	25	26	27	28	30	31	32	33	34	35	36	37	45
1	AF534906_1953																																				
2	NC_001405_1953	0.138																																			
3	M73260_1953	0.155	0.158																																		
4	FJ349096_1953	0.150	0.099	0.157																																	
5	HQ413315_1953	0.151	0.100	0.158	0.001																																
6	LC068712.1_1987	0.150	0.099	0.157	0.001	0.001																															
7	LC068713.1_1987	0.150	0.100	0.157	0.001	0.002	0.000																														
9	KF268127.1_1988	0.155	0.158	0.002	0.157	0.157	0.157	0.156																													
10	KF429754.1_1990	0.155	0.158	0.002	0.157	0.157	0.157	0.156	0.000																												
11	KF268310.1_1992	0.138	0.000	0.158	0.099	0.100	0.100	0.100	0.158	0.158																											
12	LC068714.1_1993	0.151	0.099	0.155	0.002	0.003	0.002	0.002	0.155	0.155	0.100																										
13	LC068715.1_1994	0.150	0.099	0.157	0.001	0.001	0.000	0.000	0.157	0.157	0.100	0.002																									
14	JX173078.1_2000	0.000	0.138	0.155	0.150	0.151	0.150	0.150	0.155	0.155	0.138	0.151	0.150																								
15	JX173080.1_2001	0.002	0.139	0.155	0.151	0.151	0.151	0.150	0.155	0.155	0.139	0.152	0.151	0.002																							
16	JX173081.1_2001	0.140	0.022	0.160	0.102	0.103	0.102	0.103	0.160	0.160	0.022	0.104	0.102	0.140	0.141																						
17	HQ003817.1_2001	0.113	0.108	0.143	0.108	0.108	0.108	0.107	0.142	0.142	0.108	0.107	0.108	0.113	0.114	0.110																					
18	JX173079.1_2002	0.142	0.021	0.164	0.092	0.092	0.092	0.092	0.164	0.164	0.021	0.092	0.092	0.141	0.142	0.028	0.116																				
19	KX384959.1_2002	0.142	0.021	0.164	0.092	0.092	0.092	0.092	0.164	0.164	0.021	0.092	0.092	0.142	0.142	0.028	0.115	0.000																			
20	JX173085.1_2003	0.002	0.137	0.154	0.149	0.150	0.149	0.149	0.154	0.154	0.137	0.150	0.149	0.001	0.002	0.139	0.112	0.141	0.141																		
21	JX173083.1_2003	0.002	0.138	0.154	0.149	0.150	0.149	0.149	0.154	0.154	0.137	0.150	0.149	0.002	0.003	0.140	0.112	0.141	0.141	0.000																	
22	JX173082.1_2003	0.001	0.139	0.155	0.150	0.151	0.150	0.150	0.155	0.155	0.138	0.151	0.150	0.001	0.003	0.141	0.114	0.142	0.142	0.002	0.003																
23	JX173084.1_2003	0.139	0.000	0.158	0.099	0.100	0.100	0.100	0.159	0.159	0.001	0.100	0.100	0.138	0.139	0.022	0.109	0.020	0.021	0.138	0.138	0.139															
24	LC068716.1_2003	0.150	0.099	0.157	0.001	0.001	0.000	0.000	0.157	0.157	0.100	0.002	0.000	0.150	0.151	0.102	0.108	0.092	0.092	0.149	0.149	0.150	0.100														
25	JX173086.1_2004	0.002	0.137	0.154	0.150	0.150	0.150	0.149	0.154	0.154	0.137	0.151	0.150	0.002	0.003	0.139	0.112	0.141	0.141	0.000	0.001	0.003	0.137	0.150													
26	KF268130.1_2004	0.139	0.001	0.159	0.100	0.100	0.100	0.100	0.159	0.159	0.001	0.100	0.100	0.139	0.140	0.022	0.109	0.021	0.022	0.138	0.138	0.139	0.001	0.100	0.138												
27	LC068718.1_2004	0.150	0.099	0.157	0.001	0.001	0.000	0.000	0.157	0.157	0.100	0.002	0.000	0.150	0.151	0.102	0.108	0.092	0.092	0.149	0.149	0.150	0.100	0.000	0.150	0.100											
28	LC068717.1_2004	0.150	0.099	0.157	0.001	0.001	0.000	0.000	0.157	0.157	0.100	0.002	0.000	0.150	0.151	0.102	0.108	0.092	0.092	0.149	0.149	0.150	0.100	0.000	0.150	0.100	0.000										
30	JX173077.1_2005	0.138	0.000	0.158	0.099	0.100	0.099	0.100	0.158	0.158	0.000	0.099	0.099	0.138	0.139	0.022	0.108	0.021	0.021	0.137	0.138	0.139	0.000	0.099	0.137	0.001	0.099	0.099									
31	KF268129.1_2005	0.149	0.095	0.155	0.017	0.018	0.017	0.017	0.155	0.155	0.095	0.017	0.017	0.149	0.150	0.104	0.104	0.097	0.097	0.148	0.148	0.149	0.094	0.017	0.148	0.095	0.017	0.017	0.095								
32	LC068720.1_2005	0.150	0.099	0.157	0.001	0.001	0.000	0.000	0.157	0.157	0.100	0.002	0.000	0.150	0.151	0.102	0.108	0.092	0.092	0.149	0.149	0.150	0.100	0.000	0.150	0.100	0.000	0.000	0.099	0.017							
33	LC068719.1_2005	0.150	0.099	0.157	0.001	0.001	0.000	0.000	0.157	0.157	0.100	0.002	0.000	0.150	0.151	0.102	0.108	0.092	0.092	0.149	0.149	0.150	0.100	0.000	0.150	0.100	0.000	0.000	0.099	0.017	0.000						
34	JX423389.1_2007	0.150	0.099	0.157	0.001	0.001	0.000	0.000	0.157	0.157	0.100	0.002	0.000	0.150	0.151	0.102	0.108	0.092	0.092	0.149	0.149	0.150	0.100	0.000	0.150	0.100	0.000	0.000	0.099	0.017	0.000	0.000					
35	KF268199.1_2008	0.155	0.162	0.008	0.157	0.158	0.157	0.157	0.006	0.006	0.162	0.158	0.157	0.155	0.155	0.160	0.147	0.167	0.167	0.154	0.154	0.155	0.162	0.157	0.154	0.163	0.157	0.157	0.162	0.157	0.157	0.157	0.157				
36	KR699642_2009	0.136	0.012	0.163	0.099	0.099	0.099	0.099	0.164	0.164	0.012	0.100	0.099	0.136	0.137	0.023	0.110	0.023	0.023	0.135	0.135	0.136	0.012	0.099	0.135	0.012	0.099	0.099	0.012	0.099	0.099	0.099	0.099	0.164			
37	KF951595.1_2013	0.150	0.099	0.157	0.001	0.001	0.000	0.000	0.157	0.157	0.100	0.002	0.000	0.150	0.151	0.102	0.108	0.092	0.092	0.149	0.149	0.150	0.100	0.000	0.150	0.100	0.000	0.000	0.099	0.017	0.000	0.000	0.000	0.157	0.099		
45	BJ04-2012-2	0.139	0.001	0.159	0.100	0.100	0.100	0.100	0.159	0.159	0.001	0.100	0.100	0.139	0.140	0.022	0.109	0.021	0.022	0.138	0.138	0.139	0.001	0.100	0.138	0.001	0.100	0.100	0.001	0.095	0.100	0.100	0.100	0.163	0.012	0.100	
46	BJ09-2013-2	0.136	0.011	0.163	0.099	0.099	0.099	0.099	0.164	0.164	0.012	0.100	0.099	0.135	0.136	0.023	0.109	0.023	0.023	0.135	0.135	0.136	0.012	0.099	0.135	0.012	0.099	0.099	0.011	0.098	0.099	0.099	0.099	0.164	0.000	0.099	0.011

pLdistanceL18838-21744

		1	2	3	4	5	6	7	9	10	11	12	13	14	15	16	17	18	19	20	21	22	23	24	25	26	27	28	30	31	32	33	34	35	36	37	45
1	AF534906_1953																																				
2	NC_001405_1953	0.020																																			
3	M73260_1953	0.030	0.023																																		
4	FJ349096_1953	0.018	0.004	0.026																																	
5	HQ413315_1953	0.018	0.004	0.026	0.000																																
6	LC068712.1_1987	0.023	0.017	0.025	0.015	0.015																															
7	LC068713.1_1987	0.022	0.016	0.025	0.014	0.014	0.001																														
9	KF268127.1_1988	0.030	0.023	0.000	0.026	0.026	0.025	0.025																													
10	KF429754.1_1990	0.030	0.023	0.000	0.026	0.026	0.025	0.025	0.000																												
11	KF268310.1_1992	0.020	0.001	0.023	0.005	0.005	0.017	0.017	0.023	0.023																											
12	LC068714.1_1993	0.023	0.017	0.025	0.015	0.015	0.000	0.001	0.025	0.025	0.017																										
13	LC068715.1_1994	0.023	0.017	0.025	0.015	0.015	0.000	0.001	0.025	0.025	0.017	0.000																									
14	JX173078.1_2000	0.002	0.021	0.031	0.019	0.019	0.023	0.022	0.031	0.031	0.020	0.023	0.023																								
15	JX173080.1_2001	0.006	0.021	0.032	0.019	0.019	0.024	0.023	0.032	0.032	0.020	0.024	0.024	0.007																							
16	JX173081.1_2001	0.018	0.014	0.028	0.017	0.017	0.024	0.023	0.028	0.028	0.014	0.024	0.024	0.019	0.018																						
17	HQ003817.1_2001	0.027	0.024	0.028	0.022	0.022	0.010	0.010	0.028	0.028	0.024	0.010	0.010	0.027	0.028	0.028																					
18	JX173079.1_2002	0.015	0.013	0.027	0.016	0.016	0.019	0.018	0.027	0.027	0.013	0.019	0.019	0.016	0.017	0.015	0.024																				
19	KX384959.1_2002	0.015	0.013	0.027	0.016	0.016	0.019	0.018	0.027	0.027	0.013	0.019	0.019	0.016	0.016	0.015	0.024	0.001																			
20	JX173085.1_2003	0.004	0.019	0.030	0.018	0.018	0.023	0.022	0.030	0.030	0.019	0.023	0.023	0.005	0.005	0.018	0.027	0.016	0.016																		
21	JX173083.1_2003	0.003	0.019	0.030	0.017	0.017	0.022	0.022	0.030	0.030	0.019	0.022	0.022	0.004	0.005	0.017	0.027	0.015	0.015	0.000																	
22	JX173082.1_2003	0.001	0.020	0.030	0.018	0.018	0.022	0.022	0.030	0.030	0.019	0.022	0.022	0.002	0.006	0.018	0.027	0.015	0.015	0.004	0.003																
23	JX173084.1_2003	0.020	0.001	0.024	0.004	0.004	0.017	0.016	0.024	0.024	0.001	0.017	0.017	0.021	0.021	0.014	0.024	0.013	0.013	0.019	0.019	0.020															
24	LC068716.1_2003	0.023	0.017	0.025	0.015	0.015	0.000	0.001	0.025	0.025	0.018	0.000	0.000	0.023	0.024	0.024	0.010	0.019	0.019	0.023	0.023	0.023	0.017														
25	JX173086.1_2004	0.003	0.019	0.030	0.017	0.017	0.022	0.022	0.030	0.030	0.019	0.022	0.022	0.004	0.005	0.017	0.027	0.015	0.015	0.000	0.000	0.003	0.019	0.023													
26	KF268130.1_2004	0.020	0.001	0.024	0.005	0.005	0.017	0.016	0.024	0.024	0.001	0.017	0.017	0.021	0.021	0.014	0.024	0.013	0.013	0.020	0.019	0.020	0.001	0.017	0.019												
27	LC068718.1_2004	0.023	0.017	0.025	0.015	0.015	0.000	0.001	0.025	0.025	0.018	0.000	0.000	0.023	0.024	0.024	0.010	0.019	0.019	0.023	0.023	0.023	0.017	0.000	0.023	0.017											
28	LC068717.1_2004	0.022	0.016	0.025	0.014	0.014	0.001	0.000	0.025	0.025	0.017	0.001	0.001	0.022	0.024	0.023	0.010	0.018	0.018	0.022	0.022	0.022	0.017	0.001	0.022	0.016	0.001										
30	JX173077.1_2005	0.019	0.000	0.023	0.004	0.004	0.017	0.016	0.023	0.023	0.001	0.017	0.017	0.020	0.020	0.013	0.024	0.013	0.013	0.019	0.018	0.019	0.000	0.017	0.018	0.001	0.017	0.016									
31	KF268129.1_2005	0.030	0.028	0.024	0.028	0.028	0.025	0.025	0.024	0.024	0.029	0.025	0.025	0.031	0.032	0.033	0.027	0.031	0.031	0.031	0.030	0.030	0.028	0.026	0.030	0.029	0.026	0.025	0.028								
32	LC068720.1_2005	0.022	0.016	0.025	0.014	0.014	0.001	0.000	0.025	0.025	0.017	0.001	0.001	0.022	0.024	0.023	0.010	0.018	0.018	0.022	0.022	0.022	0.017	0.001	0.022	0.016	0.001	0.000	0.016	0.025							
33	LC068719.1_2005	0.022	0.016	0.025	0.014	0.014	0.001	0.000	0.025	0.025	0.017	0.001	0.001	0.022	0.024	0.023	0.010	0.018	0.018	0.022	0.022	0.022	0.017	0.001	0.022	0.016	0.001	0.000	0.016	0.025	0.000						
34	JX423389.1_2007	0.023	0.017	0.025	0.015	0.015	0.001	0.001	0.025	0.025	0.017	0.001	0.001	0.023	0.024	0.024	0.011	0.019	0.019	0.023	0.022	0.022	0.017	0.001	0.022	0.017	0.001	0.001	0.017	0.025	0.001	0.001					
35	KF268199.1_2008	0.032	0.024	0.003	0.028	0.028	0.026	0.026	0.003	0.003	0.025	0.026	0.026	0.032	0.033	0.029	0.029	0.029	0.029	0.032	0.031	0.032	0.025	0.027	0.031	0.025	0.027	0.026	0.024	0.025	0.026	0.026	0.026				
36	KR699642_2009	0.013	0.016	0.029	0.016	0.016	0.023	0.023	0.029	0.029	0.016	0.023	0.023	0.014	0.014	0.021	0.027	0.017	0.017	0.012	0.012	0.013	0.016	0.024	0.012	0.016	0.024	0.023	0.015	0.032	0.023	0.023	0.023	0.031			
37	KF951595.1_2013	0.022	0.016	0.025	0.014	0.014	0.001	0.000	0.025	0.025	0.017	0.001	0.001	0.022	0.023	0.023	0.010	0.018	0.018	0.022	0.022	0.022	0.016	0.001	0.022	0.016	0.001	0.000	0.016	0.025	0.000	0.000	0.001	0.026	0.023		
45	BJ04-2012-2	0.020	**0.001**	0.024	0.005	0.005	0.018	0.017	0.024	0.024	0.002	0.018	0.018	0.021	0.021	0.014	0.024	0.014	0.014	0.020	0.019	0.020	**0.001**	0.018	0.019	0.002	0.018	0.017	**0.001**	0.029	0.017	0.017	0.018	0.025	0.016	0.017	
46	BJ09-2013-2	0.013	0.016	0.029	0.017	0.017	0.024	0.023	0.029	0.029	0.016	0.024	0.024	0.014	0.014	0.021	0.028	0.018	0.018	0.012	0.012	0.013	0.016	0.024	0.012	0.016	0.024	0.023	0.016	0.031	0.023	0.023	0.024	0.031	**0.002**	0.023	0.017

pLdistanceL21745-26000

	1	2	3	4	5	6	7	9	10	11	12	13	14	15	16	17	18	19	20	21	22	23	24	25	26	27	28	30	31	32	33	34	35	36	37	45
1 AF534906_1953																																				
2 NC_001405_1953	0.099																																			
3 M73260_1953	0.104	0.109																																		
4 FJ349096_1953	0.100	0.008	0.109																																	
5 HQ413315_1953	0.100	0.008	0.109	0.000																																
6 LC068712.1_1987	0.100	0.006	0.108	0.007	0.007																															
7 LC068713.1_1987	0.100	0.007	0.108	0.007	0.007	0.000																														
9 KF268127.1_1988	0.102	0.109	0.002	0.108	0.108	0.107	0.107																													
10 KF429754.1_1990	0.102	0.109	0.002	0.108	0.108	0.107	0.107	0.000																												
11 KF268310.1_1992	0.099	0.001	0.109	0.008	0.008	0.006	0.007	0.109	0.109																											
12 LC068714.1_1993	0.100	0.006	0.108	0.007	0.007	0.000	0.000	0.107	0.107	0.006																										
13 LC068715.1_1994	0.101	0.007	0.109	0.008	0.008	0.001	0.001	0.108	0.108	0.007	0.001																									
14 JX173078.1_2000	0.000	0.100	0.104	0.100	0.100	0.100	0.100	0.102	0.102	0.099	0.100	0.101																								
15 JX173080.1_2001	0.095	0.018	0.110	0.018	0.018	0.018	0.018	0.108	0.108	0.017	0.018	0.019	0.095																							
16 JX173081.1_2001	0.096	0.012	0.112	0.017	0.017	0.014	0.015	0.110	0.110	0.012	0.014	0.015	0.097	0.014																						
17 HQ003817.1_2001	0.102	0.020	0.112	0.013	0.013	0.017	0.017	0.111	0.111	0.020	0.017	0.017	0.102	0.019	0.023																					
18 JX173079.1_2002	0.099	0.004	0.109	0.008	0.008	0.006	0.007	0.108	0.108	0.004	0.006	0.007	0.099	0.017	0.013	0.019																				
19 KX384959.1_2002	0.100	0.004	0.110	0.009	0.009	0.007	0.007	0.109	0.109	0.004	0.007	0.008	0.100	0.017	0.013	0.019	0.001																			
20 JX173085.1_2003	0.003	0.100	0.105	0.101	0.101	0.101	0.101	0.103	0.103	0.100	0.101	0.101	0.003	0.096	0.097	0.103	0.100	0.100																		
21 JX173083.1_2003	0.003	0.100	0.105	0.101	0.101	0.100	0.100	0.103	0.103	0.100	0.100	0.101	0.003	0.095	0.097	0.103	0.100	0.100	0.000																	
22 JX173082.1_2003	0.000	0.100	0.104	0.100	0.100	0.100	0.100	0.102	0.102	0.099	0.100	0.101	0.001	0.095	0.097	0.102	0.099	0.100	0.003	0.003																
23 JX173084.1_2003	0.100	0.001	0.110	0.009	0.009	0.006	0.007	0.109	0.109	0.001	0.006	0.007	0.100	0.018	0.013	0.020	0.004	0.004	0.100	0.100	0.100															
24 LC068716.1_2003	0.100	0.007	0.108	0.007	0.007	0.000	0.001	0.108	0.108	0.007	0.000	0.001	0.100	0.018	0.014	0.017	0.007	0.007	0.101	0.101	0.100	0.007														
25 JX173086.1_2004	0.003	0.100	0.105	0.101	0.101	0.100	0.100	0.103	0.103	0.100	0.100	0.101	0.003	0.095	0.097	0.103	0.100	0.100	0.000	0.000	0.003	0.100	0.101													
26 KF268130.1_2004	0.099	0.001	0.109	0.009	0.009	0.006	0.007	0.109	0.109	0.001	0.006	0.007	0.100	0.018	0.013	0.020	0.004	0.004	0.100	0.100	0.100	0.002	0.007	0.100												
27 LC068718.1_2004	0.100	0.006	0.108	0.007	0.007	0.000	0.001	0.108	0.108	0.006	0.000	0.001	0.100	0.018	0.014	0.017	0.006	0.007	0.101	0.101	0.100	0.007	0.001	0.101	0.007											
28 LC068717.1_2004	0.100	0.006	0.108	0.007	0.007	0.001	0.001	0.107	0.107	0.006	0.001	0.001	0.100	0.018	0.014	0.017	0.006	0.007	0.101	0.101	0.100	0.007	0.001	0.101	0.007	0.001										
30 JX173077.1_2005	0.099	0.001	0.109	0.008	0.008	0.006	0.006	0.109	0.109	0.000	0.006	0.007	0.099	0.017	0.012	0.019	0.003	0.004	0.100	0.100	0.099	0.001	0.006	0.100	0.001	0.006	0.006									
31 KF268129.1_2005	0.101	0.082	0.115	0.082	0.082	0.083	0.083	0.113	0.113	0.082	0.083	0.083	0.101	0.084	0.086	0.087	0.081	0.082	0.102	0.102	0.101	0.083	0.083	0.102	0.082	0.083	0.082	0.082								
32 LC068720.1_2005	0.100	0.006	0.108	0.007	0.007	0.001	0.001	0.107	0.107	0.006	0.001	0.001	0.100	0.018	0.014	0.017	0.006	0.007	0.100	0.100	0.100	0.007	0.001	0.100	0.007	0.001	0.000	0.006	0.082							
33 LC068719.1_2005	0.100	0.006	0.108	0.007	0.007	0.001	0.001	0.107	0.107	0.006	0.001	0.001	0.100	0.018	0.014	0.017	0.006	0.007	0.100	0.100	0.100	0.007	0.001	0.100	0.007	0.001	0.000	0.006	0.082	0.000						
34 JX423389.1_2007	0.100	0.006	0.108	0.007	0.007	0.000	0.000	0.107	0.107	0.006	0.000	0.001	0.100	0.018	0.014	0.017	0.006	0.007	0.101	0.100	0.100	0.006	0.000	0.100	0.006	0.000	0.001	0.006	0.083	0.001	0.001					
35 KF268199.1_2008	0.103	0.111	0.006	0.110	0.110	0.109	0.109	0.003	0.003	0.110	0.109	0.110	0.103	0.110	0.112	0.112	0.110	0.110	0.104	0.104	0.103	0.111	0.109	0.104	0.111	0.109	0.109	0.110	0.114	0.109	0.109	0.109				
36 KR699642_2009	0.099	0.004	0.109	0.010	0.010	0.006	0.006	0.109	0.109	0.004	0.006	0.007	0.099	0.018	0.013	0.021	0.005	0.005	0.100	0.100	0.099	0.005	0.006	0.100	0.005	0.006	0.006	0.004	0.083	0.006	0.006	0.006	0.110			
37 KF951595.1_2013	0.100	0.007	0.109	0.008	0.008	0.001	0.001	0.108	0.108	0.007	0.001	0.001	0.101	0.019	0.015	0.017	0.007	0.007	0.101	0.101	0.101	0.007	0.001	0.101	0.007	0.001	0.001	0.007	0.083	0.001	0.001	0.001	0.109	0.006		
45 BJ04-2012-2	0.100	0.002	0.110	0.009	0.009	0.007	0.007	0.110	0.110	0.002	0.007	0.008	0.100	0.018	0.013	0.021	0.004	0.005	0.101	0.101	0.100	0.003	0.007	0.101	0.003	0.007	0.007	0.002	0.083	0.007	0.007	0.007	0.111	0.005	0.008	
46 BJ09-2013-2	0.100	0.004	0.109	0.010	0.010	0.006	0.006	0.109	0.109	0.004	0.006	0.006	0.100	0.018	0.013	0.021	0.005	0.005	0.100	0.100	0.100	0.004	0.006	0.100	0.004	0.006	0.006	0.004	0.083	0.006	0.006	0.006	0.110	0.001	0.006	0.005

pLdistanceL26001-31029

	1	2	3	4	5	6	7	9	10	11	12	13	14	15	16	17	18	19	20	21	22	23	24	25	26	27	28	30	31	32	33	34	35	36	37	45
1 AF534906_1953																																				
2 NC_001405_1953	0.268																																			
3 M73260_1953	0.249	0.269																																		
4 FJ349096_1953	0.225	0.246	0.282																																	
5 HQ413315_1953	0.225	0.246	0.282	0.000																																
6 LC068712.1_1987	0.225	0.248	0.284	0.004	0.004																															
7 LC068713.1_1987	0.225	0.248	0.284	0.004	0.004	0.000																														
9 KF268127.1_1988	0.249	0.269	0.000	0.282	0.282	0.284	0.284																													
10 KF429754.1_1990	0.249	0.269	0.000	0.282	0.282	0.284	0.284	0.000																												
11 KF268310.1_1992	0.267	0.001	0.269	0.246	0.246	0.249	0.249	0.269	0.269																											
12 LC068714.1_1993	0.225	0.248	0.284	0.004	0.004	0.000	0.000	0.284	0.284	0.249																										
13 LC068715.1_1994	0.225	0.248	0.284	0.004	0.004	0.000	0.000	0.284	0.284	0.249	0.000																									
14 JX173078.1_2000	0.001	0.269	0.249	0.226	0.226	0.226	0.226	0.249	0.249	0.268	0.226	0.226																								
15 JX173080.1_2001	0.010	0.266	0.245	0.221	0.221	0.221	0.221	0.245	0.245	0.265	0.221	0.221	0.009																							
16 JX173081.1_2001	0.268	0.004	0.268	0.247	0.247	0.250	0.250	0.268	0.268	0.005	0.250	0.250	0.269	0.266																						
17 HQ003817.1_2001	0.226	0.250	0.285	0.004	0.004	0.001	0.001	0.285	0.285	0.250	0.001	0.001	0.227	0.222	0.251																					
18 JX173079.1_2002	0.268	0.003	0.267	0.246	0.246	0.248	0.248	0.267	0.267	0.005	0.248	0.248	0.269	0.266	0.005	0.250																				
19 KX384959.1_2002	0.267	0.003	0.267	0.247	0.247	0.250	0.250	0.267	0.267	0.005	0.250	0.250	0.268	0.265	0.005	0.251	0.001																			
20 JX173085.1_2003	0.007	0.266	0.248	0.222	0.222	0.222	0.222	0.248	0.248	0.265	0.222	0.222	0.006	0.006	0.266	0.224	0.266	0.265																		
21 JX173083.1_2003	0.008	0.266	0.248	0.223	0.223	0.223	0.223	0.248	0.248	0.266	0.223	0.223	0.007	0.007	0.267	0.224	0.267	0.266	0.001																	
22 JX173082.1_2003	0.001	0.268	0.248	0.225	0.225	0.225	0.225	0.248	0.248	0.268	0.225	0.225	0.001	0.010	0.269	0.226	0.269	0.268	0.007	0.007																
23 JX173084.1_2003	0.267	0.001	0.269	0.246	0.246	0.249	0.249	0.269	0.269	0.000	0.249	0.249	0.268	0.265	0.005	0.250	0.005	0.005	0.265	0.266	0.268															
24 LC068716.1_2003	0.225	0.248	0.284	0.004	0.004	0.000	0.000	0.284	0.284	0.249	0.000	0.000	0.226	0.221	0.250	0.001	0.248	0.250	0.222	0.223	0.225	0.249														
25 JX173086.1_2004	0.008	0.266	0.248	0.223	0.223	0.223	0.223	0.248	0.248	0.266	0.223	0.223	0.007	0.007	0.267	0.224	0.267	0.266	0.001	0.001	0.007	0.266	0.223													
26 KF268130.1_2004	0.268	0.001	0.269	0.246	0.246	0.248	0.248	0.269	0.269	0.002	0.248	0.248	0.269	0.266	0.005	0.250	0.004	0.004	0.266	0.267	0.269	0.002	0.248	0.267												
27 LC068718.1_2004	0.225	0.248	0.284	0.004	0.004	0.000	0.000	0.284	0.284	0.249	0.000	0.000	0.226	0.221	0.250	0.001	0.248	0.250	0.222	0.223	0.225	0.249	0.000	0.223	0.248											
28 LC068717.1_2004	0.224	0.248	0.282	0.006	0.006	0.002	0.002	0.282	0.282	0.248	0.002	0.002	0.225	0.221	0.249	0.003	0.248	0.249	0.222	0.222	0.224	0.248	0.002	0.222	0.248	0.002										
30 JX173077.1_2005	0.267	0.001	0.269	0.246	0.246	0.249	0.249	0.269	0.269	0.000	0.249	0.249	0.268	0.265	0.005	0.250	0.005	0.005	0.265	0.266	0.268	0.000	0.249	0.266	0.002	0.249	0.248									
31 KF268129.1_2005	0.223	0.245	0.281	0.018	0.018	0.020	0.020	0.281	0.281	0.246	0.020	0.020	0.224	0.219	0.246	0.020	0.245	0.246	0.221	0.221	0.223	0.246	0.020	0.221	0.245	0.020	0.021	0.246								
32 LC068720.1_2005	0.224	0.248	0.283	0.004	0.004	0.001	0.001	0.283	0.283	0.248	0.001	0.001	0.225	0.221	0.249	0.002	0.248	0.249	0.222	0.222	0.224	0.248	0.001	0.222	0.248	0.001	0.001	0.248	0.020							
33 LC068719.1_2005	0.224	0.248	0.283	0.004	0.004	0.001	0.001	0.283	0.283	0.248	0.001	0.001	0.225	0.221	0.249	0.002	0.248	0.249	0.222	0.222	0.224	0.248	0.001	0.222	0.248	0.001	0.001	0.248	0.020	0.000						
34 JX423389.1_2007	0.224	0.249	0.283	0.004	0.004	0.001	0.001	0.283	0.283	0.250	0.001	0.001	0.225	0.222	0.250	0.002	0.249	0.250	0.223	0.224	0.224	0.250	0.001	0.224	0.249	0.001	0.003	0.250	0.020	0.001	0.001					
35 KF268199.1_2008	0.250	0.270	0.002	0.284	0.284	0.285	0.285	0.002	0.002	0.270	0.285	0.285	0.250	0.246	0.269	0.286	0.268	0.268	0.249	0.250	0.250	0.270	0.285	0.250	0.271	0.285	0.283	0.270	0.282	0.284	0.284	0.284				
36 KR699642_2009	0.268	0.003	0.269	0.246	0.246	0.248	0.248	0.269	0.269	0.004	0.248	0.248	0.269	0.266	0.005	0.250	0.004	0.004	0.266	0.266	0.268	0.004	0.248	0.266	0.003	0.248	0.248	0.004	0.245	0.248	0.248	0.249	0.270			
37 KF951595.1_2013	0.225	0.248	0.284	0.004	0.004	0.000	0.000	0.284	0.284	0.249	0.000	0.000	0.226	0.221	0.250	0.001	0.248	0.250	0.222	0.223	0.225	0.249	0.000	0.223	0.248	0.000	0.002	0.249	0.020	0.001	0.001	0.001	0.285	0.248		
45 BJ04-2012-2	0.268	0.001	0.269	0.246	0.246	0.249	0.249	0.269	0.269	0.002	0.249	0.249	0.269	0.266	0.003	0.250	0.003	0.003	0.266	0.267	0.269	0.002	0.249	0.267	0.001	0.249	0.248	0.002	0.246	0.248	0.248	0.250	0.271	0.002	0.249	
46 BJ09-2013-2	0.268	0.002	0.269	0.246	0.246	0.249	0.249	0.269	0.269	0.003	0.249	0.249	0.269	0.266	0.003	0.250	0.003	0.003	0.266	0.267	0.269	0.003	0.249	0.267	0.002	0.249	0.248	0.003	0.246	0.248	0.248	0.250	0.271	0.001	0.249	0.001

pLdistanceL31030-32778

	1	2	3	4	5	6	7	9	10	11	12	13	14	15	16	17	18	19	20	21	22	23	24	25	26	27	28	30	31	32	33	34	35	36	37	45
1 AF534906_1953																																				
2 NC_001405_1953	0.003																																			
3 M73260_1953	0.011	0.011																																		
4 FJ349096_1953	0.007	0.005	0.013																																	
5 HQ413315_1953	0.007	0.005	0.013	0.000																																
6 LC068712.1_1987	0.018	0.016	0.017	0.016	0.016																															
7 LC068713.1_1987	0.017	0.015	0.018	0.016	0.016	0.005																														
9 KF268127.1_1988	0.011	0.011	0.001	0.012	0.013	0.017	0.017																													
10 KF429754.1_1990	0.011	0.011	0.001	0.012	0.013	0.017	0.018	0.000																												
11 KF268310.1_1992	0.004	0.001	0.011	0.005	0.005	0.016	0.015	0.011	0.011																											
12 LC068714.1_1993	0.017	0.015	0.017	0.014	0.015	0.011	0.011	0.017	0.017	0.016																										
13 LC068715.1_1994	0.017	0.015	0.017	0.014	0.014	0.011	0.010	0.017	0.017	0.015	0.001																									
14 JX173078.1_2000	0.002	0.002	0.010	0.005	0.006	0.017	0.016	0.010	0.010	0.003	0.016	0.015																								
15 JX173080.1_2001	0.016	0.016	0.019	0.015	0.015	0.017	0.017	0.019	0.019	0.016	0.012	0.012	0.015																							
16 JX173081.1_2001	0.014	0.013	0.011	0.015	0.015	0.016	0.017	0.010	0.011	0.013	0.018	0.018	0.014	0.022																						
17 HQ003817.1_2001	0.010	0.009	0.016	0.011	0.011	0.016	0.015	0.016	0.016	0.009	0.010	0.010	0.009	0.011	0.017																					
18 JX173079.1_2002	0.013	0.011	0.013	0.012	0.012	0.017	0.018	0.012	0.012	0.011	0.018	0.018	0.012	0.020	0.015	0.017																				
19 KX384959.1_2002	0.014	0.013	0.013	0.013	0.013	0.019	0.020	0.013	0.013	0.013	0.020	0.019	0.014	0.022	0.017	0.018	0.003																			
20 JX173085.1_2003	0.008	0.006	0.007	0.008	0.008	0.015	0.016	0.006	0.006	0.006	0.014	0.014	0.007	0.014	0.009	0.010	0.011	0.012																		
21 JX173083.1_2003	0.008	0.006	0.007	0.008	0.008	0.015	0.016	0.006	0.006	0.006	0.014	0.014	0.007	0.014	0.009	0.010	0.011	0.012	0.000																	
22 JX173082.1_2003	0.001	0.003	0.011	0.006	0.006	0.017	0.017	0.010	0.010	0.003	0.017	0.016	0.001	0.016	0.014	0.010	0.013	0.014	0.007	0.007																
23 JX173084.1_2003	0.003	0.001	0.011	0.005	0.005	0.016	0.015	0.011	0.011	0.001	0.015	0.015	0.002	0.016	0.013	0.009	0.011	0.013	0.006	0.006	0.003															
24 LC068716.1_2003	0.017	0.015	0.015	0.014	0.014	0.009	0.010	0.015	0.015	0.014	0.011	0.010	0.015	0.016	0.016	0.014	0.017	0.018	0.014	0.014	0.016	0.015														
25 JX173086.1_2004	0.007	0.007	0.006	0.009	0.009	0.016	0.017	0.005	0.005	0.007	0.015	0.015	0.006	0.013	0.010	0.011	0.012	0.013	0.001	0.001	0.006	0.007	0.015													
26 KF268130.1_2004	0.003	0.001	0.011	0.005	0.005	0.016	0.015	0.011	0.011	0.002	0.016	0.015	0.002	0.016	0.013	0.009	0.012	0.013	0.006	0.006	0.003	0.000	0.015	0.007												
27 LC068718.1_2004	0.017	0.015	0.016	0.014	0.014	0.001	0.004	0.015	0.016	0.015	0.010	0.010	0.015	0.016	0.015	0.014	0.016	0.018	0.014	0.014	0.016	0.015	0.008	0.015	0.015											
28 LC068717.1_2004	0.014	0.012	0.018	0.011	0.011	0.013	0.012	0.018	0.018	0.012	0.007	0.006	0.013	0.010	0.019	0.006	0.019	0.020	0.013	0.013	0.013	0.012	0.012	0.014	0.012	0.011										
30 JX173077.1_2005	0.004	0.002	0.012	0.006	0.006	0.017	0.016	0.012	0.012	0.001	0.016	0.016	0.003	0.016	0.014	0.010	0.012	0.013	0.007	0.007	0.003	0.002	0.016	0.008	0.002	0.016	0.013									
31 KF268129.1_2005	0.018	0.017	0.017	0.016	0.016	0.012	0.014	0.016	0.016	0.017	0.013	0.013	0.018	0.016	0.019	0.016	0.017	0.019	0.016	0.016	0.018	0.017	0.008	0.017	0.017	0.011	0.015	0.018								
32 LC068720.1_2005	0.014	0.012	0.018	0.011	0.011	0.013	0.012	0.018	0.018	0.012	0.007	0.006	0.013	0.010	0.019	0.006	0.019	0.020	0.013	0.013	0.013	0.012	0.012	0.014	0.012	0.011	0.000	0.013	0.015							
33 LC068719.1_2005	0.014	0.012	0.018	0.011	0.011	0.012	0.012	0.018	0.018	0.012	0.007	0.006	0.013	0.010	0.019	0.006	0.019	0.020	0.013	0.013	0.013	0.012	0.012	0.014	0.012	0.011	0.000	0.013	0.015	0.000						
34 JX423389.1_2007	0.015	0.013	0.020	0.012	0.012	0.014	0.013	0.019	0.020	0.013	0.009	0.008	0.014	0.011	0.020	0.008	0.020	0.021	0.014	0.014	0.014	0.013	0.013	0.015	0.013	0.012	0.001	0.014	0.017	0.001	0.001					
35 KF268199.1_2008	0.008	0.008	0.006	0.009	0.010	0.017	0.018	0.006	0.006	0.008	0.016	0.016	0.007	0.015	0.010	0.011	0.012	0.014	0.002	0.002	0.007	0.008	0.016	0.001	0.008	0.016	0.015	0.009	0.017	0.014	0.015	0.016				
36 KR699642_2009	0.010	0.009	0.008	0.010	0.011	0.014	0.015	0.008	0.008	0.009	0.016	0.016	0.010	0.017	0.007	0.013	0.012	0.013	0.004	0.004	0.010	0.009	0.014	0.005	0.009	0.013	0.015	0.010	0.016	0.015	0.015	0.016	0.006			
37 KF951595.1_2013	0.014	0.012	0.019	0.011	0.012	0.013	0.013	0.019	0.019	0.013	0.008	0.007	0.013	0.010	0.020	0.007	0.019	0.021	0.013	0.013	0.014	0.012	0.013	0.014	0.013	0.012	0.002	0.013	0.016	0.002	0.002	0.003	0.015	0.016		
45 BJ04-2012-2	0.019	0.017	0.018	0.016	0.017	0.011	0.012	0.017	0.017	0.018	0.013	0.013	0.018	0.017	0.019	0.016	0.018	0.020	0.016	0.016	0.018	0.017	0.011	0.017	0.017	0.011	0.014	0.018	0.013	0.014	0.014	0.015	0.018	0.016	0.014	
46 BJ09-2013-2	0.010	0.008	0.008	0.010	0.010	0.013	0.014	0.007	0.007	0.008	0.015	0.015	0.009	0.016	0.007	0.012	0.011	0.013	0.003	0.003	0.009	0.008	0.013	0.004	0.009	0.012	0.014	0.009	0.015	0.014	0.015	0.016	0.005	0.001	0.016	0.016

pLdistanceL32779-end

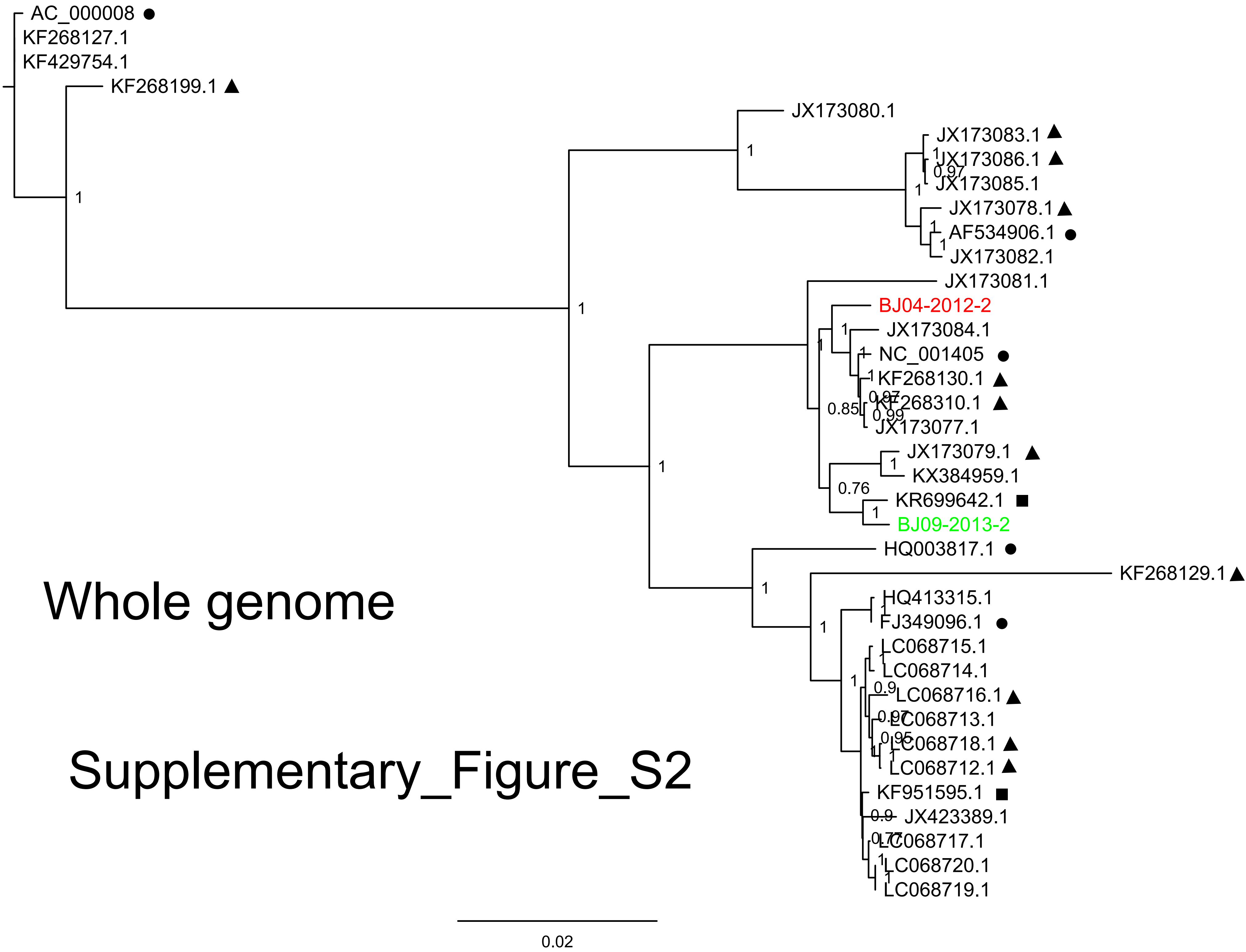

Supplementary_Figure_S2

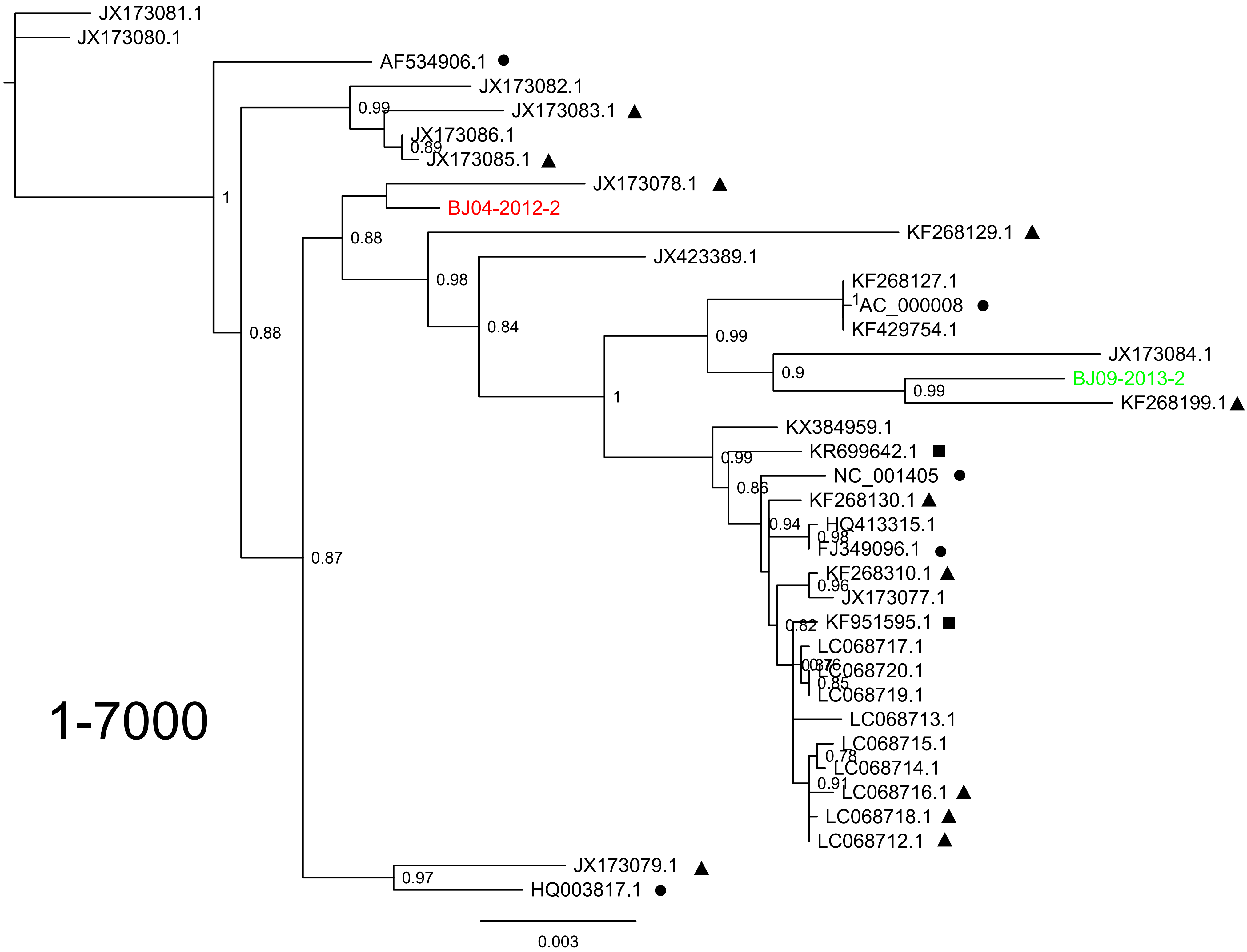
JX173081.1
JX173080.1
AF534906.1
JX173082.1
0.99
JX173083.1
JX173086.1
0.89
JX173085.1
JX173078.1
1
BJ04-2012-2
0.88
KF268129.1
JX423389.1
0.98
KF268127.1
1
AC_000008
KF429754.1
0.84
0.99
JX173084.1
0.9
BJ09-2013-2
0.99
KF268199.1
1
0.88
KX384959.1
KR699642.1
0.99
NC_001405
0.86
KF268130.1
0.94
HQ413315.1
0.98
FJ349096.1
KF268310.1
0.96
JX173077.1
0.87
KF951595.1
0.82
LC068717.1
LC068720.1
0.85
LC068719.1
LC068713.1
LC068715.1
0.78
LC068714.1
0.91
LC068716.1
LC068718.1
LC068712.1
JX173079.1
0.97
HQ003817.1
0.003
1-7000

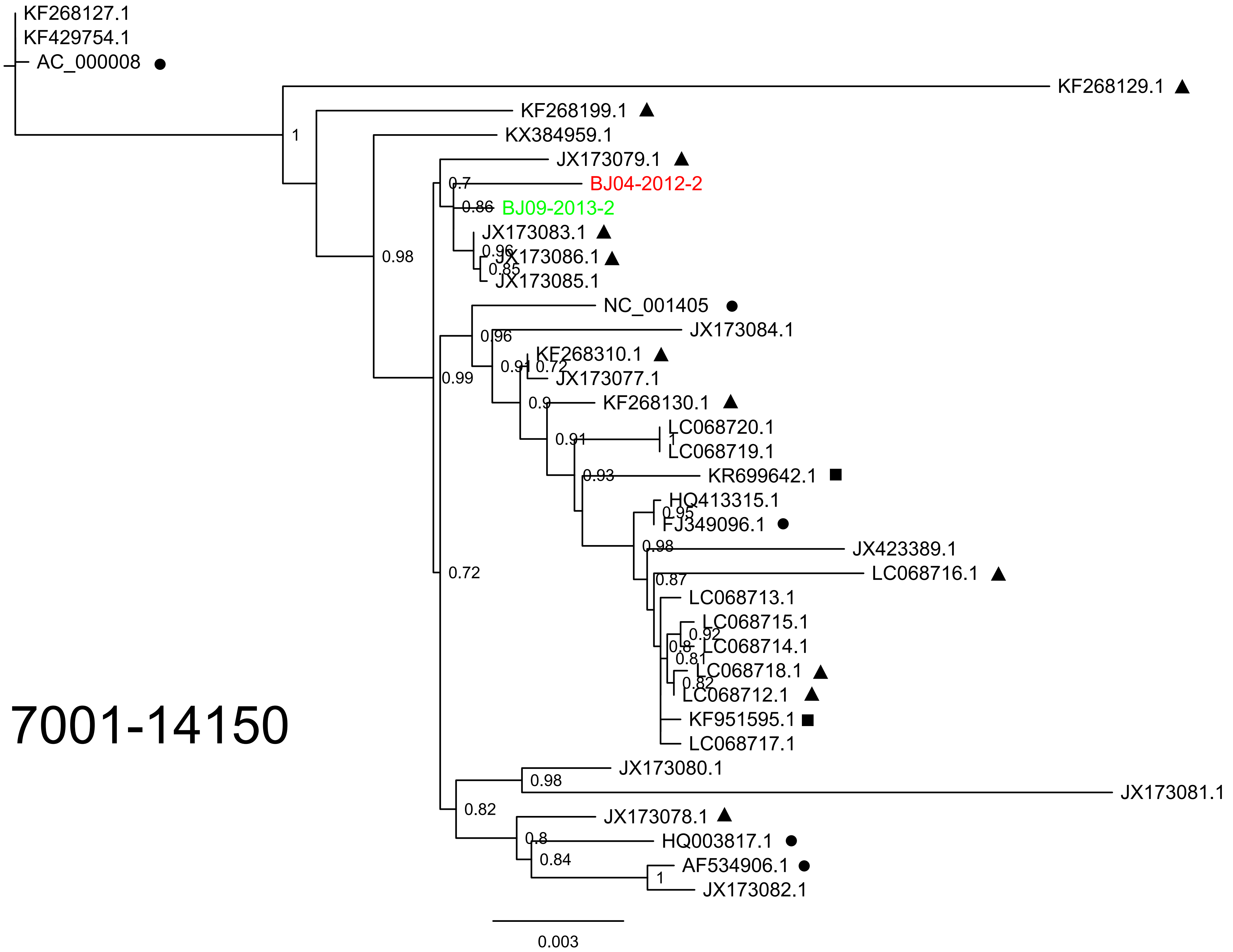

KF268127.1
KF429754.1
AC_000008
KF268129.1
KF268199.1
KX384959.1
JX173079.1
BJ04-2012-2
BJ09-2013-2
JX173083.1
JX173086.1
JX173085.1
NC_001405
JX173084.1
KF268310.1
JX173077.1
KF268130.1
LC068720.1
LC068719.1
KR699642.1
HQ413315.1
FJ349096.1
JX423389.1
LC068716.1
LC068713.1
LC068715.1
LC068714.1
LC068718.1
LC068712.1
KF951595.1
LC068717.1
JX173080.1
JX173081.1
JX173078.1
HQ003817.1
AF534906.1
JX173082.1
1
0.98
0.7
0.86
0.96
0.85
0.99
0.96
0.91
0.72
0.9
0.91
1
0.93
0.95
0.98
0.87
0.92
0.8
0.81
0.82
0.72
0.98
0.82
0.8
0.84
1
0.003
7001-14150

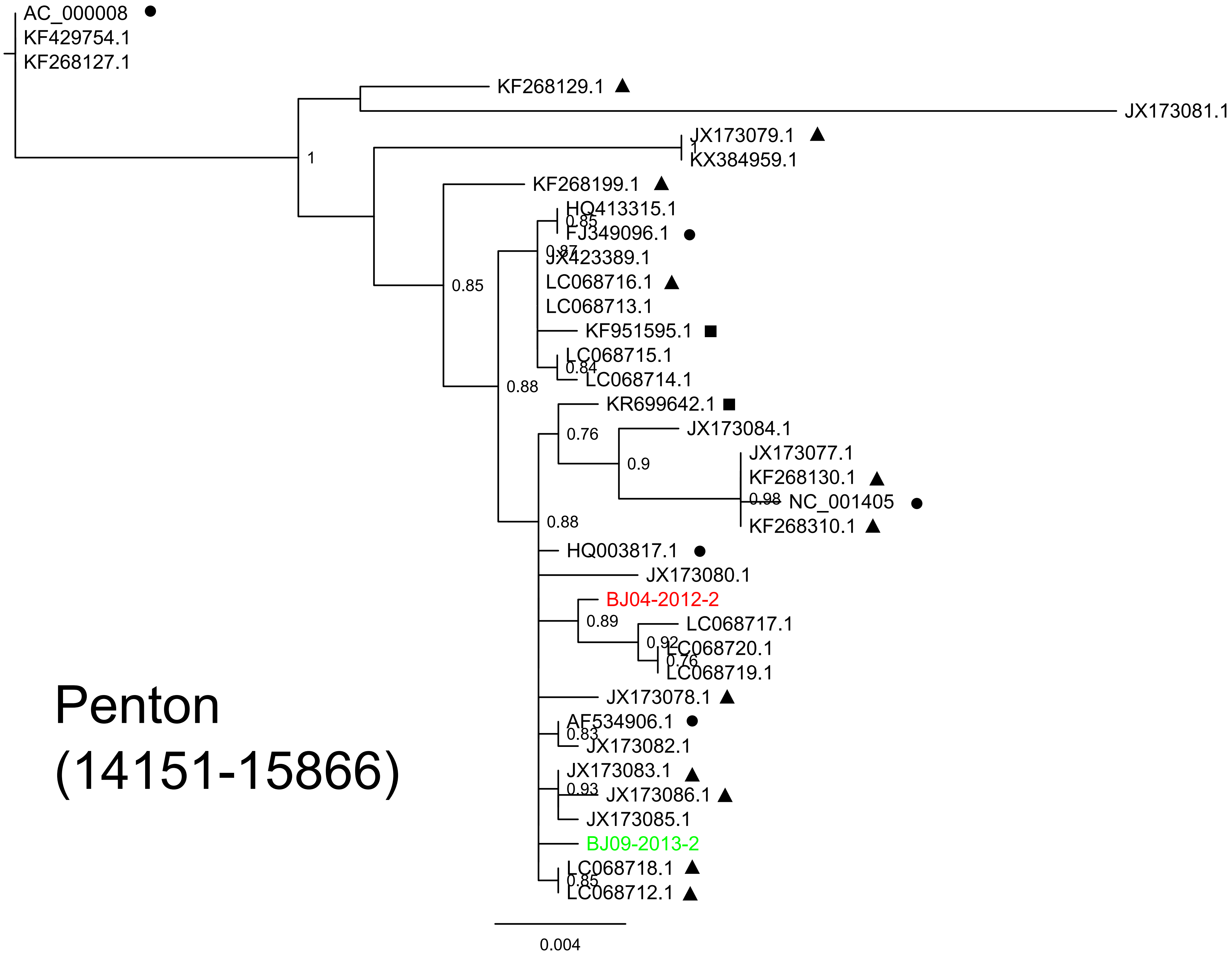
AC_000008
KF429754.1
KF268127.1
KF268129.1
JX173081.1
JX173079.1
KX384959.1
1
KF268199.1
HQ413315.1
0.85
FJ349096.1
0.87
JX423389.1
LC068716.1
LC068713.1
KF951595.1
LC068715.1
0.84
LC068714.1
0.85
0.88
KR699642.1
JX173084.1
0.76
JX173077.1
0.9
KF268130.1
0.98
NC_001405
KF268310.1
0.88
HQ003817.1
JX173080.1
BJ04-2012-2
0.89
LC068717.1
0.92
LC068720.1
0.76
LC068719.1
JX173078.1
AF534906.1
0.83
JX173082.1
JX173083.1
0.93
JX173086.1
JX173085.1
BJ09-2013-2
LC068718.1
0.85
LC068712.1
0.004
Penton
(14151-15866)

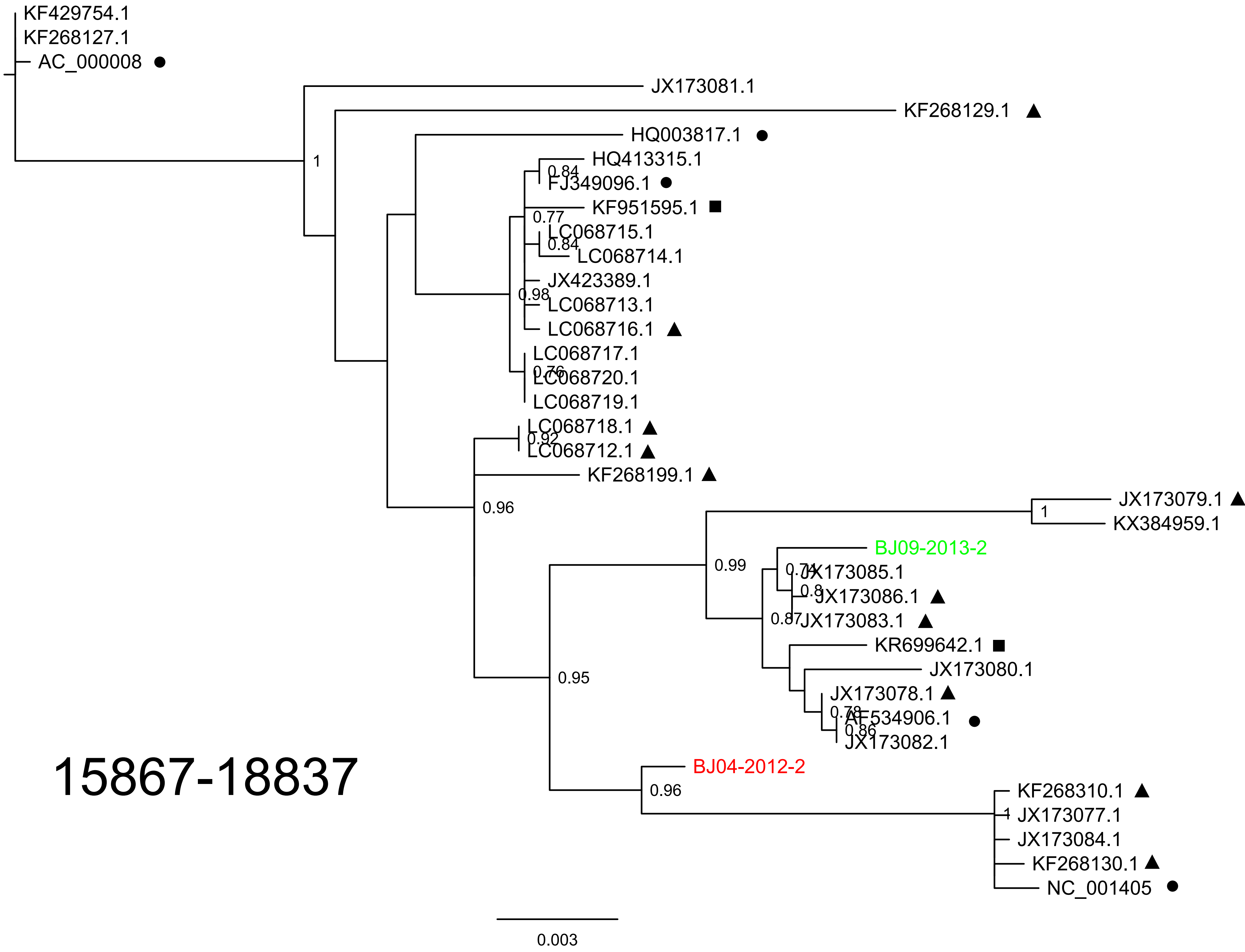
KF429754.1
KF268127.1
AC_000008
JX173081.1
KF268129.1
HQ003817.1
HQ413315.1
0.84
FJ349096.1
KF951595.1
0.77
LC068715.1
0.84
LC068714.1
JX423389.1
0.98
LC068713.1
LC068716.1
LC068717.1
0.76
LC068720.1
LC068719.1
LC068718.1
0.92
LC068712.1
KF268199.1
0.96
1
JX173079.1
KX384959.1
1
BJ09-2013-2
0.99
0.74
JX173085.1
0.8
JX173086.1
0.87
JX173083.1
KR699642.1
JX173080.1
0.95
JX173078.1
0.78
AF534906.1
0.86
JX173082.1
15867-18837
BJ04-2012-2
0.96
KF268310.1
1
JX173077.1
JX173084.1
KF268130.1
NC_001405
0.003

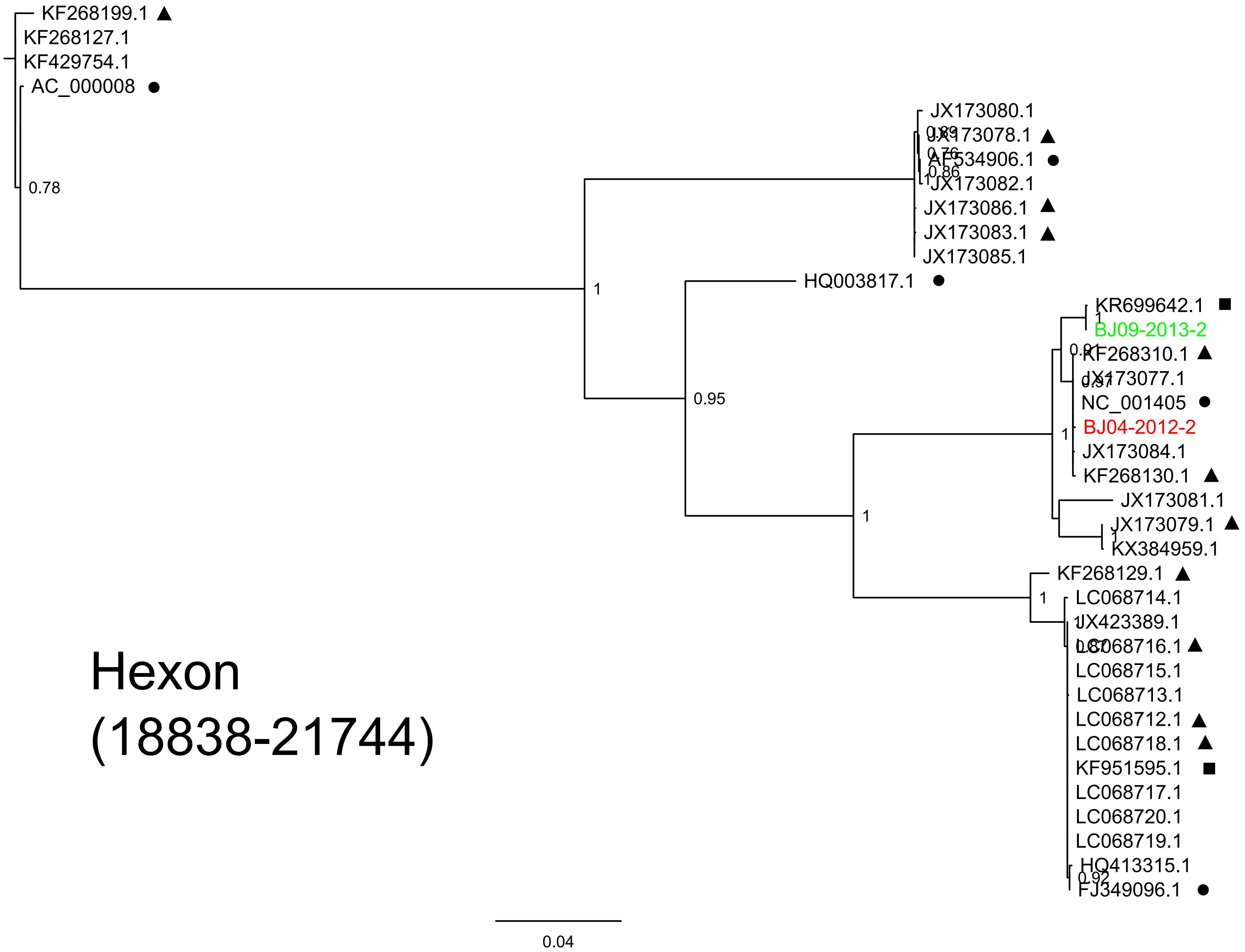

KF268199.1
KF268127.1
KF429754.1
AC_000008
0.78
JX173080.1
0.89
JX173078.1
0.76
AF534906.1
0.86
JX173082.1
JX173086.1
JX173083.1
JX173085.1
1
HQ003817.1
KR699642.1
1
BJ09-2013-2
0.91
KF268310.1
0.97
JX173077.1
NC_001405
BJ04-2012-2
1
JX173084.1
KF268130.1
0.95
JX173081.1
1
JX173079.1
1
KX384959.1
KF268129.1
1
LC068714.1
1
JX423389.1
0.87
LC068716.1
LC068715.1
LC068713.1
LC068712.1
LC068718.1
KF951595.1
LC068717.1
LC068720.1
LC068719.1
HQ413315.1
0.92
FJ349096.1
Hexon
(18838-21744)
0.04

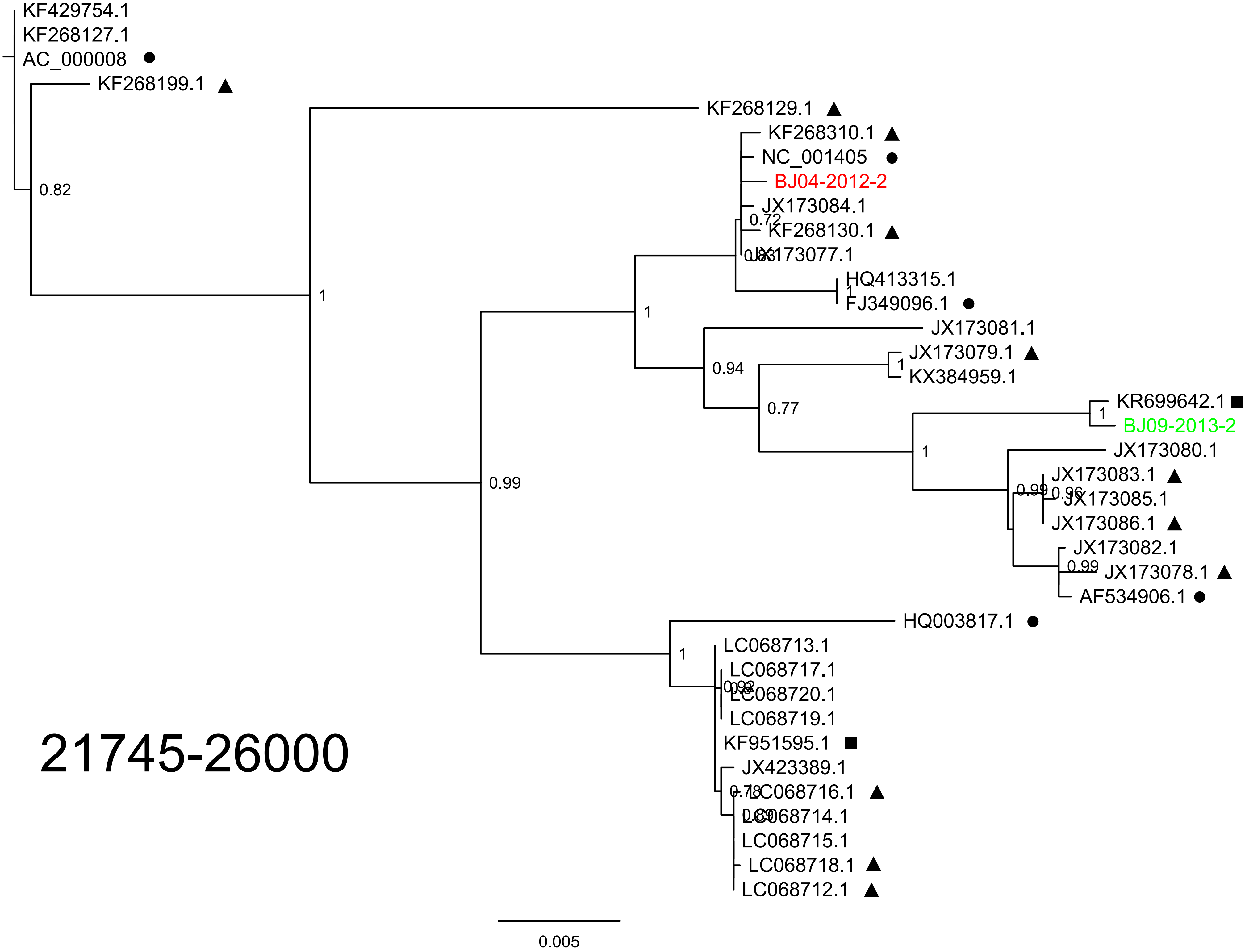

KF429754.1
KF268127.1
AC_000008
KF268199.1
0.82
1
KF268129.1
KF268310.1
NC_001405
BJ04-2012-2
JX173084.1
0.72
KF268130.1
HQ413315.1
FJ349096.1
1
JX173081.1
JX173079.1
KX384959.1
0.94
0.77
KR699642.1
BJ09-2013-2
JX173080.1
JX173083.1
JX173085.1
JX173086.1
JX173082.1
JX173078.1
AF534906.1
0.99
HQ003817.1
LC068713.1
LC068717.1
LC068720.1
LC068719.1
KF951595.1
JX423389.1
LC068716.1
LC068714.1
LC068715.1
LC068718.1
LC068712.1
21745-26000
0.005

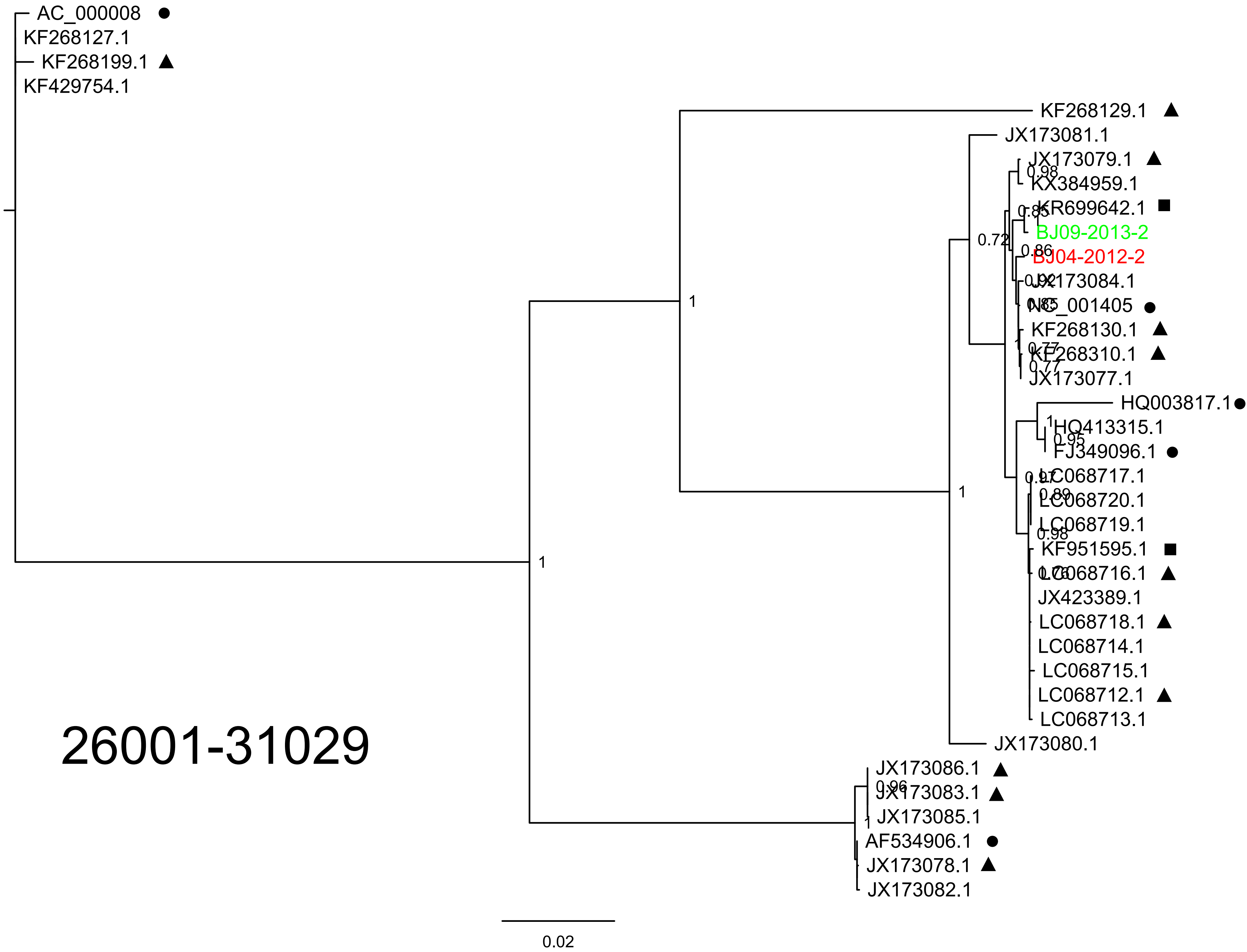
AC_000008
KF268127.1
KF268199.1
KF429754.1
KF268129.1
JX173081.1
JX173079.1
0.98
KX384959.1
0.85
KR699642.1
BJ09-2013-2
0.72
0.86
BJ04-2012-2
JX173084.1
NC_001405
KF268130.1
KF268310.1
0.77
JX173077.1
1
HQ003817.1
HQ413315.1
0.95
FJ349096.1
LC068717.1
0.89
LC068720.1
LC068719.1
0.98
KF951595.1
0.76
LC068716.1
JX423389.1
LC068718.1
LC068714.1
LC068715.1
LC068712.1
LC068713.1
JX173080.1
JX173086.1
0.96
JX173083.1
JX173085.1
AF534906.1
JX173078.1
JX173082.1
26001-31029
0.02

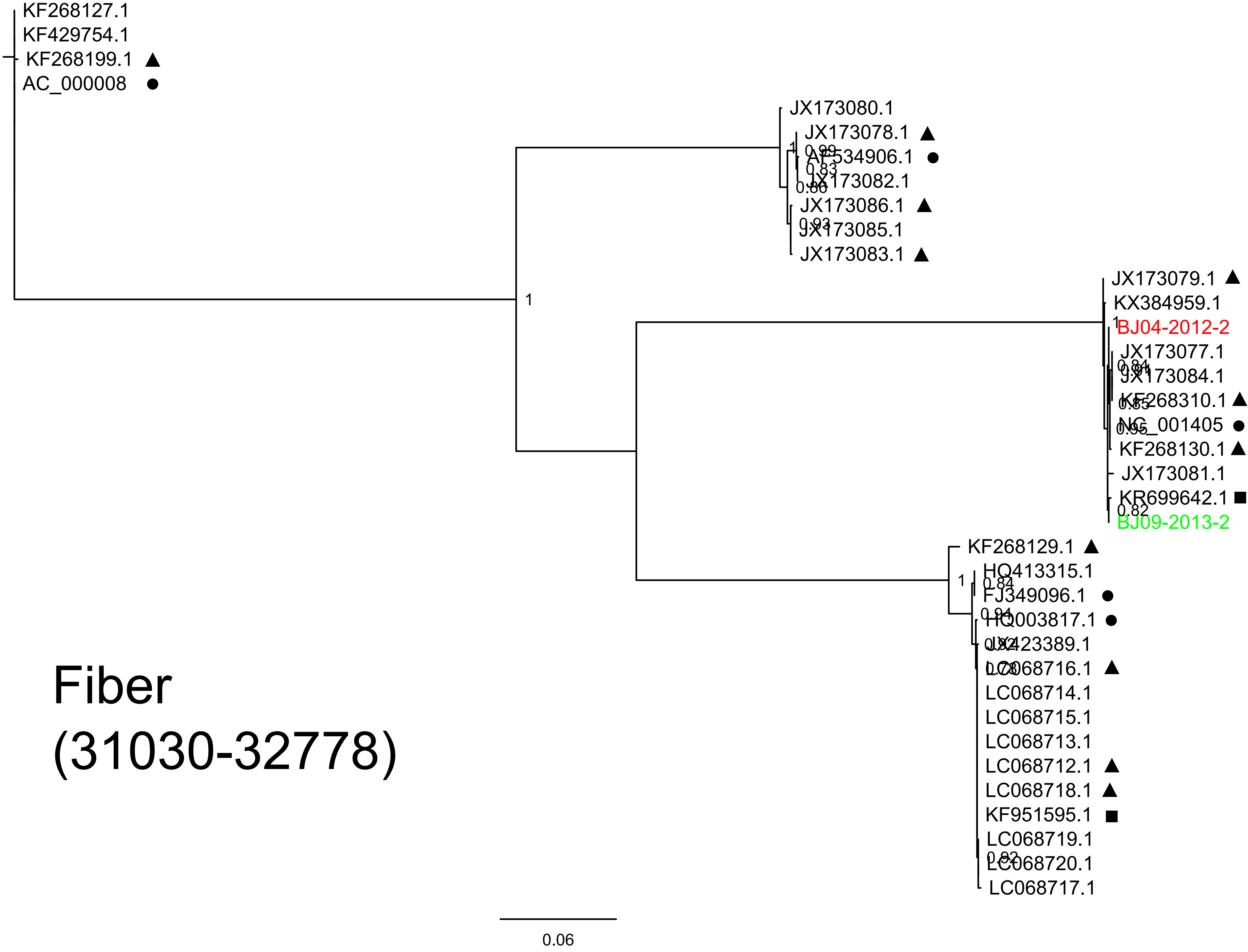

KF268127.1
KF429754.1
KF268199.1
AC_000008
JX173080.1
JX173078.1
AF534906.1
JX173082.1
JX173086.1
JX173085.1
JX173083.1
1
JX173079.1
KX384959.1
BJ04-2012-2
JX173077.1
JX173084.1
KF268310.1
NC_001405
KF268130.1
JX173081.1
KR699642.1
BJ09-2013-2
KF268129.1
HQ413315.1
FJ349096.1
HQ003817.1
JX423389.1
LC068716.1
LC068714.1
LC068715.1
LC068713.1
LC068712.1
LC068718.1
KF951595.1
LC068719.1
LC068720.1
LC068717.1
Fiber
(31030-32778)
0.06

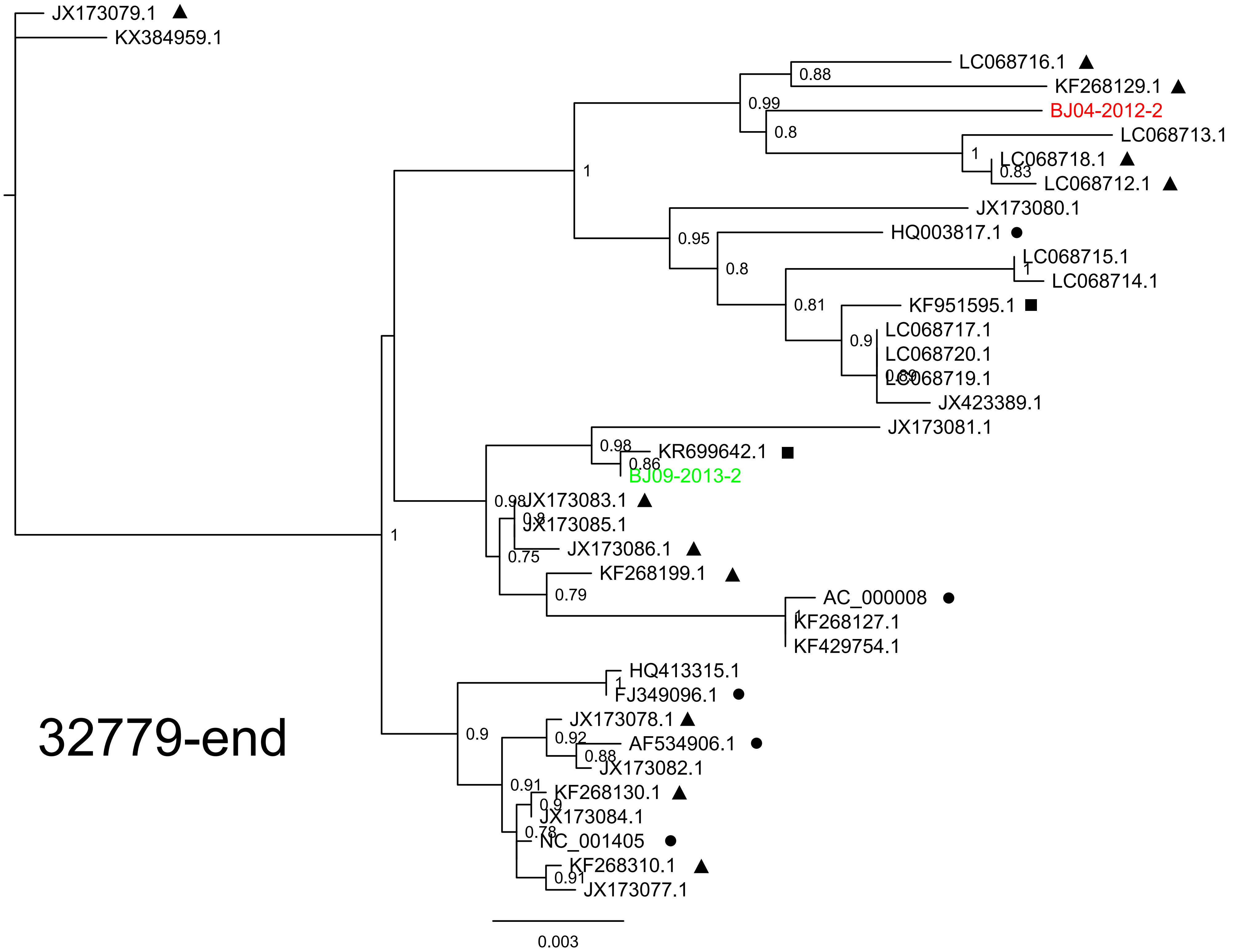
JX173079.1
KX384959.1
LC068716.1
KF268129.1
BJ04-2012-2
LC068713.1
LC068718.1
LC068712.1
JX173080.1
HQ003817.1
LC068715.1
LC068714.1
KF951595.1
LC068717.1
LC068720.1
LC068719.1
JX423389.1
JX173081.1
KR699642.1
BJ09-2013-2
JX173083.1
JX173085.1
JX173086.1
KF268199.1
AC_000008
KF268127.1
KF429754.1
HQ413315.1
FJ349096.1
JX173078.1
AF534906.1
JX173082.1
KF268130.1
JX173084.1
NC_001405
KF268310.1
JX173077.1
32779-end
0.003

BJ04

Bootscan

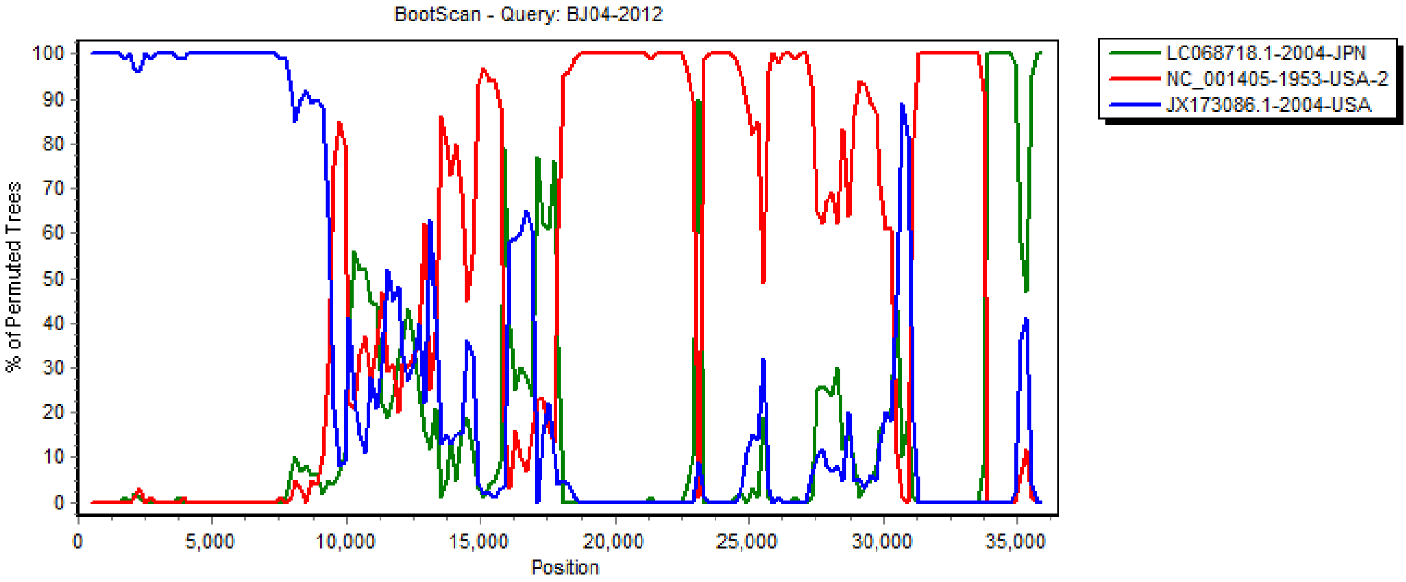

RDP4

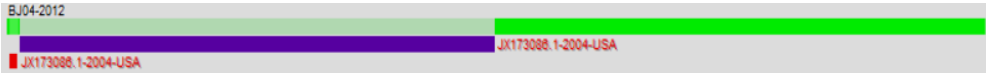

		Breakpoint analysis			
				p value on breakpoint	
**Methods**	**Average p-value on recombination event**	**Beginning breakpoint**	**Ending breakpoint**	**Beginning breakpoint**	**Ending breakpoint**
RDP	$7x10^{-82}$	undetermined	18078	N/A	
GENECONV	$8x10^{-20}$	4404	7949	N/A	
MaxChi	$3x10^{-22}$	undetermined	**18077**	N/A	$3x10^{-22}$
Chimaera	$1x10^{-7}$	undetermined	16852	N/A	$8x10^{-6}$
SiScan	$6x10^{-16}$	undetermined	15269	N/A	
3Seq	$1x10^{-12}$	undetermined	19255	N/A	

Breakpoint estimated at position 18077

Supplementary Figure S3

BJ09

Bootscan

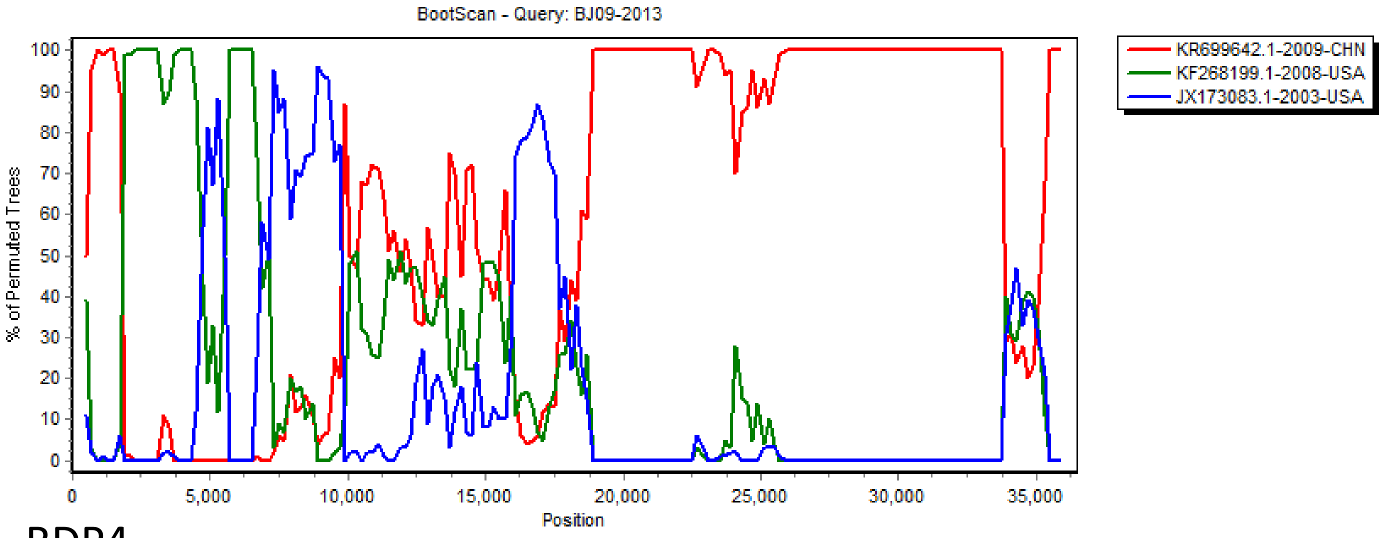

RDP4

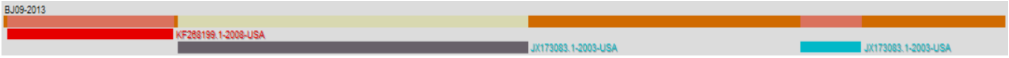

		Breakpoint analysis			
Methods	Average p-value on recombination event	Beginning breakpoint	Ending breakpoint	p value on breakpoint	
				Beginning breakpoint	Ending breakpoint
RDP	$6x10^{-45}$	136	6151	N/A	
GENECONV	$3x10^{-22}$	1989	3301	N/A	
Bootscan	$1x10^{-29}$	1997	3805		
MaxChi	$3x10^{-15}$	137	**6151**	$8x10^{-15}$	$3x10^{-15}$
Chimaera	$3x10^{-12}$	137	6151	$2x10^{-11}$	$3x10^{-12}$
SiScan	$3x10^{-7}$	1997	4925	N/A	
3Seq	$2x10^{-15}$	1341	6216	N/A	

Breakpoint estimated at position 6151

		Breakpoint analysis			
Methods	Average p-value on recombination event	Beginning breakpoint	Ending breakpoint	p value on breakpoint	
				Beginning breakpoint	Ending breakpoint
RDP	$6x10^{-46}$	6333	19011	N/A	
GENECONV	$3x10^{-24}$	7226	13403	N/A	
Bootscan	$3x10^{-16}$	8566	11990		
MaxChi	$5x10^{-18}$	**6333**	**19228**	$8x10^{-7}$	$1x10^{-12}$
Chimaera	$5x10^{-6}$	5391	19011	0.97	$5x10^{-6}$
SiScan	$3x10^{-11}$	6685	18607	N/A	
3Seq	$2x10^{-15}$	4852	17985	N/A	

Beginning breakpoint estimated at position 6333

Ending breakpoint estimated at position 19228

Virus	Number of identical sequences among the 168 analyzed	ORF position	Sequence
BJ04		260	WWREIQFFPIGSHPCTERLFVTYDVETYT
AEF59045.1-Simian adenovirus 20		237	..QS.K...L....RV....L........
ABH01044.1-Simian adenovirus 7	1	248	..Q..S.......AR.Q............
ABK59062.1-Human adenovirus 37		153	..Q...........R..............
CAO78629.1-Human adenovirus 31	1	247	..Q......V....R.....I...I....
ACZ92148.2-Human adenovirus 18		253	..Q...........R.....I........
ACA24377.1-Human adenovirus C	17	154	..............R..............
AAA42478.1-Human adenovirus 12	2	250	..Q..H........R.....I........
BAJ46376.1-Human adenovirus 53	3	153	..Q...........R..............
BAH18775.1-Human adenovirus 8	10	153	..Q...........R..............
0905196A Ad7		841	..Q...........R.....L........
ACO81791.1-Human adenovirus 14		185	..Q...........R.....L........
YP_004300204.1-Simian adenovirus 49	1	263	..Q..S........R..............
ACR78208.1-Human adenovirus 22	2	238	..Q...........R..............
AAS10432.1-Simian adenovirus 24		252	..Q...........R.....L........
ACV41281.1-Human adenovirus 11a		185	..Q...........R.....L........
AEL78833.1-Human adenovirus 62	2	153	..Q...........R..............
AFA46720.1-Human adenovirus 19		238	..Q...........R..............
AP_000576.1-Human adenovirus 35		256	..Q...........R.....L........
ADN06445.1-Human adenovirus 41	1	251	..Q..N........RV.............
BAJ46341.1-Human adenovirus 53	16	153	..Q...........R..............
YP_213966.1-Simian adenovirus 1		248	..Q..S.......AR.Q............
AAS10360.1-Simian adenovirus 22	34	254	..Q...........R.....L........
ADZ39804.1-Simian adenovirus 48	2	234	..QP.K...L....R.....L........
AFD21971.1-Simian adenovirus A1258	6	263	..Q..S........R..............
YP_068023.1-Human adenovirus E	2	255	..Q...........R.....L........
NP_040853.1-Human adenovirus F		251	..QK.N........RV.............
AFD10561.1-Simian adenovirus 18		255	..Q..N...L....R..............
AAW33204.1-Human adenovirus 4	1	255	..K...........R.....L........
YP_001552247.1-Snake adenovirus 1		152	..KP.K.S...ALSNAK...II..I....
YP_006383556.1-Goose adenovirus 4		231	M.QHVR.KCAS.P.NNRN......I....
YP_004414800.1-Raptor adenovirus A		153	Y.ET.S.Q...EN.N.KK..LI..I...S
YP_004935931.1-South polar skua adenovirus-1		154	Y.EG.N.Q...EN.N.KK..LI.....FS
AP_000613.1-Canine adenovirus 2		209	..EK.S.T...AP.N.....IV.......
ACW84422.1-Great tit adenovirus 1		154	Y.ES...Q...ETEN.FK..LV..I....
NP_047384.1-Turkey adenovirus A	1	155	F.ET.P.Q...ENEN.KK..LI..I..F.
AAB05434.1-Canine adenovirus 1	2	208	..EK.S.S...APAN.....IV.......
AEW91332.1-Bovine adenovirus 3	1	82	..E..K...L.A..D.R....V.......
AP_000006.1-Ovine adenovirus A	1	206	..SQ.S.Q....CDD.K.F.L........
YP_094032.1-Bovine adenovirus A		206	..EL.P.Q....YVDIQ...I........
AEK98447.1-Titi monkey adenovirus ECC-2011		219	..Q..K...L.A..D.R............
AP_000026.1-Bovine adenovirus B	1	222	..E..K...WAA..D.R....V.......
YP_068060.1-Tree shrew adenovirus A	1	204	..EP.S...L.AP.E.K..YI........
YP_004123738.1-Murine adenovirus 2		248	..EPVR.Q....PAGIL...I...I....
YP_004782100.1-Bat adenovirus 2		208	..EK.A.A....PAE.....IV..I....
AP_000236.1-Porcine adenovirus C		201	..EN.P.Q.L..YRE....YI........
NP_062435.1-Frog adenovirus 1		150	-.ET.N.S...EPKG.KK..LV..I....
YP_009201.1-Porcine adenovirus A		220	..QT.A.R....PEG.R...L...L....
AEP16409.1-Equine adenovirus 1		222	..ET.S.T....P.-.D...IV.......
NP_077389.1-Bovine adenovirus D		150	..EQ.K.N.V.ALES.K...IV..I....
NP_044702.1-Duck adenovirus A		149	..QP.K.H.V.CVE-.K..Y.V..I....
YP_002822207.1-Murine adenovirus 3		261	..SK.S.S.L..I.S.Q...II..L...C
YP_005271182.1-Bat adenovirus TJM		208	GGKRQPSPLWDLPRKPKGCLLSMTSRPTP
YP_004346921.1-Fowl adenovirus C	1	200	M.QHVH.SCPAQS.HCKQ..L...I....
NP_659515.1-Ovine adenovirus D	1	148	..EK.K.N.V.AIKA-K...IV..I....
NP_015537.1-Murine adenovirus A	1	267	..TK.S.T.L..I.T.K...II..L...C
Consensus 75% from 168 sequences			WWXEIXFFPIGSHPRTERLFXTYDVETYT

Supplementary Figure S4

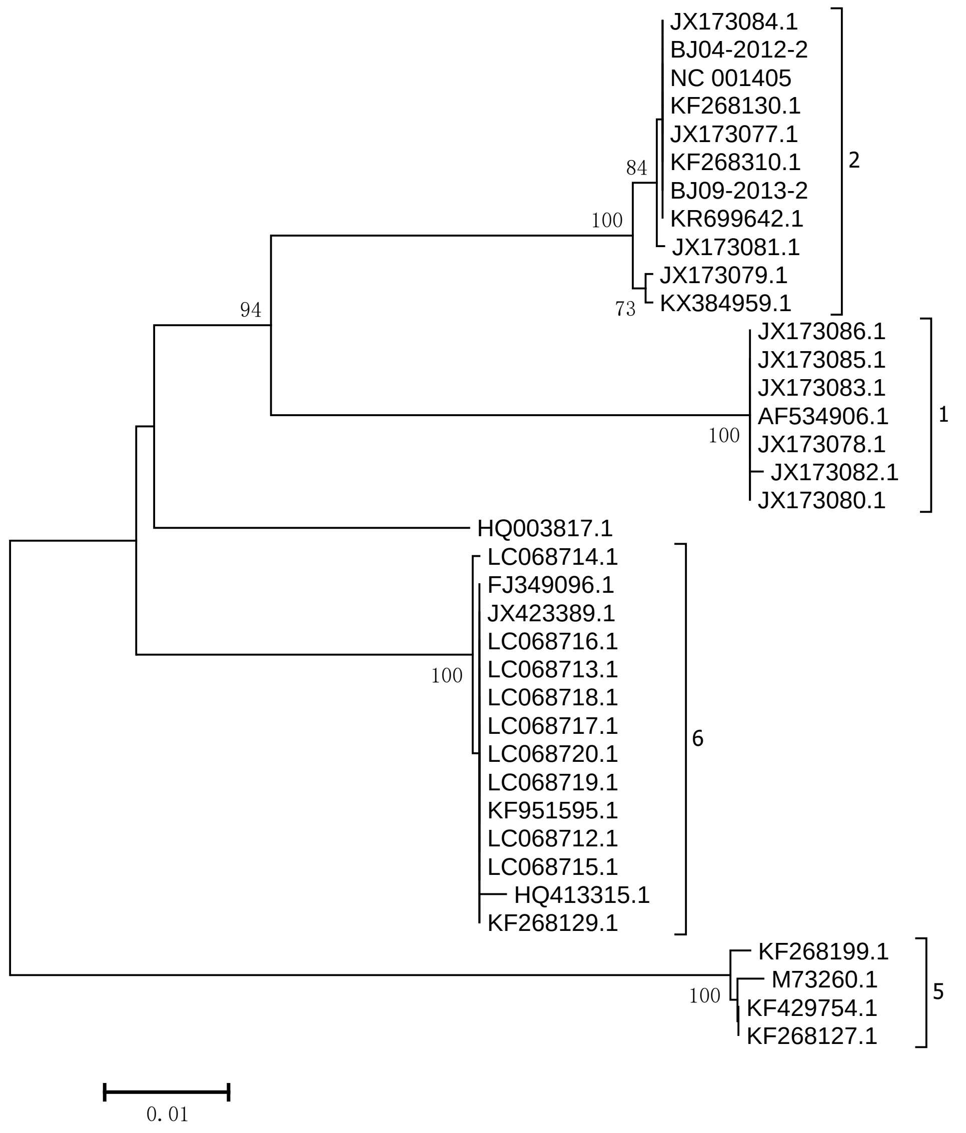

Supplementary Figure S5
